# Clinical Potential of *Curcuma longa* Linn. as Nutraceutical/Dietary Supplement for Metabolic Syndrome: Systematic Review and Meta-Analysis of Randomized Controlled Trials

**DOI:** 10.3390/foods15010060

**Published:** 2025-12-24

**Authors:** Samuel Abiodun Kehinde, Zahid Naeem Qaisrani, Rinrada Pattanayaiying, Bo Bo Lay, Khin Yadanar Phyo, Wai Phyo Lin, Myat Mon San, Nurulhusna Awaeloh, Sasithon Aunsorn, Ran Kitkangplu, Sasitorn Chusri

**Affiliations:** 1 Biomedical Technology Research Group for Vulnerable Populations and School of Health Science, Mae Fah Luang University, Chiang Rai 57100, Thailand; samuelabiodun.research@mfu.ac.th (S.A.K.); qaisrani.research@mfu.ac.th (Z.N.Q.); 6531804115@lamduan.mfu.ac.th (K.Y.P.); 6431804208@lamduan.mfu.ac.th (W.P.L.); 6531804120@lamduan.mfu.ac.th (M.M.S.); 6551811005@lamduan.mfu.ac.th (S.A.); 6451811006@lamduan.mfu.ac.th (R.K.); 2 Biochemical/EnTox Laboratory, Faculty of Basic Medical Sciences, Ajayi Crowther University, Oyo 211001, Nigeria; 3 Department of Chemical Engineering, Faculty of Engineering & Architecture, Balochistan University of Information Technology, Engineering and Management Sciences (BUITEMS), Quetta 87300, Pakistan; 4 Department of Food Innovation and Professional Chef, Faculty of Science and Technology, Suan Sunandha Rajabhat University, Bangkok 10300, Thailand; rinrada.pa@ssru.ac.th; 5 School of Information Technology, Mae Fah Luang University, Chiang Rai 57100, Thailand; 6431306113@lamduan.mfu.ac.th; 6 Thai Faculty of Allied Health Science, Nakhon Ratchasima College, Nakhon Ratchasima 30000, Thailand; nurulhusna@nmc.ac.th

**Keywords:** *Curcuma longa*, curcumin, metabolic syndrome, type 2 diabetes, inflammation, lipid profile, antioxidant, randomized controlled trial, nutraceuticals

## Abstract

Metabolic syndrome (MetS) and its associated conditions, namely, type 2 diabetes mellitus (T2DM), non-alcoholic fatty liver disease (NAFLD), obesity, and polycystic ovary syndrome (PCOS) are characterized by insulin resistance, dyslipidemia, and low-grade inflammation. Curcumin, a polyphenolic compound derived from *Curcuma longa* Linn., exhibits pleiotropic metabolic and anti-inflammatory properties and has thus been evaluated as a nutraceutical intervention for these conditions, but findings remain inconsistent. This systematic review and meta-analysis evaluated the clinical efficacy of *Curcuma longa* supplementation on anthropometric, glycemic, lipid, inflammatory, and oxidative stress parameters in adults with MetS or related disorders. A comprehensive search of databases (PubMed, Scopus, AMED, LILACS, and Google Scholar) identified 104 eligible randomized controlled trials (RCTs). The included trials primarily assessed standardized oral turmeric/curcumin supplements and bioavailability-enhanced formulations rather than whole culinary turmeric. Pooled standardized mean differences (SMDs) with 95% confidence intervals (CIs) were computed using random-effects models. Subgroup analyses were conducted by disease category, dose, and formulation. Risk of bias was assessed using the Cochrane RoB 2 tool. Curcumin supplementation significantly reduced fasting blood sugar (SMD = −0.54, 95% CI −0.72 to −0.36) and HbA1c (SMD = −0.41, 95% CI −0.60 to −0.23) in T2DM; decreased triglycerides (SMD = −0.48; 95% CI: −0.70 to −0.25), and LDL cholesterol (SMD = −0.39; 95% CI: −0.59 to −0.18) while elevating HDL cholesterol (SMD = 0.45; 95% CI: 0.25 to 0.65) and total antioxidant capacity (SMD = 0.73; 95% CI: 0.51 to 0.94). *Curcuma longa* also attenuated systemic inflammation, lowering C-reactive protein (SMD = −0.62; 95% CI: −0.81 to −0.43), TNF-α (SMD = −0.57; 95% CI: −0.80 to −0.34), and IL-6 (SMD = −0.50; 95% CI: −0.70 to −0.29). Heterogeneity was moderate-to-high, reflecting some differences in the formulation, dosage, and duration. Collectively, these findings affirm that *Curcuma longa* exerts measurable, clinically relevant improvements on glycemic regulation, lipid metabolism, and inflammatory−oxidative balance, supporting its role as a nutraceutical adjunct in metabolic health management, while its bioavailability-enhanced formulations show superior efficacy. Larger, long-term, multicenter RCTs are warranted to confirm durability, optimal dosing, and safety.

## 1. Introduction

Metabolic syndrome (MetS) induces a constellation of cardiometabolic risk factors such as central obesity, dyslipidemia, poor glycemic control, and hypertension that increase the risk of type 2 diabetes, cardiovascular disease, and non-alcoholic fatty liver disease [1,2,3]. The rate of its spread is increasing across the globe due to increases in urbanization, sedentary lifestyles, and high-energy diets. One in every four adults qualifies as experiencing MetS; this places an extra burden on health systems that are already under pressure from non-communicable diseases [4]. MetS is particularly dangerous, since each of its components support each other. High visceral fats lead to dysfunction in the adipocytes, releasing pro-inflammatory cytokines like TNF-α and IL-6 and reducing beneficial adipokines like adiponectin [5]. This inflammatory condition disrupts insulin signaling, drives oxidative stress, compromises endothelial activity, and disrupts lipid metabolism. These changes, in combination, cause insulin resistance, atherosclerosis, and organ damage. Thus, multi-tasking treatments targeting inflammation, oxidative stress, and metabolic dysregulation are highly promising for the treatment of MetS.

A traditional medicinal ingredient and a culinary spice that has been used for centuries is Turmeric (*Curcuma longa* Linn.). It contains antioxidant, anti-inflammatory, and metabolism-regulating properties due to its main active compounds, curcumin and curcuminoids [2,6,7]. In preclinical models, curcumin has been shown to cause an inhibition in the activation of NF-κB, downregulate proinflammatory mediators such as COX-2 and iNOS, stimulate AMP-activated protein kinase (AMPK) signaling, inhibit lipid peroxidation, and enhance mitochondrial function [8,9]. Beyond these, a growing body of research suggests curcumin also modulates gut microbiota composition, strengthens intestinal barrier integrity, and invariably influences host–microbiome metabolic crosstalk [10,11,12].

Over the past decade, a considerable number of randomized controlled trials (RCTs) have been carried out on individuals with MetS or related metabolic diseases. Some trials have described an increase in fasting glucose, HbA1c, triglycerides, total cholesterol, and inflammatory markers [1,2,13]. Others have reported negligible or inconsistent effects. These discrepancies may arise from the differences in study design, dosage, curcumin formulation, intervention duration, or baseline metabolic status. Consequently, the overall clinical relevance of *C. longa* in metabolic regulation remains uncertain.

Metabolic syndrome presents a major clinical and public health challenge because its management often requires long-term, multi-drug therapy targeting individual components of the disease, leading to polypharmacy, variable adherence, and cumulative adverse effects. This has driven increasing interest in safe, cost-effective adjunctive approaches capable of simultaneously modulating key pathogenic mechanisms, including chronic low-grade inflammation, oxidative stress, insulin resistance, and dyslipidemia. Nutraceuticals derived from bioactive food constituents have therefore gained attention as integrative strategies for metabolic risk reduction, provided their clinical efficacy is supported by robust randomized evidence.

*Curcuma longa* Linn., particularly its principal bioactive compound, curcumin, has emerged as a leading nutraceutical candidate due to its pleiotropic anti-inflammatory, antioxidant, and metabolic regulatory effects. Advances in formulation technologies have improved its bioavailability and clinical applicability; however, findings from randomized controlled trials investigating metabolic syndrome and related conditions remain heterogeneous and at times, inconsistent. Accordingly, the present systematic review and meta-analysis quantitatively synthesizes randomized evidence to clarify the clinical relevance of *Curcuma longa* supplementation across metabolic, inflammatory, and oxidative stress outcomes, thereby providing evidence to inform its rational integration into metabolic health management.

Given this heterogeneity, a rigorous quantitative synthesis of relevant data is needed to clarify the therapeutic potential of *Curcuma longa* as a dietary supplement in the aforementioned metabolic disorders. Although turmeric has a long history of culinary and traditional medicinal use and can be considered a nutraceutical, most contemporary randomized clinical trials evaluate standardized turmeric/curcumin supplements or bioavailability-enhanced curcumin formulations rather than whole-food turmeric consumed as part of a habitual diet. Accordingly, this review focuses specifically on supplemental and formulated curcumin interventions and not on the dietary use of whole turmeric. Therefore, this systematic review and meta-analysis aims to evaluate the pooled effects of *C. longa* supplementation on glycemic, lipid, anthropometric, inflammatory, and oxidative stress markers in adults with MetS or related conditions; explore subgroup differences by disease type, dosage, and formulation; and assess the quality and consistency of the available clinical evidence. Establishing a consolidated evidence base for curcumin’s efficacy may inform its rational integration into dietary and preventive strategies for improved metabolic health.

## 2. Materials and Methods

### 2.1. Study Design and Protocol Registration

**Figure 1:**
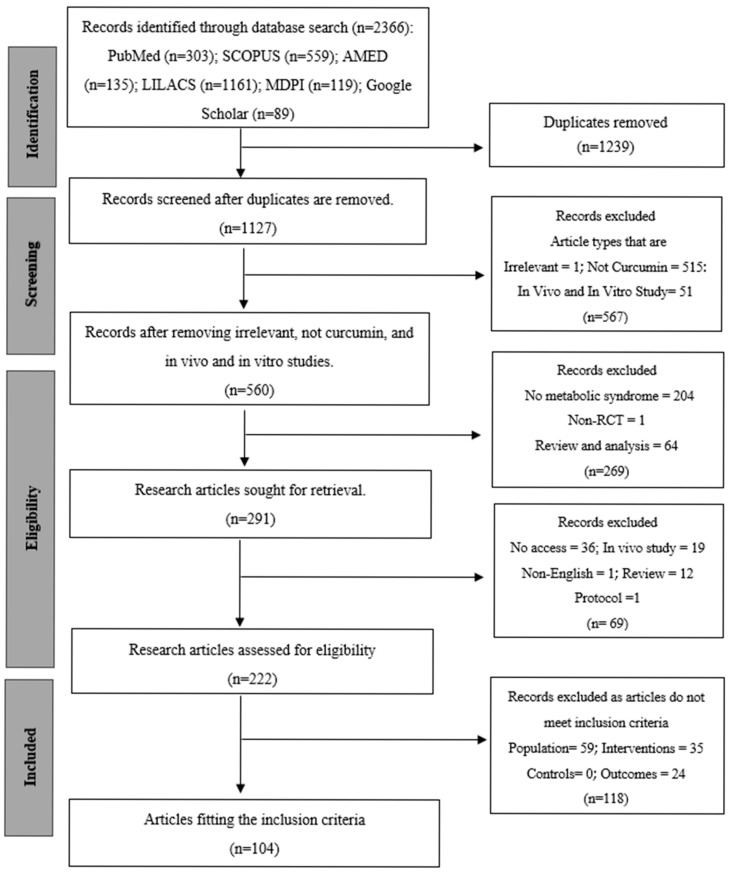
PRISMA flow diagram of the systematic review process.

The systematic review and meta-analysis were performed according to the Preferred Items to Systematic Reviews and Meta-Analyses (PRISMA) 2020 guidelines [14]. Figure 1 demonstrates the PRISMA checklist for every reporting domain. The a priori registration of the study protocol is in the International Prospective Register of Systematic Reviews (PROSPERO) with the registration number CRD420251184355. The study was aimed at assessing the clinical effectiveness of *Curcuma longa* Linn. (curcumin) supplementation in metabolic syndrome and associated conditions, such as type 2 diabetes mellitus (T2DM), non-alcoholic fatty liver disease (NAFLD), obesity, and polycystic ovary syndrome (PCOS). The inclusion criteria were randomized controlled trials (RCTs) that investigated *Curcuma longa* or curcumin preparations in the oral form, either as a mono-therapy or an adjunctive to standard care, and reported an outcome on anthropometric, glycemic, lipid, inflammatory, or oxidative stress measurements. MetaAnalysisOnline.com (https://www.metaanalysisonline.com; accessed 15 July 2025) [15] was used to conduct a meta-analysis of the extracted quantitative data. The disease subtype and outcome categories were used to stratify the pooled analyses in order to identify heterogeneity. The effects were measured as standardized mean differences (SMDs) in the form of 95% confidence intervals (CIs) based on a random-effects model. The tau^2^ and I^2^ statistics were used to assess between-study variance. Sensitivity and subgroup analyses were performed to determine the effect of the study quality, intervention formulation (standard, piperine-enhanced, phospholipidated, or nanocurcumin), and the duration of dosage on the overall effects.

### 2.2. Information Sources and Search Strategy

An extensive literature review was performed to explore the existing randomized controlled trials (RCTs) exploring the impact of *Curcuma longa* Linn. (curcumin) supplementation on the elements of metabolic syndrome and its associated conditions. Also, databases such as Pubmed (http://www.pubmed.gov (accessed on 23 August 2024)), Scopus (http://www.scopus.com (accessed on 28 August 2024)), AMED (https://www.ebsco.com/products/research-databases (accessed on 28 August 2024)), LILACS (https://lilacs.bvsalud.org (accessed on 30 August 2024)), MDPI (www.mdpi.com (accessed on 28 August 2024)), and Google Scholar (www.scholar.google.com (accessed on 2 September 2024)) without time restrictions (Supplementary Table S1). Searches were conducted from database inception through 9 September 2024. No additional update searches were performed after 9 September 2024, prior to final analysis, to avoid selective inclusion and to maintain methodological consistency. The search strategy was a combination of Medical Subject Headings (MeSH) and the free-text terms of Curcuma longa, curcumin, and metabolic syndrome. Sensitivity and specificity were improved with the use of the Boolean operators (“AND” or “OR”) and truncation symbols. All databases had the search strategies adapted to them, making them sensitive and specific. There were also no limitations on the year of publication. Only English studies that were carried out on human populations were considered. The retrieved articles and relevant reviews were manually screened to discover more eligible studies in their reference lists. To ensure search transparency and reproducibility, the complete database search history and query strings are provided in Supplementary Table S1. The search process and record selection are visually summarized in the PRISMA flow diagram (Figure 1)

### 2.3. Eligibility Criteria

For the purposes of this review, we defined ‘Nutraceutical/Dietary Supplement’ as an oral, standardized preparation delivered as a capsule, tablet, or liquid formulation containing curcumin or curcumin-enriched Curcuma longa extracts. This definition includes formulations designed to enhance systemic bioavailability (e.g., curcumin–piperine combinations, phytosomal or phospholipid complexes, and nano-formulations). Studies of whole-food turmeric preparations incorporated into meals or habitual diets were not the focus of this review. This distinction is critical because supplement formulations provide concentrated, standardized curcuminoid doses and pharmacokinetic profiles that differ substantially from culinary turmeric. We included studies based on the following predefined eligibility criteria, which were structured according to the PICOS framework (Table 1).

#### 2.3.1. Exclusion Criteria

Studies that were not randomized (for example, observational studies), in vitro experiments, and animal studies were excluded because they do not provide the randomized clinical evidence required for this review. We also excluded case reports, narrative reviews, editorials, and conference abstracts lacking full text, as these sources do not present extractable trial data. Trials that administered curcumin together with other herbal extracts or multi-ingredient formulations were excluded when curcumin-specific effects could not be disentangled. Finally, we excluded trials that did not apply clear diagnostic criteria for metabolic syndrome or that failed to report sufficient outcome data for extraction and synthesis.

#### 2.3.2. Prognostic Criteria for Inclusion

To ensure consistency, eligible studies must report baseline prognostic indicators of MetS such as waist circumference, BMI, or measures of central obesity; blood pressure (systolic/diastolic); lipid profile (triglycerides, HDL-C, LDL-C, total cholesterol); glycemic control (fasting plasma glucose, HbA1c, insulin resistance indices); and presence of at least three diagnostic risk factors for MetS (per ATP III, IDF, or WHO). These baseline criteria allow for pooling of comparable populations and enable subgroup analyses based on the severity of metabolic dysfunction, geographic region, or curcumin formulation.

### 2.4. Data Retrieval and Synthesis

Two independent reviewers screened titles and abstracts. Potentially eligible studies were identified, and the full contents were strictly examined according to the predetermined inclusion and exclusion criteria. Where the effect sizes were not extractable or calculable based on the published data, the relevant authors were contacted through email to obtain further statistical data. Disputes were resolved by consensus or a third reviewer. Only manuscripts describing original clinical studies were considered. All articles found during the search were imported into Microsoft Excel 2019, and duplicates were eliminated. The first screening included a title and abstract review, which led to the exclusion of irrelevant articles. The rest of the records underwent complete-text critical appraisal. Articles that had no complete text or were irrelevant were eliminated.

In order to improve data visualization, results were arranged and represented in figures, with other data summarized in tables. A standardized form of data extraction was created and pilot tested. Three reviewers extracted data from the selected studies. Variables of interest were as follows: study characteristics (author(s), year of publication, country, study design, sample size, randomization method, and blinding), population characteristics (age, gender distribution, duration and severity, baseline MetS symptoms), intervention (name of curcumin formulation, composition, form, route of administration, frequency, duration, dosage), comparator (type of control, e.g., no treatment, standard care/control diet, placebo), and outcomes assessed as core components of MetS, such as fasting blood glucose, HbA1c, triglycerides, total cholesterol, HDL-C, LDL-C, systolic and diastolic blood pressure, waist circumference, inflammatory markers (CRP, TNF-a, IL-6), oxidative stress markers (MDA, TAC), insulin resistance indices (HOMA-IR), liver enzymes, and body mass index (BMI). Any difference between data extraction was sorted out by discussion.

### 2.5. Quality Assessment Risk of Bias Assessment

The risk of bias in each included study was independently assessed by three (3) reviewers via predefined criteria adapted from the Cochrane Risk of Bias 2 (RoB 2) to assess the studies’ risk of bias [16]. This evaluation examined various factors, including the risk of bias in individual studies, the directness of the evidence, the precision of effect estimates, the heterogeneity among studies, and the potential for publication bias. Bias, in this context, refers to systematic deviations from accurate findings or inferences that can distort study results. This instrument comprises items categorized into randomization, allocation concealment, baseline characteristics, patient blinding, caregiver blinding, blinding of outcome assessor, overall attrition, differential attrition, other bias, and overall bias. Each domain was evaluated using one of three responses: “Low” (indicating low risk of bias), “High” (indicating high risk of bias), or “Unclear” (not clear due to insufficient information). Two reviewers independently evaluated each study and classified the risk of bias as “low,” “unclear,” or “high” in each domain. Upon concluding their assessments, the reviewers engaged in a comparative analysis and discussion of their findings. Discrepancies in scoring were addressed through consultation with a third reviewer to achieve consensus.

### 2.6. Statistical Analysis

Quantitative synthesis (meta-analysis) was performed using an online tool, MetaAnalysisOnline.com (https://metaanalysisonline.com; accessed 15 July 2025) when sufficient homogeneity in study design, intervention, and outcome measures was present. The intervention and control group sample sizes, standard deviations (SDs), and mean values were immediately uploaded into the application. Before data entry, the standard errors (SEs) provided in the studies were converted to standard deviations (SDs). Continuous outcome analysis was conducted via a random effects model because of the expected methodological and biological heterogeneity of studies. Effect sizes were calculated using the standard mean difference (SMD) with a 95% confidence interval (CI) due to the various measurement scales that were used in the study. The method of moments (Der-Simonian and Laird) was used to determine the variation between studies, and heterogeneity was measured using the Cochran Q test and quantified with the I^2^ statistic. An I^2^ value of more than 50% meant that there was a lot of heterogeneity. The assessment of biases in the publications was carried out visually (using funnel plots) and statistically (using the Egger regression test) [15]. A *p*-value of less than 0.05 was considered a manifestation of an important asymmetry. Publication bias was evaluated both visually through funnel plots and statistically through the Egger regression test [15], which was developed on MetaAnalysisOnline.com. The overall quality of evidence obtained for each outcome was evaluated via the Grading of Recommendations, Assessment, Development, and Evaluations (GRADE) framework [17]. The domains assessed included risk of bias, inconsistency, indirectness, imprecision, and publication bias. The quality of the evidence was categorized as high, moderate, low, or very low.

## 3. Results

### 3.1. Study Selection

The process of study selection is represented in the PRISMA flow diagram (Figure 1). As of 9 September 2024, a total of 2366 records were first retrieved via electronic database searches, comprising contributions from PubMed (*n* = 303), SCOPUS (*n* = 559), AMED (*n* = 135), LILACS (*n* = 1161), MDPI (*n* = 119), and Google Scholar (*n* = 89). Following the elimination of 1239 duplicate records, 1127 distinct records were retained for screening. In the title and abstract screening step, 567 records were rejected because they did not meet the inclusion criteria. The exclusions were mainly attributed to the articles being reviews, nonclinical outcome measures, non-metabolic syndrome, non-curcumin, including animal or in vitro data, or lacking a comparative arm. This resulted in 291 records for additional evaluation. A total of 69 articles were eliminated because they were non-English, irrelevant, or lacked full-text access after a comprehensive assessment, resulting in 222 articles being evaluated for eligibility. After a comprehensive evaluation, 118 additional publications were removed for failing to meet the specified inclusion criteria: 59 did not have the required population, 35 did not satisfy the intervention criteria, and 24 lacked outcome data. Finally, 104 studies [18,19,20,21,22,23,24,25,26,27,28,29,30,31,32,33,34,35,36,37,38,39,40,41,42,43,44,45,46,47,48,49,50,51,52,53,54,55,56,57,58,59,60,61,62,63,64,65,66,67,68,69,70,71,72,73,74,75,76,77,78,79,80,81,82,83,84,85,86,87,88,89,90,91,92,93,94,95,96,97,98,99,100,101,102,103,104,105,106,107,108,109,110,111,112,113,114,115,116,117,118,119,120,121] were included in the final systematic review and fulfilled all the set inclusion criteria. These studies were identified by searching for statistical data, either in published publications or by emailing the corresponding author. Articles that had insufficient information to be used in meta-analysis were only included in the qualitative section of the systematic review. The earliest publication was from 2011, and over 90% of studies published were performed in the last decade (2014–2024).

### 3.2. Assessment of the Risk of Bias of the Included Studies

**Figure 2:**
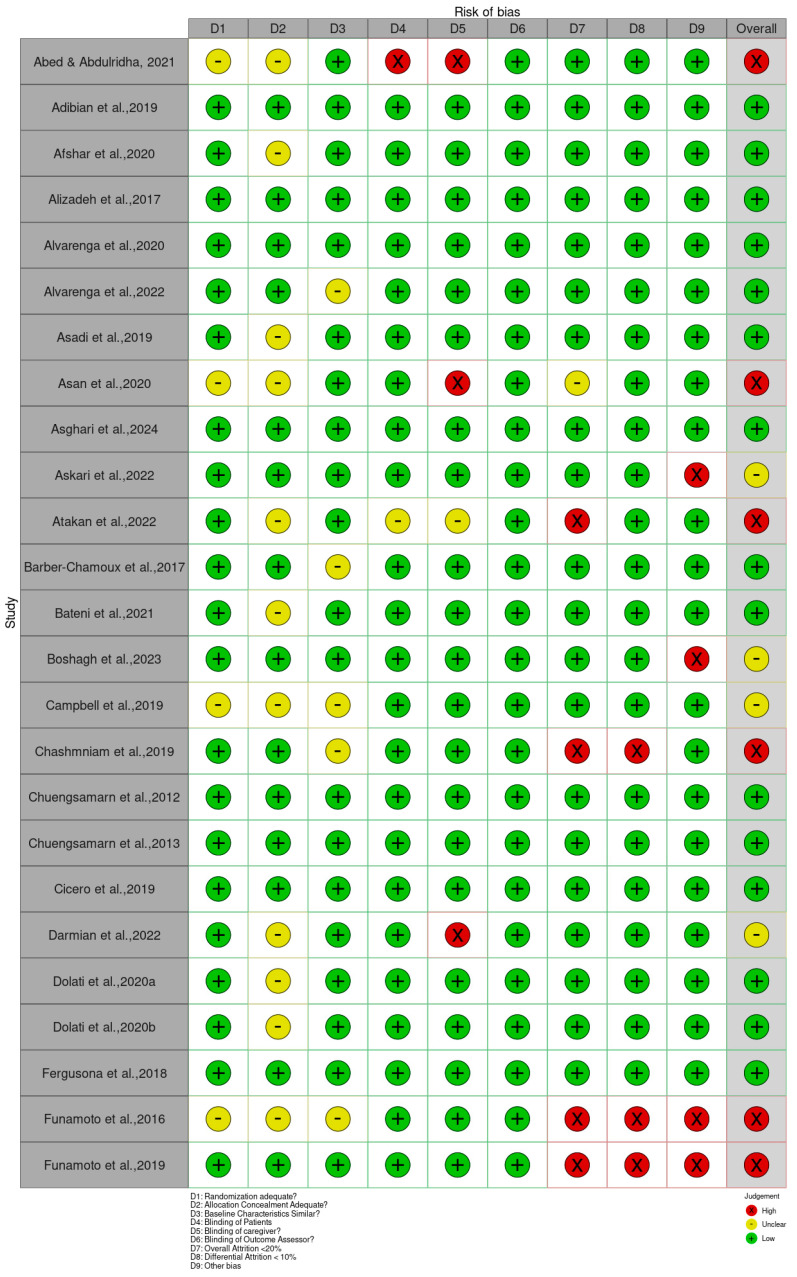

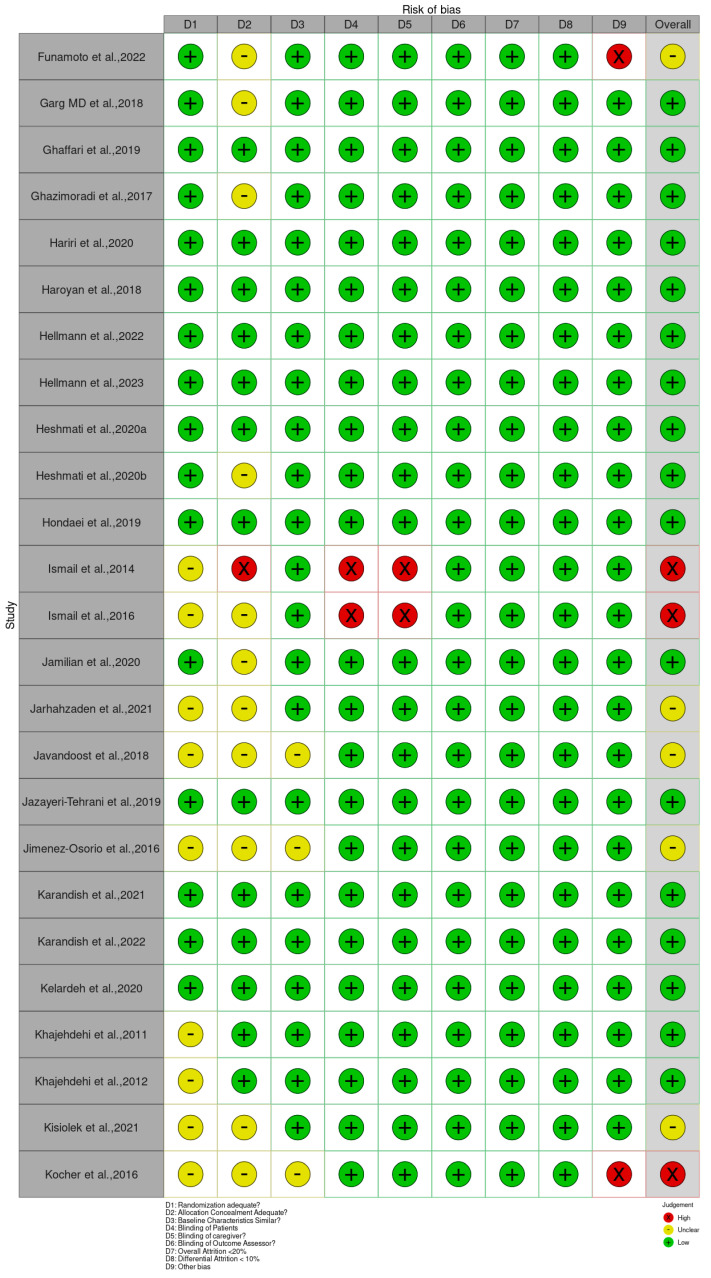

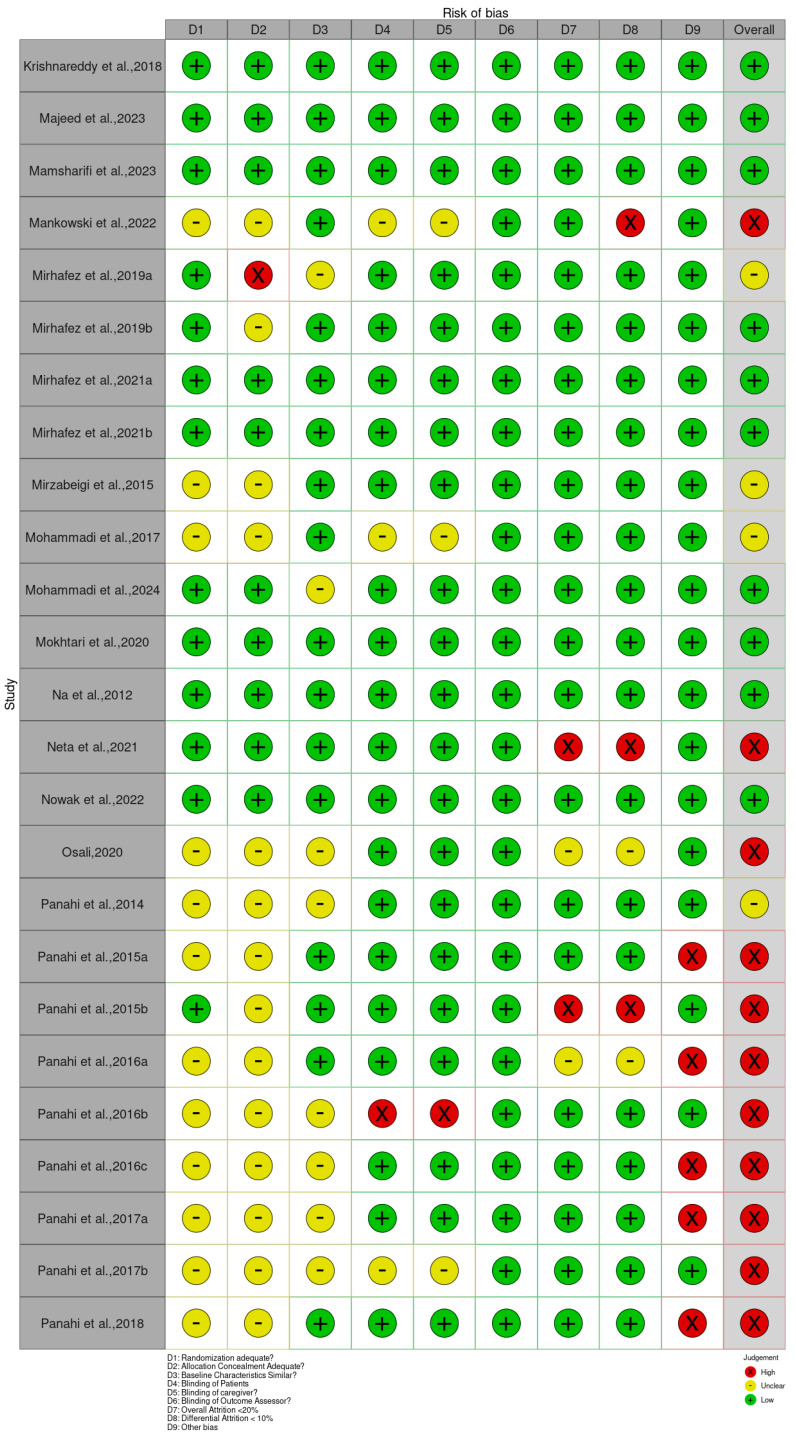

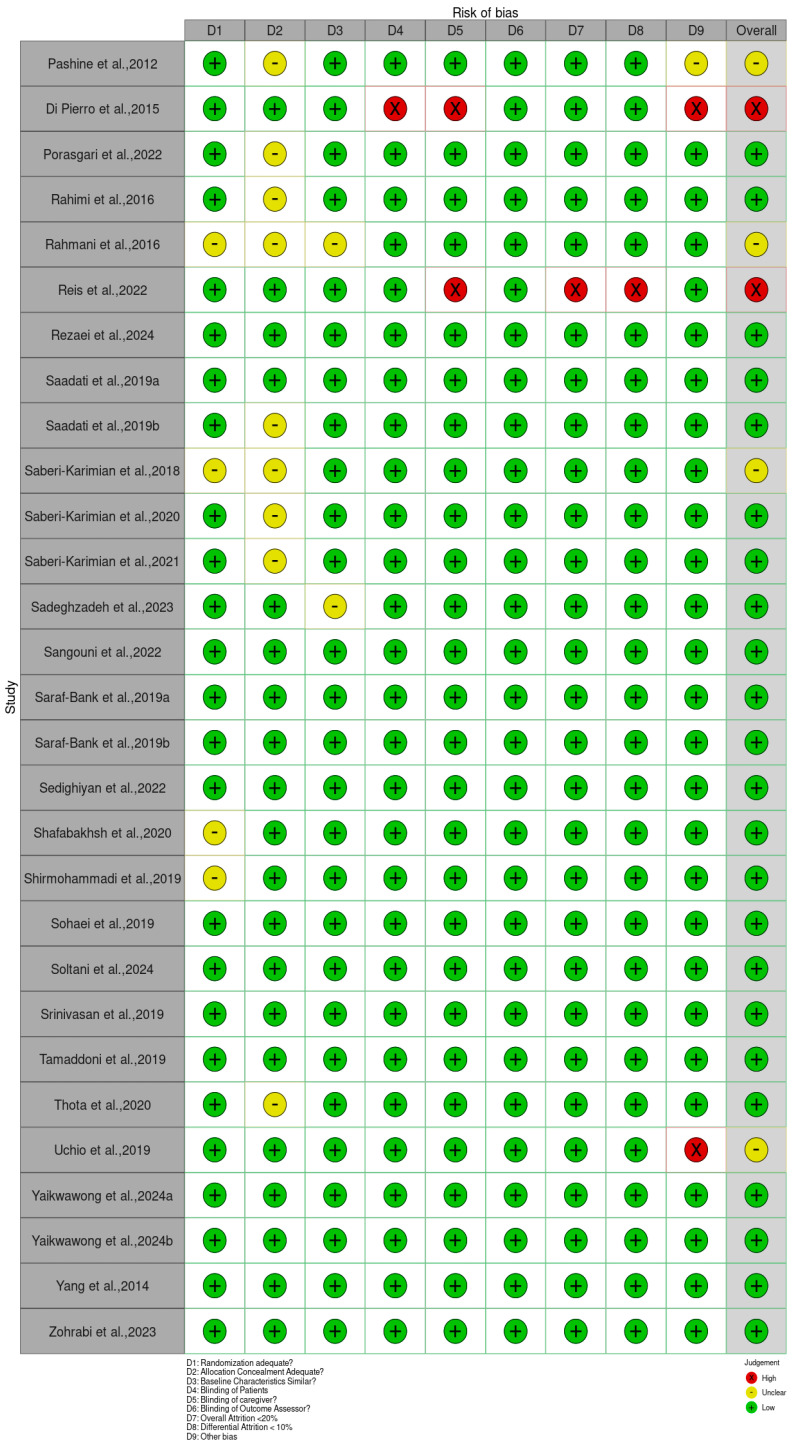

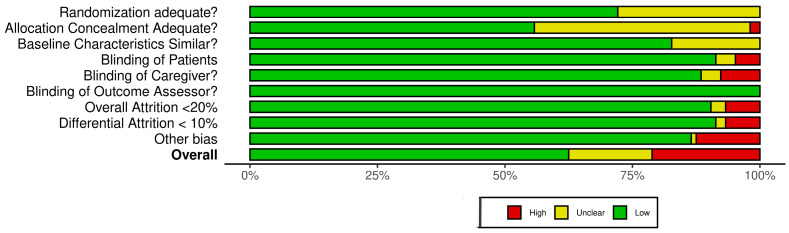
Quality of reporting and bias evaluation conducted with the risk of bias tool. The upper panel illustrates the quality of reporting and bias risk in the included studies, while the lower panel evaluates biases related to selection, performance, detection, attrition, and other factors.

The Cochrane Risk of Bias 2 (RoB 2) tool for RCTs was applied to all included studies to evaluate their methodological quality. The assessment revealed a range of bias levels across domains (bias arising from the randomization, allocation concealment, baseline characteristics, patient blinding, caregiver blinding, blinding of outcome assessor, overall attrition, differential attrition, other bias, and overall bias) (Figure 2 and Supplementary Table S2). Overall, the risk of bias assessment indicates that a substantial majority of the included randomized controlled trials (RCTs) involving *Curcuma longa* are considered to have a low risk of bias in domains related to blinding and the completeness of outcome data. Specifically, risks associated with the Blinding of Patients, Blinding of Caregivers, and Blinding of Outcome Assessors were judged to be low across almost all studies, suggesting robust implementation of double-blind methodologies. Furthermore, the domains addressing Attrition Bias, represented by both Overall Attrition <20% and Differential Attrition <10%, also showed minimal concern, confirming that participant dropout and differential loss between groups were generally well-managed or reported effectively. This consistency across blinding and attrition domains strengthens the reliability of the observed treatment effects.

Bias arising from the randomization process was generally low, as many studies clearly reported adequate random sequence generation and allocation concealment [34,36,47,49,80]. However, a few trials, such as Abed et al. [19], Asan et al. [25], and Ismail et al. [54], showed some concerns or high risk due to insufficient reporting or unclear randomization procedures. Bias due to deviations from intended interventions showed the greatest variability. While studies such as Haroyan et al. [48], Hellmann et al. [50], and Mirhafez et al. [75] adequately implemented blinding and adherence monitoring, others, including Ismail et al. [54,55] and several trials by Panahi et al. [85,87], were rated as having some concerns or high risks, primarily because of open-label designs and potential protocol deviations.

Bias due to missing outcome data was mostly low across studies, with manageable attrition rates reported in trials such as Adibian et al. [19], Chuengsamarn et al. [35], and Cicero et al. [36]. Nevertheless, incomplete or unclearly handled data in studies such as Abed et al. [18], Asan et al. [25], and Mankowski et al. [71] resulted in the determination of there being some concern for this domain. Bias in measurement of the outcome was predominantly low in trials using objective biochemical or anthropometric measures [34,47,49,74]. In contrast, studies that relied on subjective outcomes without assessor blinding, such as Funamoto et al. [41,42], Chashmniam et al. [33], and Neta et al. [81], were rated as high or having some risks for outcome measurement. Bias in the selection of the reported result was low in most trials, indicating consistent reporting of prespecified outcomes [35,47,50,75]. A few studies, particularly those lacking a published protocol or showing incomplete reporting of secondary outcomes, were marked as having some concerns [85,87].

Overall, most trials demonstrated a low or moderate risk of bias, supporting confidence in the evidence base. High-risk studies [25,28,33,41,42,54,55,67,71,81,83,85,86,87,88,89,90,91,92,94,98] tended to show combined weaknesses in randomization, blinding, and outcome assessment. In contrast, a large number of studies [19,20,21,22,23,24,26,29,30,34,35,36,38,39,40,44,45,46,47,48,49,50,51,52,53,56,59,61,62,63,64,65,68,69,70,73,74,75,78,79,80,82,95,96,99,100,101,103,104,105,106,107,108,109,110,111,112,113,114,115,116,118,119,120,121] consistently demonstrated low risk across domains, contributing the most reliable evidence in this review.

### 3.3. Study Characteristics

A total of 104 randomized controlled trials (RCTs) investigating the clinical effects of *Curcuma longa* Linn. or its formulations in populations related to metabolic syndrome were included in this review. The included trials were published between 2011 and 2024, highlighting a steady and global rise in curcumin-focused clinical research over the past decade. The general characteristic table is illustrated in Table 2.

#### 3.3.1. Publication Year

**Figure 3:**
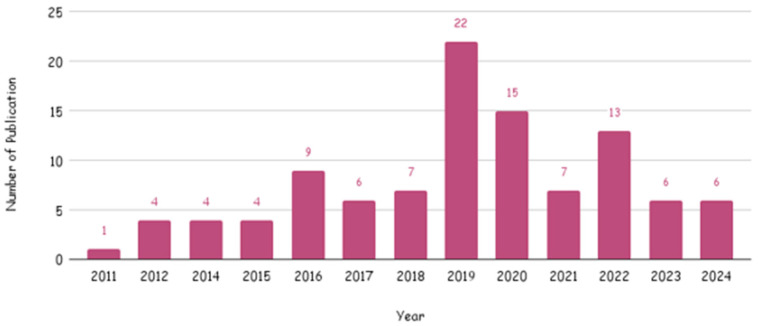
Number of publications per year of included studies.

The temporal distribution of studies revealed a sharp increase in research output after 2015. Only about 12.5% (*n* = 13) of the studies were published between 2011 and 2015 [34,35,54,64,65,76,80,84,85,86,93,94,120] while 81.7% (*n* = 85) were published between 2016 and 2023 [18,19,20,21,22,23,24,25,27,28,29,30,31,32,33,36,37,38,39,40,41,42,43,44,45,46,47,48,49,50,51,52,53,55,56,57,58,59,60,61,62,63,66,67,68,69,70,71,72,73,74,75,77,79,81,82,83,87,88,89,90,91,92,95,96,97,98,100,101,102,103,104,105,106,107,108,109,110,111,112,114,115,116,117,121]. The remaining 5.76% (*n* = 6) were published in 2024, demonstrating sustained and accelerating interest in curcumin as a supplement and nutraceutical ingredient for metabolic health. The year intervals used for reporting publication frequency (2011–2015, 2016–2023, and 2024) were chosen to reflect the empirically observed inflection in the temporal distribution of included trials that occurs after 2015 (Figure 3).

#### 3.3.2. Demographic Distribution

**Figure 4:**
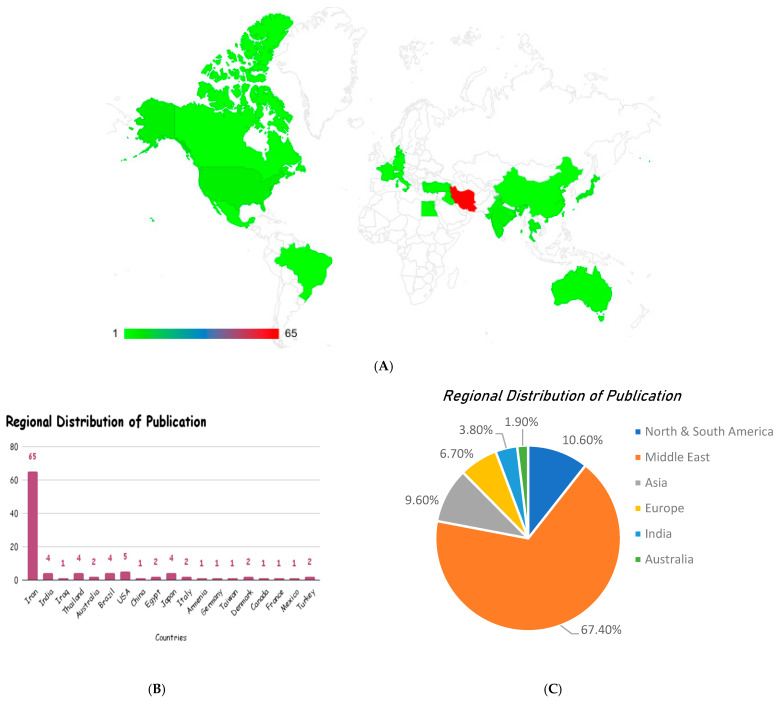
Geographical distribution (**A**) and regional distribution of the publication (**B**,**C**) of the included studies, reflecting the demographics of the studies assessing the beneficial effects of curcumin/*Curcuma longa* extracts in research on metabolic syndrome.

The studies were conducted in nineteen (19) countries, with Iran as a leading country, with 63% (*n* = 65) of the studies, e., refs. [73,74,78,84,97] followed by the USA (*n* = 5). There were 4 studies each from India, Thailand, Brazil, and Japan, and 2 studies each from Australia, Egypt, Italy, Denmark, and Turkey, while other countries like Iraq, China, Armenia, Germany, Taiwan, Canada, France, and Mexico had only 1 study each (Figure 4). The selected studies were published between 2001 and 2024. The year 2019 had the highest number of publications [21, 20.2%], while the years 2016–2024 each had six or more publications. The regional distribution of publications shows a marked dominance of research output from the Middle East (67.4%), indicating a strong regional interest and investment in *Curcuma longa* research. North and South America contributed 10.6%, while Asia followed closely with 9.6% of studies. Europe accounted for 6.7%, India for 3.8%, and Australia for 1.9%. This distribution highlights limited global representation, with most of the evidence emerging from the Middle East, suggesting potential geographical bias and also the need for broader international research participation to enhance the generalizability of these findings.

### 3.4. Population Characteristics

**Figure 5:**
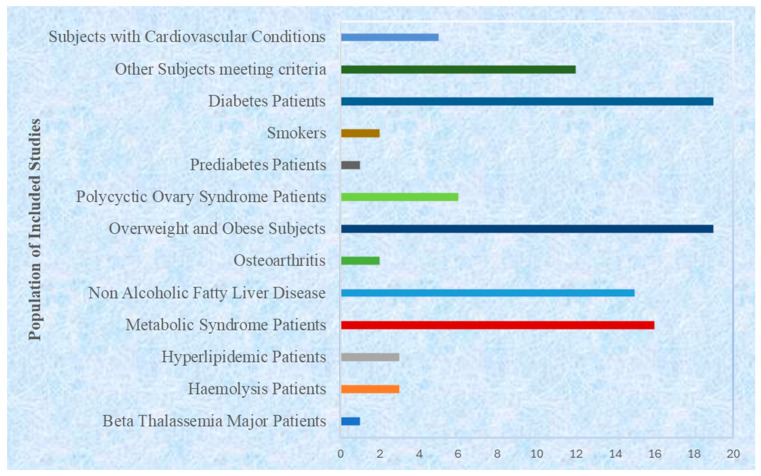
Populations of included studies with reference to associated diseases.

The target populations were diverse and were unified by their metabolic relevance (Figure 5). About 70% (*n* = 73) of the studies targeted patients diagnosed with metabolic syndrome or its main elements, which included type 2 diabetes, dyslipidemia, obesity, or non-alcoholic fatty liver disease, e.g., refs. [75,80,85,96]. Approximately 18% (*n* = 19) of the studied patients had associated metabolic or inflammatory conditions like chronic kidney disease (60), ischemic stroke (18, 31), osteoarthritis (85), or hypertension (43). The remaining 12 and 0 percent (*n* = 12) were seemingly healthy but overweight or obese adults, as a measure of curcumin’s preventive efficacy, e.g., refs. [32,36,49]. This pattern shows that the majority of these research studies were performed on a population that is already presenting with metabolic dysfunctions or risk factors.

#### 3.4.1. Gender Distribution

**Figure 6:**
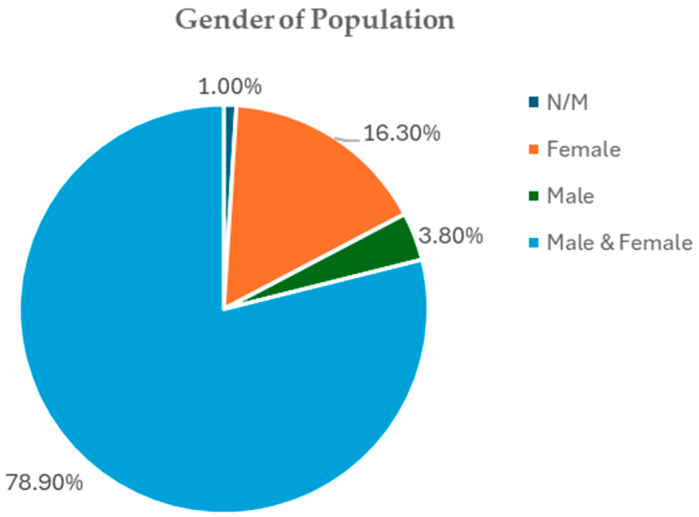
Gender distribution of included studies.

Out of the study populations used in the review, 78.9% of studies had both male and female participants, thus making them more generalizable, e.g., refs. [47,73,74,87]. Another 16.3% of the trials had only female subjects, with most of them addressing a reproductive or hormonal disorder, including polycystic ovary syndrome [25,52,56,121], postmenopausal health [105], or postmenopausal obesity in women [38,95]. A very small minority of trials used only male subjects (3.8%) and generally focused on male infertility or exercise metabolism [21,32]. The gender of the participants was not specified in a small percentage (1%) (Figure 6). This distribution shows that most randomized controlled trials that have measured the clinical impact of *Curcuma longa* on metabolic syndrome have mixed-gender sample designs.

#### 3.4.2. Age of Participants

The age of the participants ranged between 15 and 79 years, with the majority of studies centered on adults of middle age. Approximately 76% (*n* = 79) of studies enrolled adults aged between 35 and 60 years, the age at which metabolic syndrome is most impacted [75,80,85]. Adolescents or younger adults were represented in about 10% of the studies (*n* = 10), such as Ismail et al. [54], Saraf Bank et al. [107], and Campbell et al. [32]. The rest 14% (*n* = 15) were older adults with ages above 65 years, including the articles by Mankowski et al. [71], Funamoto et al. [42], and Kelardeh et al. [63]. This distribution illustrates a strong clinical emphasis on the adult and aging population, where curcumin’s metabolic and anti-inflammatory actions are most relevant.

#### 3.4.3. Sample Size

The studies that were included had diverse numbers of participants. Small-scale trials (<40 participants) represented 31% (*n* = 32) of the total, primarily serving as pilot or mechanistic investigations [29,38,50]. Trials of moderate size (40–99 participants) were the most frequent, comprising 52% (*n* = 54) of all studies [47,72,85]. Large-scale trials (≥100 participants) accounted for 17% (*n* = 18), including Chuengsamarn et al. [34], *n* = 235, Chuengsamarn et al. [35], *n* = 213, and Yaikwawong et al. [118], *n* = 227. The increasing number of larger RCTs in recent years reflects enhanced methodological rigor and growing investment in clinical validation of *Curcuma longa*’s therapeutic potential. Together, these studies encompass a wide demographic and clinical spectrum that spans continents, age groups, and metabolic conditions, thereby demonstrating the versatility of *Curcuma longa* as a nutraceutical candidate. These characteristics demonstrate both the scientific maturity and translational potential of *Curcuma longa* as a dietary supplement for the prevention and management of metabolic syndrome and its complications, thereby ensuring the internal validity and generalizability of these findings across diverse populations.

### 3.5. Intervention Characteristics

The analysis of the included RCTs in the study revealed a consistent pattern that spanned across the intervention parameters, specifically concerning sample size, formulation type, and treatment duration.

#### 3.5.1. Sample Size Distribution

**Figure 7:**
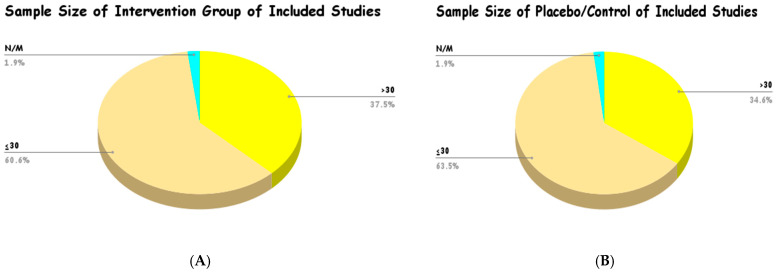
Sample size of intervention group (**A**) and placebo/control (**B**) of included studies.

Most of the studies that were included in this review had comparatively small sample sizes in the intervention and control groups. As depicted in Figure 7, the intervention group analysis showed that 60.6% of the included studies had a sample size of ≤30 participants, and 37.5% of the included studies had a sample size of >30 participants. Equally, in the placebo/control groups, 63.5% of the studies provided a sample size of ≤30, and 34.6% reported a sample size of >30. The minimal proportion of studies that did not state or calculate the sample size (N/M) was 1.9% in both groups. This trend points to the fact that much of the existing clinical evidence on *Curcuma longa* is based on smaller studies, and possibly single-center studies.

#### 3.5.2. Type of Formulations

**Figure 8:**
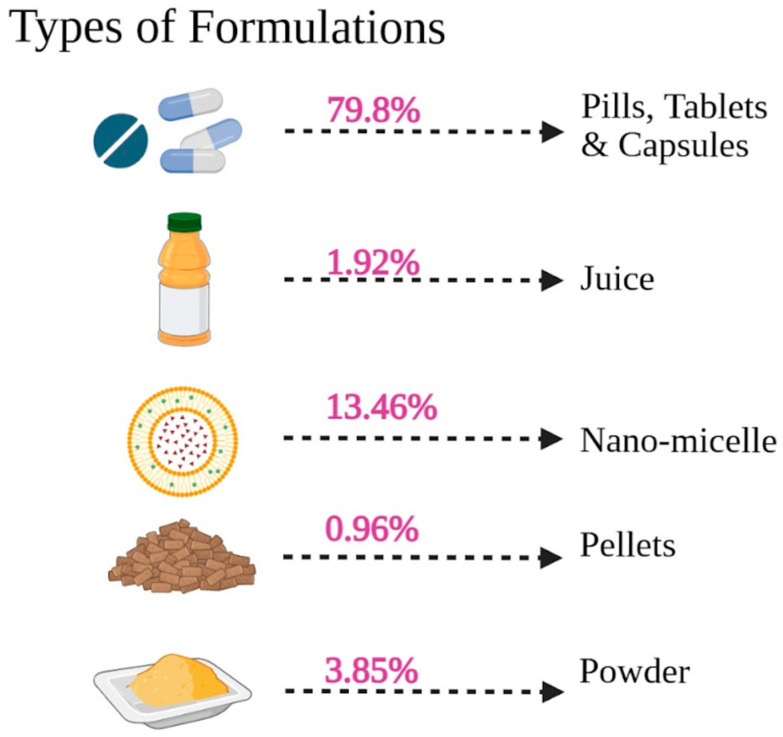
Types of formulations used in the selected studies.

The formulation of *Curcuma longa* used varied but was largely dominated by conventional oral dosage preparations, as observed in Figure 8 and Supplementary Table S3. The most frequently used modes of delivery were pills, tablets, and capsules, which represented 79.8% of the overall reported interventions. It is worth noting that a large percentage of the studies focused on new formulations aimed at improving bioavailability, with nano-micelle preparations comprising 13.46% of the total. Powder (3.85%), juice (1.92%), and pellets (0.96%) were also less frequently used. To give an example, many studies by Panahi et al. [85,86,87,88,89,90,91] involved capsules with 500 mg of curcuminoids and doses of 5 mg of piperine (Bioperile) in doses of one to two capsules per day. The other widespread approach was phospholipidated curcumin (e.g., Meriva or phytosomal preparations), which authors, including Mirhafez et al. [72,73,74,75] and Hariri et al. [47] used, in some instances providing 50 mg of pure curcumin per 250 mg or 500 mg. The upper limit of these increased forms, including two pills of 1000 mg Meriva that were studied by Hellmann et al. [49], points to the tendency to optimize absorption. These data indicate that most clinical evidence derives from defined supplemental forms of curcuminoids rather than habitual dietary turmeric consumption, and their results should be interpreted accordingly. It is apparent that the shift towards capsules/tablets and the specific delivery systems is an indication that the clinical literature is focused on optimizing the systemic absorption and efficacy of curcuminoids.

#### 3.5.3. Treatment Dosage

The review of the treatment protocols demonstrates a high degree of heterogeneity in the daily dosage and schedule of administration of *Curcuma longa* interventions, but the preference was for a middle-term period (Supplementary Table S3). The total daily dose of curcuminoids (or curcumin equivalent) was extremely variable, with a minimum of 80 mg/day and a maximum of 4000 mg/day. Nano-micelle (nano-curcumin) formulations that were created to ensure high bioavailability were most often used in the lowest therapeutic doses, and were usually used at a dose of 80 mg/day in single or divided doses (studies) [21,79,96]. On the other hand, the maximum dose was 4000 mg of curcumin per day, which was used in the short-term trial performed by Garg et al. [44]. The range of moderate doses was, however, agreed upon by most studies, often in the range of 500 mg/day [54] to 1000 mg/day [77,110] of regular or somewhat modified preparations. Bioavailability enhancers were typical, and most of the trials used 500 mg of curcuminoids in combination with 5 mg of piperine, which Panahi et al. studied in most trials [84,85,86,87,88,89,90,91], or phospholipidated curcumin (e.g., Meriva) at an equivalent dose of 50 mg of pure curcumin per day [72,73,74,75].

The dosing schedules were mostly categorized into two groups: once a day and twice a day. Many of the studies chose the convenience of once-daily administration [32,54,97], especially with ultra-low-dose nano-curcumin formulations. Nevertheless, the schedule of administration in two separate doses per day was also widespread, as it was commonly used to enhance tolerance or stable plasma concentrations. Examples of treatments studied with twice-a-day schedules included 500 mg capsules [58,84], 30 mg Theracurmin capsules [41], or augmented preparations, such as 1000 mg Meriva tablets [49]. Some of the studies that employed a thrice-a-day schedule included those by Yang et al. [120] and Heshmati et al. [51,52], or in one case, a 4-times-a-day schedule [76]. The general range of treatment ranged between 6 weeks and 12 weeks (3 months), which is the average intervention period of this systematic review.

#### 3.5.4. Treatment Duration

**Figure 9:**
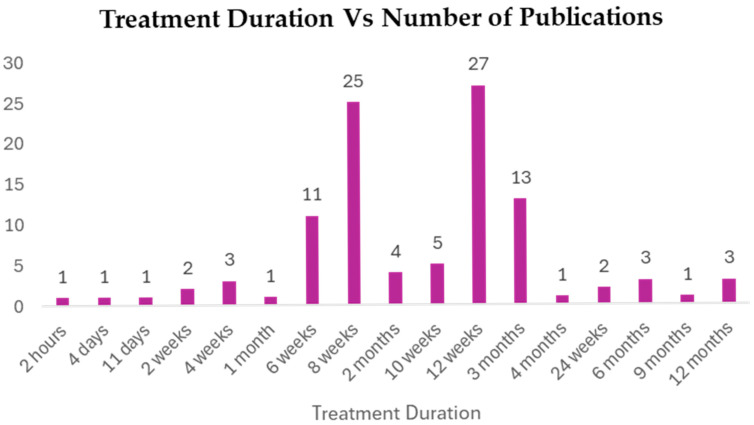
Treatment duration in reference to number of publications.

The *Curcuma longa* interventions were not homogeneous in terms of time, making it possible to observe both acute and short-term treatments (not longer than 2 h) as well as long-term interventions (up to 12 months) (Figure 9 and Supplementary Table S3). The data, however, were very much concentrated in short-to-medium-term regimens. The most commonly used treatment time was 12 weeks (mentioned in 27 publications), with an average of 8 weeks (25 publications). There were other similar durations of 3 months (13 publications) and 6 weeks (11 publications). This dispersion gives an indication that the majority of the trials measured the impact of *Curcuma longa* within two and three months, which is standard practice when it comes to evaluating the therapeutic efficacy of a treatment for a metabolic disorder. Long-term studies (e.g., 6, 9, and 12 months) on the impact of this treatment were significantly less prevalent, which may imply a gap in evidence on the long-term safety and efficacy of *Curcuma longa* after three months.

### 3.6. Clinical Efficacy Outcomes

#### 3.6.1. Metabolic Parameters

The clinical efficacy of *Curcuma longa* was measured across a wide spectrum of metabolic parameters that were systematically tested in the RCTs involved. This review is centered on the major indicators of the five parameters of Metabolic Syndrome (MetS): central obesity, dyslipidemia, hyperglycemia, and hypertension (Supplementary Table S4).

##### Anthropometric and Body Composition Indices

One of the central points of the research was the impact that *Curcuma longa* has on anthropometric measures that show the presence of central obesity. Body Mass Index (BMI), Waist Circumference (WC), and the Waist-to-Hip Ratio (W/H Ratio) were the major measures to be considered (Supplementary Table S5). The patient groups in the studies included mostly reported being overweight or mildly obese at baseline, with the means of the BMI values often around 30 kg/m^2^, such as in the intervention groups included in Saraf-Bank et al. [107] (31.0 ± 2.85 kg/m^2^), and Asadi et al. [24] (31.0 ± 14.1 kg/m^2^). In line with this, anthropometric measures of abdominal adiposity, including WC, were also regularly measured, with both baseline values being around 100 cm, as witnessed in the curcumin groups from Sangouni et al. [106] (100.2 ± 8.5 cm) and Asadi et al. [24] (101.1 ± 9.5 cm).

##### Glycemic Control and Insulin Resistance Markers

Body glucose homeostasis was evaluated with the indices of Fasting Blood Sugar (FBS), Hemoglobin A1c (HbA1c), Fasting Plasma Insulin (FPI) (Supplementary Table S6), and calculation of the Homeostasis Model Assessment of Insulin Resistance (HOMA-IR). Most of the evidence espoused the use of *Curcuma longa* in enhancing the glucose homeostasis mechanism and insulin sensitivity. There was a tendency for patients in the trials that were included to have signs of impaired glucose metabolism or diabetes at baseline. As an example, in several intervention groups, initial FBS levels were in the normoglycemic range of values, including in Asan et al. [25] (87.30 ± 8.20 mg/dL), which ranged to levels that were significantly higher, as seen in research such as that by Asadi et al. [24] (150.90 ± 58.10 mg/dL). Equally, long-term glycemic control as indicated by the HbA1c values showed significant heterogeneity, ranging between 5.95%, as indicated by Panahi et al. [87], and 8.18%, as indicated by Asadi et al. [24]. There was a high prevalence of markers of insulin resistance, including FPI [92] and HOMA-IR [100], which indicated the predisposition of the MetS population to being insulin-resistant. HbA1c is the most important long-term indicator, and was significantly reduced in several studies [26,34,80,118]. Similarly, the reduction in FBS in the trials by Asghari et al. [26], Chuengsamarn et al. [34], and Zohrabi et al. [121] was also significant; however, an increase in FPS was indicated in the trials by Khajehdehi et al. [64]. Moreover, HOMA-IR was repeatedly and considerably reduced in many studies, among them, Chuengsamarn et al. [34,35], Na et al. [80], Ismail et al. [54], Zohrabi et al. [121], Asghari et al. [26], and Yaikwawong et al. [118], which shows a strong influence on this pathogenic mechanism of MetS.

##### Lipid Profile Components

The lipid profiles of study participants were strictly measured by measuring Triglycerides (TG), Total Cholesterol (TC), LDL Cholesterol (LDL), and HDL Cholesterol (HDL) (Supplementary Table S7). A state of dyslipidemia, which is one of the essential elements of MetS, was verified with the help of the baseline lipid status of the participants. The intervention groups in most of the studies reported their baseline TG levels as exceeding 150 mg/dL The MetS diagnostic criteria include a range between 109.00 mg/dL [19] and 66.70 mg/dL [106]. The TC levels also were stable throughout the trials, and representative curcumin group baselines were 163 mg/dL [24] to 170.19 mg/dL [100]. Also, the level of HDL was usually low and was often below 40 mg/dL (e.g., [19]: 30 ± 2 mg/dL; [20]: 33.81 ± 8.14 mg/dL), highlighting the common lipid-related risk factors in the population. This benefit extended to the atherogenic lipoproteins, as LDL Cholesterol (LDL) also showed significant reductions in the same studies, with additional support from Rezaei M. (2024) [99]. Crucially, the beneficial HDL Cholesterol (HDL) level was reported to be significantly increased by Na et al. [80], Mansharifi et al. [70], and Pashine et al. [93], confirming a positive modulation of the dyslipidemia characteristic of Metabolic Syndrome.

##### Blood Pressure Indices

Finally, the effect of *Curcuma longa* on hypertension was evaluated by recording Systolic Blood Pressure (SBP) and Diastolic Blood Pressure (DBP) (Supplementary Table S7). Baseline SBP values were often in the hypertensive or pre-hypertensive range, such as the 142.07 ± 20.73 mmHg reported in the treatment group by Abed et al. [18]. Similarly, baseline DBP values generally indicated elevated pressure, with the curcumin groups in the studies by Abed et al. [18] and Sangouni et al. [106] reporting mean DBP values of 92.27 mmHg and 91.5 mmHg, respectively, prior to intervention. However, some studies did report a significant reduction in Diastolic Blood Pressure (DBP) [113], while results for Systolic Blood Pressure (SBP) were highly variable, with some studies reporting increases and others showing no change [64]. The overall effect of curcumin on blood pressure requires careful interpretation due to the inconsistencies observed, which suggest that the primary clinical benefit of *Curcuma longa* may be more pronounced in the glycemic and lipid profiles than in hypertension in this patient population.

#### 3.6.2. Inflammatory Parameters

The compelling evidence of the inflammatory parameters analyzed is a strong indication of the anti-inflammatory effects of *Curcuma longa* supplementation, which is a core of the mechanism of its effect on Metabolic Syndrome (MetS). The majority of the eligible Randomized Controlled Trials (RCTs) had a significant reduction in the most important inflammatory markers, which supports its contribution to the prevention of chronic low-grade inflammation, which is the pathology of MetS (Supplementary Table S8).

##### Acute-Phase Reactants and Pro-Inflammatory Cytokines

C-Reactive Protein (CRP), a widely utilized acute-phase reactant and strong predictor of cardiovascular risk, was the most consistently and significantly modulated inflammatory marker. Many studies have revealed that CRP reduced tremendously with an intervention with *Curcuma longa* (Supplementary Table S9). The major authors who have illustrated such a strong impact are Panahi et al. [85], Alizadeh et al. [21], Haroyan et al. [48], Adibian et al. [19], Afshar et al. [20], Asan et al. [25], Javandoosi et al. [58], Majeed et al. [69], and Mamsharifi et al. [70]. Although other studies by Kocher et al. [67] and Garg et al. [44] did not show a change, the overall results showed a positive effect of anti-inflammatories as indicated by a reduction in CRP.

The effects on the Interleukin family were more or less to stifle pro-inflammatory species. Interleukin-6 (IL-6) and insulin resistance major mediators were significantly reduced in numerous publications, such as those by Alvarenga et al. [23], Asghari et al. [26], Boshagh et al. [31], Kisiolek et al. [66], Kocher et al. [67], Mamsharifi et al. [70], and Panahi et al. [88], and Interleukin-8 (IL-8) was also reported to be reduced by Khajehdehi et al. Anti-inflammatory cytokine Interleukin-10 (IL-10), on the other hand, had been demonstrated to rise in certain studies like Mamsharifi et al. [70] and indicated a change towards the anti-inflammatory state(Supplementary Table S9).

Tumor Necrosis Factor-alpha (TNF-a), which is an important upstream pro-inflammatory cytokine, was also significantly and consistently decreased in various trials, which supports the mechanism of curcuminoids as an anti-inflammatory. Alizadeh et al. [21], Alvarenga et al. [23], Asan et al. [25], Jazayeri-Tehrani et al. [59], and Panahi et al. [87,88] reported this effect of Curcuma longa, which inhibits systemic inflammation at a critical regulatory point (Supplementary Table S10).

##### Adipokines and Other Inflammatory Mediators

The action of *Curcuma longa* is not limited to conventional metabolic markers but also impacts the regulation of adipokine and non-cytokine inflammatory mediators, with emphasis on its multi-targeted action in the treatment of the chronic, low-grade inflammation of Metabolic Syndrome (MetS). Important chemokine recruitment of macrophages to adipose tissue, leading to insulin resistance, was reported to be markedly decreased after supplementation in the studies by Panahi et al. [87] and Sedighiyan et al. [109] using Monocyte Chemoattractant Protein-1 (MCP-1). Also, there was a positive regulation of adipocyte-derived signaling molecules (adipokines) directly associated with insulin resistance: Visfatin and Resistin were highly reduced by Ismail et al. [54] and Sedighiyan et al. [109]. Lastly, *Curcuma longa* was shown to inhibit other essential inflammation pathways, suggesting a substantial reduction in NLRP3 [54], a central constituent of the inflammasome, and Fetuin-A (Fe-A) [109], an inflammatory glycoprotein associated with insulin resistance and ectopic fat deposition (Supplementary Table S11). All these findings validate that *Curcuma longa* has a multi-layered anti-inflammatory and immunomodulatory effect, which is fundamental in the management of MetS therapeutically.

#### 3.6.3. Oxidative Parameters

The measurement of the parameters of oxidative stress gives a very important mechanistic interrelation between the clinical efficacy of *Curcuma longa* and the pathophysiology of Metabolic Syndrome (MetS). Oxidative stress, which is an imbalance between the generation of reactive oxygen species (ROS) and antioxidant mechanisms in the body, is strongly involved in all aspects of MetS. The results of the reviewed RCTs, as a rule, support the presence of a significant antioxidant effect, which is manifested in a decrease in the indicators of oxidative damage and an enhancement in endogenous antioxidant potential (Supplementary Table S12).

##### Markers of Oxidative Damage

One of the major indicators of oxidative damage is the amount of lipid peroxidation products, which was also consistently and positively altered by *Curcuma longa* supplementation. Thiobarbituric acid-reactive substances (TBARS) and Malondialdehyde (MDA) constitute important end products of lipid peroxidation and are well-known biomarkers of oxidative stress. Most of the available studies reported a significant reduction in MDA (Supplementary Table S13), including Alizadeh et al. [21], Darmian et al. [37], Jarhahzaden et al. [57], Osali et al. [83], Panahi et al. [85,86,87,88,89], and Saraf-Bank et al. [107,108]. Some trial studies reported a significant reduction in TBARS (Supplementary Table S14), which were significantly reduced in the trial by Krishnareddy et al. [68]. This repeated decrease points out the capacity of *Curcuma longa* to shield the cellular membranes against oxidative attack. The balance between prooxidants/antioxidants was also checked. Alvarenga et al. [23] found a significant reduction in the prooxidant antioxidant balance (PAB) of the trial, which is an indication of a desirable change in favor of a non-prooxidative state. On the other hand, a single study by Jimenez-Osorio et al. [60] demonstrated that MDA increased, and this observation might have been due to heterogeneity in formulation or patient population.

##### Endogenous Antioxidant Defenses

The therapeutic action of *Curcuma longa* is further involved in strengthening the body’s defense mechanism, which is demonstrated by a change in major antioxidant enzymes and molecules. Important enzymatic antioxidants such as Superoxide Dismutase (SOD) and Glutathione Peroxidase (GPx) were often found to have been significantly raised after intervention (Supplementary Table S13). The studies by Panahi et al. [85,86,87,88,89], Krishnareddy et al. [68], and Heshmati et al. [51] reported an increase in SOD, while GPx was increased in the studies by Krishnareddy et al. [68] and Heshmati et al. [52]. This suggests that bioactive curcuminoids actively upregulate the endogenous enzymatic scavenger system. Glutathione (GSH), the master non-enzymatic antioxidant, was also reported to be significantly increased by Panahi et al. [85] and Krishnareddy et al. [68]. Concurrently, its oxidized form, Glutathione Disulfide (GSSG), was found to be significantly decreased by Jimenez-Osorio et al. [60], as was Glutathione Reductase (GR). This pattern collectively indicates a shift towards a more reduced, and therefore more protective, cellular environment. Measures of the plasma’s overall non-enzymatic antioxidant capability, TAC and TAS, were consistently and significantly increased across multiple trials, including those by Panahi et al. [88], Alizadeh et al. [21], Saraf-Bank et al. [107], and Mokhtari et al. [79] (Supplementary Table S14). This provides compelling evidence that *Curcuma longa* enhances the systemic antioxidative buffering capacity. In summary, the highly consistent findings of reduced oxidative damage (MDA/TBARS) and augmented anti-oxidant defenses (SOD, GPx, GSH, and TAC/TAS) strongly establish the potent antioxidative mechanism of Curcuma longa, positioning it as a nutraceutical capable of addressing the core component of cellular stress in MetS.

### 3.7. Meta-Analysis of RCTs Regarding the Clinical Potential of Curcuma longa Linn. as a Nutraceutical for Metabolic Syndrome

We pooled randomized controlled trial data to evaluate the effect of *Curcuma longa* (curcumin) supplementation on anthropometric measures, glycemic control, blood pressure, lipid profile, insulin resistance and secretion indices, inflammatory markers, and oxidative stress biomarkers across clinical populations relevant to metabolic syndrome (Type 2 diabetes mellitus [T2DM], metabolic syndrome [MetS], non-alcoholic fatty liver disease [NAFLD], obesity, and polycystic ovary syndrome [PCOS]) (Supplementary Figures S1–S19). Analyses used random-effects models and are reported as standardized mean differences (SMD) with 95% confidence intervals (CI). Heterogeneity is described with tau^2^ and I^2^; *p*-values refer to two-sided tests.

#### 3.7.1. Anthropometry: BMI, Waist Circumference (WC), Waist-to-Hip Ratio (WHR)

Across disease-specific subgroups, curcumin supplementation produced notable improvements in body composition, particularly among patients with type 2 diabetes mellitus (T2DM) and obesity (Supplementary Figures S1–S3). Across clinical subgroups, the pooled effects on BMI were inconsistent. In diabetes cohorts (12 studies), the pooled SMD was −0.27 (95% CI −0.57 to 0.02), with a non-significant trend toward lower BMI with curcumin; substantial heterogeneity was present (I^2^ ≈ 79%, *p* < 0.01), suggesting results varied across trials. For metabolic syndrome cohorts (7 studies, 195 vs. 195 subjects), the pooled SMD was 0.09 (95% CI −0.23 to 0.41), i.e., no effect and moderate heterogeneity (I^2^ ≈ 58%, *p* = 0.03). NAFLD and overweight/obese sub-groups likewise showed no significant pooled BMI effects. Overall, the evidence does not support a consistent clinically meaningful BMI reduction from curcumin; where an effect was suggested (small negative SMD in some diabetes/obese analyses), heterogeneity limited confidence. The wide 95% prediction interval further suggests that while some future studies might detect meaningful BMI reductions, others could show negligible effects. Trials by Panahi et al. [89,90] demonstrated relatively strong reductions, whereas others (e.g., Asadi et al. [24]; Yakawong et al. [119]) reported minimal change.

Findings for WC were mostly null. A pooled SMD of −0.33 (95% CI −0.81 to 0.15), which was non-significant with high heterogeneity (I^2^ ~ 75%, *p* < 0.01), was found in diabetes patients (5 studies; 292 vs. 293 subjects). The same was also observed with obese/overweight populations and metabolic syndrome populations, which did not exhibit any consistent WC benefit. This was thus viewed as a non-significant, inaccurate effect that had low between-study significance, since there was no strong evidence to support the hypothesis that curcumin lowers central adiposity. The pooled SMDs across the NAFLD, overweight/obese, and metabolic syndrome subgroups were small and nonsignificant (e.g., NAFLD SMD 0.11, 95% CI 0.37 to 0.15, and overweight SMD 0.47, 95% CI 0.10 to 1.03, respectively, with moderate heterogeneity in the latter). In sum, there was no significant influence on WHR.

#### 3.7.2. Blood Pressure: SBP and DBP

During the analysis of SBP, for diabetes patients (7 studies; 188 vs. 176), pooled SMD was −0.11 (95% CI −0.45 to 0.23), which is not significant; heterogeneity was moderate (I^2^ ≈ 61%, *p* = 0.02). A statistically significant difference was found in patients with metabolic syndrome (4 studies; 105 vs. 104), pooled SMD was −0.65 (95% CI −1.21 to −0.08), *p* = 0.05, but the heterogeneity was observed to be high (I^2^ ≈ 65, *p* = 0.03). There were no significant changes in SBP in NAFLD and the overweight/obese subgroups. Accordingly, curcumin could potentially be used to lower SBP in specific trials on metabolic syndrome, but the heterogeneity and the small number of studies warn against generalizing these findings (Supplementary Figure S4). In diabetes cohorts, pooled DBP SMD was −0.34 (95% CI −1.18 to 0.49), which was non-significant, but had high heterogeneity (I^2^ ≈ 86%, *p* < 0.01) in both cases of DBP. In patients with metabolic syndrome (3 studies; 94 vs. 93), pooled SMD was −0.40 (95% CI −0.79 to −0.01), *p* < 0.05, indicating a slight DBP decrease in that group with low inter-study variability reported. No consistent effect of DBP was demonstrated in other sub-groups. Generally, small, positive BP changes seem to be most reproducible in trials involving metabolic syndrome. (Supplementary Figure S5).

#### 3.7.3. Glycemic Control: Fasting Blood Sugar (FBS), Blood Glucose, HbA1c

As depicted in Supplementary Figures S6–S8, fasting blood sugar (FBS) results differed by population. In diabetes patients (9 studies; 323 vs. 320) pooled SMD was −0.61 (95% CI −1.48 to 0.25), which was not statistically significant and very highly heterogeneous (I^2^ ≈ 88%, *p* < 0.01). By contrast, in metabolic syndrome cohorts (6 studies; 197 vs. 198), pooled SMD was −0.25 (95% CI −0.48 to −0.03), *p* < 0.05, indicating a small but statistically significant reduction in FBS with curcumin and low heterogeneity reported. Obese/overweight and NAFLD subgroups were generally null. The pattern suggests curcumin may modestly improve fasting glycemia in metabolic syndrome populations, while effects in established diabetes are heterogeneous and inconclusive.

In blood glucose (post-prandial/other glucose measures) measured in diabetes patients (4 studies; 241 vs. 237), an SMD of −0.53 (95% CI −0.82 to −0.23), *p* < 0.05, was published with medium effects (I^2^ ≈ 58%, *p*≈0.07). This means that measured blood glucose showed a moderate reduction in trials measuring such endpoints. The possible small-study effects of this outcome were proposed by funnel/Egger’s tests. In diabetes cohort designs (10 studies; 505 vs. 504) that reported HbA1c, SMD was −0.33 (95% CI −0.58 to −0.09), *p* < 0.05, with moderate heterogeneity (I^2^ ≈ 66%, *p* < 0.01). This is a minor yet statistically significant increase in long-term glycemic control. The effect of HbA1c was not significant and extremely heterogeneous in trials of metabolic syndrome. On the whole, HbA1c and some of the glucose measures indicate consistent minor changes in the cohorts with diabetes, but inter-study variability dampens confidence.

#### 3.7.4. Insulin Resistance and Beta-Cell Indices: HOMA-IR, HOMA-B, QUICKI, Serum Insulin

Across all clinical populations, curcumin supplementation showed no significant improvement in insulin resistance indices (Supplementary Figures S9–S12). Results for HOMA-IR were largely null across populations. In diabetes (9 studies; 537 vs. 535), pooled SMD was −0.01 (95% CI −0.78 to 0.76), which was non-significant, with extremely high heterogeneity (I^2^ ≈ 95%, *p* < 0.01). The subgroups of overweight/obese, PCOS, and NAFLD patients also did not exhibit a reliable pooled benefit. Overweight/obese, PCOS, and NAFLD subgroups likewise showed no reliable pooled benefit. This is indicative that pooled trials do not demonstrate consistent improvements in HOMA-IR that are attributable to curcumin. As for HOMA-B (beta-cell function), in diabetes cohorts (3 studies; 104 vs. 101), pooled SMD was 0.09 (95% CI −0.19 to 0.36), which was non-significant and without notable heterogeneity. There is thus no evidence of a consistent beneficial effect on beta-cell secretion indices. QUICKI across NAFLD (3 studies) and PCOS (4 studies) cohorts revealed SMDs that were non-significant (e.g., NAFLD-QUICKI SMD 0.41, 95% CI −0.39 to 1.21 with very high heterogeneity). No robust signal favoring curcumin was discovered. Diabetes cohorts for serum insulin (5 studies; 154 vs. 151) revealed a pooled SMD of −0.33 (95% CI −0.77 to 0.11), which was non-significant with high heterogeneity (I^2^ ≈ 75%, *p* < 0.01). Overweight/obese and PCOS cohorts were likewise non-significant. Taken together, there is no conclusive benefit to fasting insulin or direct insulin-sensitivity indices.

#### 3.7.5. Lipids: TC, LDL-C, HDL-C, TG

Curcumin supplementation produced notable improvements in serum lipid parameters across several metabolic conditions (Supplementary Figures S13–S16). In measurements of total cholesterol (TC) across diabetes, overweight/obese, PCOS, metabolic syndrome, and NAFLD subgroups, most pooled SMDs were small and non-significant (e.g., diabetes SMD −0.22, 95% CI −0.45 to 0.01). Heterogeneity varied by subgroup (some high). There was no observed consistency in the capacity of curcumin to lower TC according to the analysis. In the case of LDL-C, in diabetes cohorts (10 studies; 387 vs. 388) pooled SMD was −0.19 (95% CI −0.45 to 0.08), which was non-significant with moderate–high heterogeneity (I^2^ ≈ 69%, *p* < 0.01). The overweight/obese subgroup showed a statistically significant LDL reduction (9 studies; pooled SMD −0.36, 95% CI −0.64 to −0.07, *p* < 0.05), though with moderate heterogeneity (I^2^ ≈ 56%, *p* = 0.02). Some evidence of small-study/publication bias for LDL in diabetes analyses was flagged by Egger’s test. The outcomes for HDL-C were more favorable. For instance, diabetes cohorts showed a pooled SMD of 0.40 (95% CI 0.03 to 0.77), *p* < 0.05 (10 studies; 285 vs. 284), though heterogeneity was substantial (I^2^ ≈ 72%, *p* < 0.01). Overweight/obese cohorts also showed a smaller positive HDL effect (SMD 0.27, 95% CI 0.05 to 0.49). These suggest curcumin supplementation may modestly increase HDL in some metabolic populations, but between-study inconsistency reduces certainty. There were mixed findings for triglycerides (TG). For example, the diabetes cohort displayed a pooled TG SMD of −0.29 (95% CI −0.64 to 0.07), which was non-significant with high heterogeneity. In the NAFLD and some overweight/obese cohorts, analyses showed modest TG reductions (e.g., NAFLD pooled SMD −0.27, 95% CI −0.49 to −0.05, *p* < 0.05), suggesting context-specific improvements. Overall lipid effects appear modest: some benefit were found for HDL and certain subgroup improvements for LDL and TG but this was inconsistent across populations.

#### 3.7.6. Inflammation: CRP and TNF-Alpha

Across metabolically compromised populations, curcumin substantially reduced CRP (Supplementary Figure S17). CRP pooled analyses frequently favored curcumin with small-to-moderate effects. For diabetes patients (5 studies; 195 vs. 197) pooled SMD was −0.39 (95% CI −0.64 to −0.14), *p* < 0.05, which is a statistically significant reduction with little between-study variability reported. In obese/overweight patients (3 studies; 98 vs. 98) pooled SMD was −0.40 (95% CI −0.68 to −0.11), *p* < 0.05, again with low heterogeneity. These consistent CRP reductions across several populations indicate curcumin exerts an anti-inflammatory effect that is measurable through lower CRP. The funnel/Egger’s tests did not indicate publication bias for these CRP pools. TNF-α pooled effects were larger but heterogeneous. In diabetes cohorts (3 studies; 246 vs. 248) pooled SMD was −1.07 (95% CI −2.05 to −0.09), *p* < 0.05, suggesting a large point estimate reduction; however, heterogeneity was extreme (I^2^ ≈ 91%, *p* < 0.01), and the Egger’s test suggested funnel asymmetry, raising concern for bias and instability in the pooled estimate (Supplementary Figure S18). While some trials report sizable TNF-α reductions, this supports a biologically plausible anti-inflammatory mechanism that is relevant to insulin resistance and tissue injury.

#### 3.7.7. Oxidative Stress: TAC, GSH, MDA

As depicted in Supplementary Figure S19, Total Antioxidant Capacity (TAC) for diabetes cohorts (5 studies; 114 vs. 107) revealed a pooled SMD of 0.68 (95% CI −0.30 to 1.67), which is not statistically significant; heterogeneity was high (I^2^ ≈ 85%, *p* < 0.01). This wide, imprecise estimate indicates that some trials saw increased TAC with curcumin, but the effects were inconsistent. For Malondialdehyde (MDA), a pooled diabetes analysis (5 studies) yielded an SMD of −0.22 (95% CI −0.54 to 0.09), which is non-significant with low heterogeneity. This suggests little consistent effect on this lipid-peroxidation marker across trials. In the case of Glutathione (GSH). A pooled (3 studies; 61 vs. 59) SMD of 0.91 (95% CI −0.88 to 2.69) was found, which is non-significant, and extreme heterogeneity (I^2^ ≈ 92%, *p* < 0.01) was observed. Sparse data and large between-study differences prevent the formation of reliable conclusions for GSH. Overall, oxidative biomarkers show heterogeneous and imprecise effects; although some trials report antioxidant improvements, the pooled evidence is inconsistent.

Across the pooled randomized trials, curcumin/*Curcuma longa* supplementation showed consistent small anti-inflammatory effects (notably reductions in CRP) and modest improvements in some glucose metrics (HbA1c, certain glucose measures) and HDL in selected populations. Signals for SBP/DBP reductions are present primarily in metabolic syndrome subgroups. Effects on anthropometry, insulin-resistance indices (HOMA-IR, QUICKI), LDL/TC, and many oxidative markers are inconsistent when pooled; many analyses are limited by substantial between-study heterogeneity (I^2^ frequently >50–75%).

#### 3.7.8. Pooled Effects and Quality of Evidence for Curcumin Supplementation

The pooled evidence (Table 3 and Table 4) suggests that curcumin offers meaningful but selective benefits across metabolic and inflammatory pathways. Glycemic control and inflammation are the most steadily improved. Glucose in the blood after eating, HbA1c, and fasting glucose were all reduced without much difference between short-term and long-term effects. These findings are consistent with a moderate GRADE certainty, despite a certain degree of inconsistency in the trials. Blood pressure was also somewhat the same; a decrease in systolic and diastolic blood pressure, particularly in the case of individuals with metabolic syndrome, is an indication of a clinically significant cardiovascular impact.

The effects on lipids were more ambivalent. The change in total cholesterol was minimal and not significant, but LDL-C decreased slightly, and HDL-C increased considerably. Triglycerides were reduced, particularly in NAFLD research. There was moderate certainty with regard to TG and HDL-C, but certainty with regard to TC and LDL-C was undermined by heterogeneity and small-study bias. In general, there is a small lipid-modifying effect, which, however, is subject to population and formulation.

The anthropometric findings were a different story. There was no significant change in BMI, waist circumference, or waist–hip ratio. The null findings are probably due to the high heterogeneity and short intervention times of the identified studies. The insulin measures responded similarly; HOMA-IR, insulin, QUICKI, and HOMA-B did not respond in a consistent manner. The extremely large I^2^ values illustrate the inconsistencies in the study designs and formulations, with low to very low GRADE certainty.

The most evident benefits were demonstrated by inflammatory markers. CRP decreased steadily, and heterogeneity was low; TNF-alpha changed, and the trend remained positive. This aligns with the profile of the anti-inflammatory properties of curcumin, which help to promote its metabolic action. On the contrary, oxidative stress indicators (TAC, GSH, MDA) provided inconclusive, primarily non-significant results. Low certainty was caused by low sample sizes, inconsistencies in assays, and broad confidence intervals.

## 4. Discussion

This comprehensive systematic review and meta-analysis of 104 randomized controlled trials (RCTs) provides the most extensive synthesis to date of the clinical effects of *Curcuma longa* supplementation on metabolic syndrome (MetS) and related cardiometabolic conditions, including type 2 diabetes mellitus (T2DM), non-alcoholic fatty liver disease (NAFLD), obesity, and polycystic ovary syndrome (PCOS). Although the metabolic and anti-inflammatory properties of *Curcuma longa* and its principal bioactive constituent, curcumin, have been extensively described in preclinical and clinical literature, the present systematic review and meta-analysis provide important comparative and quantitative insights that extend beyond prior narrative syntheses. Rather than demonstrating uniform benefits across all components of metabolic syndrome, our findings indicate a pattern of selective efficacy, with the most consistent and clinically relevant effects observed in inflammatory modulation and glycemic control, and more variable or context-dependent effects on anthropometric and insulin-resistance indices.

The results of the glycemic control were subtle. Although the impacts on fasting blood glucose were mixed, especially in already-diabetic patients, HbA1c showed significant, yet statistically insignificant, improvements in cohorts with diabetes, suggesting that curcumin might have a cumulative effect on long-term glycemic control, but not on acute glucose metabolism. This difference has the clinical implication of making HbA1c a relatively robust outcome measure in nutraceutical trials, as the subtle effects of metabolic changes may not be detected in the short run. These improvements, though small, are clinically significant: even minor improvements in HbA1c can have significant results on reductions in microvascular and macrovascular outcomes in the long term [122,123]. The same selectivity pattern was shown in lipid-profile modulation. The changes in HDL cholesterol and triglycerides were more favorable in the case of overweight/obese and non-alcoholic fatty liver disease populations, and the total cholesterol and LDL cholesterol were different across the disease categories. These observations imply that the lipid-modifying effect of curcumin is a secondary effect of its anti-inflammatory and antioxidant effects, but not a sign of a lipid-lowering effect comparable to pharmacological compounds. All these findings indicate that curcumin also acts on lipid transport and hepatic lipid metabolism, which are some of the characteristics of metabolic dysfunction.

Anthropometric changes followed a subtler pattern. Anthropometric outcomes like body mass index, waist circumference, and waist-to-hip ratio did not show statistically reliable pooled benefits. This is not a coincidence in some individual trials, but it is probable that it is due to a combination of biological and methodological issues. There is no evidence that curcumin is a major weight-reduction agent, but its metabolic effects can be realized without significant adiposity changes in response to the short-term interventions (6–12 weeks) that prevail in the literature. As central obesity is more prognostically meaningful than total weight, these findings may reflect curcumin’s influence on adipose inflammation rather than generalized weight loss [124,125]. Additional comparative subgroup analyses indicate that the trials that reported significant changes in anthropometry with a modest degree of change were also more likely to have a higher baseline of inflammation or longer periods of intervention, which points to the significance of population traits and study design in the interpretation of such results. Inflammatory markers demonstrated some of the most consistent responses. C-reactive protein (CRP) decreased markedly across multiple trials, supported by reductions in TNF-α, though with significant heterogeneity and some evidence of publication bias for the latter. Given the central role of low-grade inflammation in the pathophysiology of MetS, these anti-inflammatory actions provide mechanistic coherence to the improvements observed in glycemic and lipid variables. Oxidative stress outcomes were also responsive but heterogeneous. Multiple individual RCTs documented reductions in malondialdehyde (MDA) alongside increases in antioxidant enzymes such as SOD, GPx, and GSH, with several studies demonstrating an enhancement in TAC/TAS levels as evidence of systemic antioxidant reinforcement. However, substantial variability in assays, units, and reporting prevented consistent pooled effects. Thus, while mechanistically plausible, oxidative stress remains the least consistent evidence domain. Taken together, the evidence positions curcumin as a broad-spectrum nutraceutical component capable of targeting multiple interconnected metabolic pathways, reflecting the complex multimorbidity embedded in MetS.

These clinical results align closely with the known molecular mechanisms of curcumin. Preclinical evidence supports curcumin’s ability to modulate inflammatory signaling through the inhibition of NF-κB activation, downregulation of iNOS and COX-2, and attenuation of pro-inflammatory cytokines, including TNF-α and IL-6. Curcumin also activates AMPK, promoting glucose uptake in skeletal muscle, inhibiting hepatic gluconeogenesis, and enhancing fatty acid oxidation, which are mechanistic actions consistent with improvements in fasting glucose, HbA1c, triglycerides, and central adiposity [126,127,128]. The antioxidant effects of curcumin, such as the improvement of endogenous enzymatic defenses and the decrease in lipid peroxidation, provide further evidence on the use of curcumin to induce reductions in oxidative stress as a central factor in insulin resistance and endothelial dysfunction. Moreover, new human data show that curcumin regulates the intestinal microbiota, reinforces the intestinal barrier, and affects the intestinal permeability due to the interactions between the microbiome–immune–metabolic axis [11,129]. These gut-based processes can be the reason why metabolic effects, which are of clinical significance, can be implemented even in cases when plasma curcumin levels are low. On the whole, the combination of mechanistic plausibility and clinical observations contributes to the increased confidence in curcumin’s therapeutic potential.

Among the most impressive pieces of evidence that this review has given is the extent to which the effects of curcumin are formulation dependent. Almost 80% of the included trials involved the use of capsule/tablet products, though bioavailability-enhancing strategies (including piperile, phospholipid complexes, e.g., Meriva) and nano-micellar had a high degree of variation. Approximately 13.5% of the studies included in the scope of the study were nano-formulations, and phospholipidated curcumin and piperine-enhanced extracts also formed significant proportions of the study. Clinical trials with enhanced-bioavailability preparations often found greater and more sustained metabolic activities compared to the traditional extracts [130,131]. Dosage was also quite wide, ranging between 80 mg/day and 4000 mg/day, with nano-formulated curcumin demonstrating results at 80 mg/day and above; conventional preparations have generally had to use a dosage of 500–1500 mg/day before they could show any effects. This supports the claim that the therapeutic potential of curcumin is dependent not on nominal dose but on the pharmacokinetics of curcumin. The main range of treatment was 8–12 weeks, with comparatively low numbers of studies beyond 6 months, restricting the findings on durability and long-term safety. Metabolic remodeling can usually come at a cost, and therefore future RCTs need to prolong the period of intervention in order to understand whether temporary biomarker improvements are converted to long term risk reduction.

This review is distinguished by its breadth (104 RCTs), rigorous stratification by disease subtype and formulation, thorough risk-of-bias appraisals, and the use of the GRADE framework to determine some degree of certainty across outcomes. These features are the key features of this review. The integration of anthropometric, glycemic, lipid, inflammatory, and oxidative stress indicators in a single metabolic model can be seen as providing a comprehensive view of the therapeutic profile of curcumin and a source of clarity in the face of inconsistent results in the past. Importantly, because ~80% of trials used concentrated oral supplements (capsules/tablets/advanced delivery systems) and only a few trials tested turmeric in food-style preparations, our findings primarily reflect the efficacy of defined curcumin products and formulations rather than the effects of culinary turmeric as consumed in habitual diets. Bioavailability enhancers (piperine, phytosomes, nano-formats) were associated with larger and more consistent biomarker changes; this pharmacokinetic dependence reduces the external validity of extrapolating our pooled estimates to regular dietary turmeric intake. Future RCTs should evaluate food-based interventions (standardized powder/food matrices) and head-to-head comparisons with supplement formulations to determine the translational relevance of these findings to diet and public health recommendations.

## 5. Limitations

Although the findings are promising, the heterogeneity within the studies is still one of the key weaknesses. The moderate–high I^2^s in various pooled analyses indicate the variations in population, curcumin formulations, disease severity, and quality of studies. Even though the Cochrane RoB 2 evaluations showed that the risks of bias were generally low in the domains of blinding and attrition, issues were still present in the randomization procedures, nonadherence to planned interventions, and missing outcome reporting in some studies. The geographical concentration of trials, especially the fact that most of the trials were based in Iran and other Middle Eastern countries, raises other questions in terms of external validity, because 67% of all the included trials were based in the Middle East, with scanty representation in North America, Europe, and other regions in Asia. Regional nutritional backgrounds, genetic distributions, local diets, and local clinical practices can vary widely, affecting the metabolic reactions to nutritional interventions like curcumin. Sample sizes were also mainly small, with about 60% of studies involving ≤30 subjects per arm, which is a source of imprecision risks and small-study effects. These are the methodological shortcomings that point to the necessity of larger, multisite, and internationally diverse RCTs.

## 6. Recommendation for Future Trials

The synthesis outlines a number of research priorities that are needed in order to advance the potential of curcumin in translational research: Large, multi-center RCTs using standardized formulations and longer follow-up durations to assess long-term clinical effects and the sustainability of biomarker changes; head-to-head comparisons of traditional, piperine-enhanced, nano-formulated, and phospholipidated curcumin to explain exposure–response relationships; the interactions of curcumin with the gut microbiota, systemic absorption, and tissue distribution to determine the mechanisms by which curcumin has metabolic effects in spite of its low plasma bioavailability; consistency in reporting safety outcomes, especially hepatic and gastrointestinal outcomes, drug–nutrient interactions, and long-term tolerability in vulnerable populations; stratified analyses of metabolic phenotypes, such as sex-specific responses and differences depending upon baseline inflammation or insulin resistance.

## 7. Conclusions

The findings endorse curcumin as an adjunctive treatment for those with MetS, T2DM, NAFLD, or obesity. The pooled evidence from RCTs indicates that curcumin supplementation yields consistent anti-inflammatory effects and modest yet clinically meaningful improvements in glycemic regulation and selected lipid parameters in individuals with metabolic syndrome and related disorders. These benefits are most pronounced with bioavailability-enhanced curcumin formulations, underscoring the importance of formulation in determining therapeutic efficacy. Importantly, curcumin should be viewed as a nutraceutical adjunct to standard care rather than a stand-alone therapeutic alternative, with potential utility in mitigating metabolic risk and low-grade systemic inflammation. Curcumin has a multi-target profile, so it can be especially useful in preventing early metabolic pathology before the disease is incurable and requires intensive pharmacological treatment.

## Figures and Tables

**Table 1:** Eligibility criteria for the selected studies.

Component	Description
Population (P)	Healthy people at risk of developing metabolic syndrome, pre-diabetic patients, diabetic patients, CVD patients, and patients with one of the conditions of metabolic syndrome.
Intervention (I)	Supplementation with *Curcuma longa* Linn. (turmeric) or curcumin extracts, including enhanced bioavailability formulations (e.g., nanocurcumin, curcumin-piperine). Administered as capsules, tablets, powders, or nutraceutical formulations.
Comparator (C)	Placebo, no treatment, or standard care/control diet. RCTs with active comparators (e.g., another supplement) will be included only if curcumin-specific outcomes can be extracted.
Outcomes (O)	Primary outcomes: Core components of MetS such as fasting blood glucose, HbA1c, triglycerides, total cholesterol, HDL-C, LDL-C, systolic and diastolic blood pressure, and waist circumference. Secondary outcomes: Inflammatory markers (CRP, TNF-α, IL-6), oxidative stress markers (MDA, TAC), insulin resistance indices (HOMA-IR), liver enzymes, and body mass index (BMI).

**Table 2:** General characteristics of included studies.

Author	Year	Country	Population	Gender	Age				Sample Size	
					Intervention		Control			
					Mean	SD	Mean	SD	Intervention	Control
Abed et al. [18]	2021	Iraq	Post-ischemic stroke patients	M, F	51.37	9.35	50.95	10.03	24	18
Adibian et al. [19]	2019	Iran	Type 2 Diabetes patients	M, F	58	NM	60	7	21	23
Afshar et al. [20]	2020	Iran	Hemodialysis patients	M, F	55.33	12.95	59.05	7.68	27	27
Alizadeh et al. [21]	2017	Iran	Infertile men	Male	30.54	4.03	30	3.96	28	28
Alvarenga et al. [22]	2020	Brazil	Hemodialysis Patients	M, F	54	15	53	12	14	14
Alvarenga et al. [23]	2022	Brazil	Patients undergoing hemodialysis	M, F	54	15	53	12	14	14
Asadi et al. [24]	2019	Iran	Type-2 diabetic patients	M, F	53.3	6.5	54.6	6.2	40	40
Asan et al. [25]	2020	Turkey	Women with polycystic ovary syndrome	F	27.6	3.6	28.3	5.9	15	15
Asghari et al. [26]	2024	USA	Type 2 Diabetes Mellitus patients (Eicosapentaenoic acid EPA group) Curcumin group Curcumin + EPA group	M, F	56.88 54.56 56.68	8.36 8.3 10.25	57.48	11.27	25	25
Askari et al. [27]	2022	Iran	COVID-19 outpatient	M, F	43.74	12.9	51.52	13.8	23	23
Atakan et al. [28]	2022	Turkey	Overweight and obese women (hyperlipidemia)	F	25–65				35	35
Barber-Chamoux et al. [29]	2017	France	Healthy Smokers	M, F	56	4.1	56	4.1	9	9
Bateni et al. [30]	2021	Iran	Patients with metabolic syndrome	M. F	50	9	54	7	22	21
Boshagh et al. [31]	2023	Iran	Patients with ischemic stroke	M, F	59.48	5.15	60.12	3.12	27	29
Campbell et al. [32]	2019	USA	Young obese men	M	18–35 (range)				11	11
Chashmniam et al. [33]	2019	Iran	Non-Alcoholic Fatty Liver Disease Patient	M, F	46.56	11.25	37.75	14.4	25	20
Chuengsamarn et al. [34]	2012	Thailand	Subjects with the criteria of prediabetes	M, F	56.95	12	57.93	12.71	119	116
Chuengsamarn et al. [35]	2014	Thailand	Type 2 Diabetes patients	M, F	59.16	11.03	59.58	10.71	107	106
Cicero et al. [36]	2019	Italy	Overweight subjects	M, F	54	3	53	5	40	40
Darmian et al. [37]	2022	Iran	Middle-aged women with hyperlipidemia and type 2 diabetes (Turmeric capsule group) Aerobic training group Aerobic training + Turmeric capsule group	F	44.33 42.13 43.02	1.23 2.39 3.04	44.22	3.07	11 10 11	10
Dolati et al. [38]	2020	Iran	Overweight women (Curcumin group) (Curcumin + Training group) (Placebo + training group)	F	38.9 35.80 38.20	5.4 3.22 5.67	40.8	3.55	10 10 10	10
Farzad et al. [39]	2020	Iran	Overweight women (Curcumin group) Curcumin + Training group Training group	F	38.9 35.8 38.2	5.4 3.22 5.67	40.6	3.71	10 10	10
Fergusona et al. [40]	2018	Australia	Patients with hypercholesterolemia (Curcumin group) (Phytosterol+ Curcumin group) (Phytosterol group)	M, F	51 50.35 51.35	2.34 3.36 3.62	50.11	2.96	18 17 17	18
Funamoto et al. [41]	2019	Japan	Patients with Impaired Glucose Tolerance and Non-Insulin-Dependent Diabetes Mellitus	M, F	70	6	69	7	15	18
Funamoto et al. [42]	2016	Japan	Patients with mild COPD	M, F	69.6	6.6	69.9	6.3	22	17
Funamoto et al. [43]	2022	Japan	Patients exhibiting initial signs of hypertensive heart disease	M, F	67 (median)	66 (median)	73	69		
Garg et al. [44]	2018	Canada	Patients with elective repair of an abdominal aortic aneurysm	M, F	76 (median)	304	302			
Ghaffari et al. [45]	2019	Iran	Patients with non-alcoholic fatty liver disease (Turmeric only group) (Chicory seed only group) (Turmeric + Chicory seed group)	M, F	42.5 41 41.5	6.93 8.61 7.68	40.3	9.26	21 21 21	21
Ghazimoradi et al. [46]	2017	Iran	Subjects with metabolic syndrome (Phosholipidated Curcumin group) (Curcumin group)	M/F	40.05 37.52	10.48 9.87	38.59	10.28	37 36	36
Hariri et al. [47]	2020	Iran	Non-Alcoholic Fatty Liver Disease Patient	M, F	40.95	12.24	40.06	13.69	23	22
Haroyan et al. [48]	2018	Armenia	Osteoarthritis patients (Curamed group) (Curcumin group)	M, F	54.65 57.91	8.84 9.02	56.04	8.55	57 66	54
Hellmann et al. [49]	2022	Denmark	Obese, non-diabetic individuals	M, F	44.8	15.8	47.7	12.1	18	19
Hellmann et al. [50]	2023	Denmark	Prednisolone-induced glucometabolic perturbations in men with overweight or obesity (Prednisolone + Curcumin placebo group) Prednisolone + Curcumin group	M	44 47	13.5 17.8	41.6	9.8	8 8	8
Heshmati et al. [51]	2020	Iran	Patients with Polycystic Ovary Syndrome	F	30.97	5.2	30.75	7.97	34	33
Heshmati et al. [52]	2020	Iran	Polycystic ovarian syndrome (PCOS) patients	F	31 (Median)	29 (Median)	34	33		
Hodge et al. [53]	2019	Iran	Type 2 Diabetes patients	M, F	58	8	60	7	21	23
Ismail et al. [54]	2014	Egypt	Obese Children	M, F	15.5714	5.7974	16.5357	8.69674	14	11
Ismail et al. [55]	2016	Egypt	Obese subjects (Pediatrics) (Adults)	M, F	14.707 37.552	4.52 9.934	14.707 37.552	4.52 9.552	15 15	14 14
Jamilian et al. [56]	2020	Iran	Women with polycystic ovary syndrome	F	28.6	4.7	27.2	3.4	24	26
Jarhahzaden et al. [57]	2021	Iran	Nonalcoholic fatty liver disease	M, F	44.12	8.35	38.56	10.43	32	32
Javandoosi et al. [58]	2018	Iran	Patients with metabolic syndrome (Curcumin group) (Complex Curcumin Group)	M, F	18–65 (range)				36 37	36
Jazayeri-Tehrani et al. [59]	2019	Iran	Overweight/obese patients with non-alcoholic fatty liver disease	M, F	41.8	5.6	42.5	6.2	42	42
Jimenez-Osorio et al. [60]	2016	Mexico	Patients with non-diabetic or diabetic proteinuria, chronic kidney disease (Diabetes Group) (Non-Diabetes Group)	M, F	55 36.8	1.6 2.7	56.2 44.3	1.5 3.4	28 24	23 26
Karandish et al. [61]	2021	Iran	Overweight or obese prediabetes subjects (Curcumin group) Zinc group Zinc + Curcumin group	M, F	36.95 38.19 34.48	7.23 4.87 6.45	34.19	7.03	21 21 20	20
Karandish et al. [62]	2022	Iran	overweight or obese patients with prediabetes (Curcumin group) Zinc group Curcumin + Zinc group	M, F	36.95 38.19 34.48	7.23 4.87 6.45	34.19	7.03	21 21 20	20
Kelardeh et al. [63]	2020	Iran	Older women with non-alcoholic fatty liver disease (Resistance training group) Curcumin group Resistance training + Curcumin group	F	65.91 66.72 64.09	3.31 3.03 3.03	64.36	2.97	12 11 11	11
Khajehdehi et al. [64]	2011	Iran	Type 2 Diabetes patients	M, F	52.9	9.2	52.6	9.7	20	20
Khajehdehi et al. [65]	2012	Iran	Patients with relapsing or refractory biopsy-proven lupus nephritis	M, F	32.2	11.4	35	10.4	12	12
Kisiolek et al. [66]	2021	USA	Healthy and physically active subjects (Curcumin Fast Cycling Time Trial Group) (Curcumin Slow Cycling Time Trial Group)	M, F	25 25.6	3.6 5.1	23.1	3.7	12 12	12
Kocher et al. [67]	2016	Germany	Hyperlipidemia individuals (Men) (Women)	M, F	50 52	20 16	500 52	20 16	N/M	N/M
Krishnareddy et al. [68]	2018	India	Healthy subjects with chronic alcohol intake	M	45	9.1	45	9.1	23	22
Majeed et al. [69]	2023	India	Obese adults	M, F	45.25	7.11	41.25	7.11	47	47
Mamsharifi et al. [70]	2023	Iran	Smokers	M, F	33.05	10.05	32.14	9.55	35	35
Mankowski et al. [71]	2022	USA	Moderately functioning older adults with low-grade inflammation	M, F	79.4	10.1	76.2	5.6	9	8
Mirhafez et al. [72]	2019	Iran	Non-Alcoholic Fatty Liver Disease Patient	M, F	44.8	11.14	40.7	11.83	32	29
Mirhafez et al. [73]	2019	Iran	Non-Alcoholic Fatty Liver Disease Patient	M, F	41.2	14.1	40.7	11	22	22
Mirhafez et al. [74]	2019	Iran	Patients with non-alcoholic fatty liver diseases	M, F	38.82	2.95	43.29	2.21	24	23
Mirhafez et al. [75]	2021	Iran	Non-Alcoholic Fatty Liver Disease patient	M, F	45	11.1	43.1	11.6	35	37
Mirzabeigi et al. [76]	2015	Iran	Volunteers >18 years old with a diagnosis of CAD	M, F	61.5	8.7	64.3	8.42	17	16
Mohammadi et al. [77]	2017	Iran	Individuals with metabolic syndrome (Curcumin phospholipid complex group) (Curcumin group)	M/F	40.05 37.52	10.48 9.87	38.59	10.28	37 36	36
Mohammadi et al. [78]	2024	Iran	Patients with metabolic syndrome (Curcumin group) Probiotic group Curcumin + Probiotic group	M, F	49.39 49.5 47.39	1.31 1.13 1.67	48.96	1.28	28 30 28	28
Mokhtari et al. [79]	2020	Iran	Patients with diabetic foot ulcer	M, F	57.4	11.7	55.8	9.4	25	25
Na et al. [80]	2012	China	Overweight or Obese with T2D Patients	M, F	55.42	6.4	54.72	8.34	50	50
Neta et al. [81]	2021	Brazil	Type 2 Diabetes patients	M, F	63.1	11.1	61.9	11	33	28
Nowak et al. [82]	2022	USA	Autosomal dominant polycystic kidney disease patients	M, F	18	6	19	5	28	29
Osali et al. [83]	2020	Iran	Elderly female with metabolic syndrome (Exercise group) Nano-curcumin group) Exercise + Nano-curcumin group)	F	62.3	1.23	62.3	1.23	11 11 11	11
Panahi et al. [84]	2018	Iran	Patients with type 2 Diabetes Mellitus	M, F	43	8	41	7	50	50
Panahi et al. [85]	2014	Iran	Patients with metabolic syndrome	M, F	44.8	8.67	43.46	9.7	50	50
Panahi et al. [86]	2015	Iran	Metabolic syndrome patients	M, F	44.8	8.67	43.46	9.7	50	50
Panahi et al. [87]	2015	Iran	Osteoarthritis patients	M, F	57.32	8.78	57.57	9.05	19	21
Panahi et al. [88]	2016	Iran	Type 2 Diabetes Patients	M, F	43	8	41	7	N/M	N/M
Panahi et al. [89]	2016	Iran	Non-Alcoholic Fatty Liver Disease Patients	M, F	44.98	12.59	47.21	10.29	44	43
Panahi et al. [90]	2016	Iran	Subjects with metabolic syndrome	M, F	44.8	8.67	43.46	9.7	50	50
Panahi et al. [91]	2017	Iran	Type 2 Diabetes Patients	M, F	43	8	41	7	50	50
Panahi et al. [92]	2017	Iran	Non-Alcoholic Fatty Liver Disease Patients	M, F	44.98	12.59	47.21	10.29	44	43
Pashine et al. [93]	2012	India	Overweight hyperlipidemia subjects	M, F	21–60	53	52			
Pierro et al. [94]	2015	Italy	Overweight people with metabolic syndrome	M, F	39.1	16.8	41.85	15.91	22	22
Porasgari et al. [95]	2022	Iran	Overweight and obese women (Curcumin group) Pilates group Curcumin + Pilates group	F	36.37 36.62 37.37	21.97 19.47 24.99	37.75	22.73	14 14 15	13
Rahimi et al. [96]	2016	Iran	Diabetic subjects	M, F	56.34	11.17	60.95	10.77	35	35
Rahmani et al. [97]	2016	Iran	Patients with symptoms of metabolic syndrome	M, F	46.37	11.57	48.95	9.78	37	40
Reis et al. [98]	2022	Brazil	Brazilian women	F	47	10.52	50	12.58	15	20
Rezaei et al. [99]	2024	Iran	Overweight or obese patients with the coronary slow flow phenomenon	M, F	54.3	9.1	54.6	8.4	21	21
Saadati et al. [100]	2019	Iran	Non-Alcoholic Fatty Liver Disease Patient	M, F	11.5	46.19	10.9	45.13	25	23
Saadati et al. [101]	2019	Iran	Patients with non-alcoholic fatty liver disease	M, F	46.19	11.5	45.13	10.9	27	23
Saberi-Karimian et al. [102]	2018	Iran	Patients with metabolic syndrome (Curcumin phospholipid group) (Curcumin group)	N/M	40.05 37.52	10.48 9.47	38.59	10.28	37 36	36
Saberi-Karimian et al. [103]	2019	Iran	Metabolic syndrome patients (Curcumin phospholipid group) (Curcumin group)	M, F	40.05 37.52	10.48 9.47	38.59	10.28	37 36	36
Saberi-Karimian et al. [104]	2020	Iran	Non-Alcoholic Fatty Liver Disease Patients	M, F	18–70	23	26			
Sadeghzadeh et al. [105]	2023	Iran	Postmenopausal women (Curcumin Group) Nigella sativa Group Curcumin + Nigella sativa Group	F	58 57.2 57.4	3.4 4.3 3.8	58.4	3.4	30 28 28	29
Sangouni et al. [106]	2022	Iran	Subjects with metabolic syndrome (Curcumin CP group) Coenzyme + Placebo QP group Curcumin + Coenzyme CQ group	M, F	38.8 39 37.7	4.9 4.5 5	39.5	5	22 22 22	22
Saraf-Bank et al. [107]	2019	Iran	Overweight adolescent girls	F	16.03	1.56	15.98	1.72	30	30
Saraf-Bank et al. [108]	2019	Iran	Healthy overweight and obese adolescent girls	F	16.03	1.56	15.98	1.72	30	30
Sedighiyan et al. [109]	2022	Iran	Obese and overweight patients with migraine	M, F	39.27	10.07	41	11.35	22	22
Shafabakhsh et al. [110]	2020	Iran	T2D Mellitus and coronary heart disease patients	M, F	64.9	7.8	66.5	7.7	25	24
Shirmohammadi et al. [111]	2019	Iran	Patients with metabolic syndrome	M, F	40.05	10.48	38.59	10.28	37	36
Sohaei et al. [112]	2019	Iran	Overweight and obese women with PCOS	F	29.4	5.33	29.58	5	27	24
Soltani et al. [113]	2024	Iran	Overweight or obese patients with the coronary slow flow phenomenon	M, F	54	9	55	8	21	21
Srinivasan et al. [114]	2019	India	Type 2 Diabetes patients	M, F	51.32	8.61	49.94	8.72	60	54
Tamaddoni et al. [115]	2019	Iran	Beta thalassemia major patients	M, F	25.97	6.92	27.61	6.23	31	30
Thota et al. [116]	2020	Australia	Adults with high risk of T2 Diabetes and Alzheimer’s disease	M, F	54.5	2.9	50.4	2.6	14	15
Uchio et al. [117]	2019	Japan	Subjects with overweight or prehypertension/mild hypertension	M, F	58.8	5.3	58.5	5.5	43	44
Yaikwawong et al. [118]	2024	Thailand	Type 2 Diabetes Patients	M, F	60.27	8.82	62.26	8.65	113	114
Yaikwawong et al. [119]	2024	Thailand	Obese patients with Type 2 Diabetes	M, F	60.27	8.82	62.26	8.65	113	114
Yang et al. [120]	2014	Taiwan	Metabolic syndrome patients	M, F	59.03	10.1	59.61	14.09	30	29
Zohrabi et al. [121]	2023	Iran	Polycystic ovary syndrome patients (Curcumin group) Curcumin + DASH diet group DASH diet group	F	18–45	25 24 24	24			

**Table 3:** Pooled Effects of *Curcuma longa* (Curcumin) Supplementation on Clinical and Biochemical Parameters.

Outcome Domain	Parameter	Pooled SMD (95% CI)	*p*-Value	I^2^ (%)	Remarks
Anthropometry	BMI	−0.27 (−0.57 to 0.02)	>0.05	79	No significant effect; heterogeneous results
	Waist Circumference (WC)	−0.33 (−0.81 to 0.15)	>0.05	75	Non-significant; trend toward reduction
	Waist–Hip Ratio (WHR)	−0.11 (−0.37 to 0.15)	>0.05	60+	No effect
Blood Pressure	Systolic BP (SBP)	−0.65 (−1.21 to −0.08)	<0.05	65	Significant SBP reduction (mainly in MetS)
	Diastolic BP (DBP)	−0.40 (−0.79 to −0.01)	<0.05	63	Mild DBP reduction; modest heterogeneity
Glycemic Control	Fasting Blood Sugar (FBS)	−0.25 (−0.48 to −0.03)	<0.05	55	Small but significant reduction
	Blood Glucose	−0.53 (−0.82 to −0.23)	<0.05	58	Moderate improvement
	HbA1c	−0.33 (−0.58 to −0.09)	<0.05	66	Significant improvement in long-term control
Insulin Function	HOMA-IR	−0.01 (−0.78 to 0.76)	>0.05	95	No consistent effect; highly heterogeneous
	HOMA-B	0.09 (−0.19 to 0.36)	>0.05	0	No effect on β-cell function
	QUICKI	0.41 (−0.39 to 1.21)	>0.05	80+	Non-significant; high variability
	Serum Insulin	−0.33 (−0.77 to 0.11)	>0.05	75	No consistent reduction
Lipid Profile	Total Cholesterol (TC)	−0.22 (−0.45 to 0.01)	>0.05	60+	No consistent change
	LDL-C	−0.36 (−0.64 to −0.07)	<0.05	56	Modest reduction (not uniform)
	HDL-C	0.40 (0.03 to 0.77)	<0.05	72	Significant increase
	Triglycerides (TG)	−0.27 (−0.49 to −0.05)	<0.05	68	Mild reduction, especially in NAFLD
Inflammation	CRP	−0.39 (−0.64 to −0.14)	<0.05	40	Consistent anti-inflammatory effect
	TNF-α	−1.07 (−2.05 to −0.09)	<0.05	91	Large but heterogeneous reduction
Oxidative Stress	TAC	0.68 (−0.30 to 1.67)	>0.05	85	Inconsistent antioxidant improvement
	GSH	0.91 (−0.88 to 2.69)	>0.05	92	Non-significant; sparse data
	MDA	−0.22 (−0.54 to 0.09)	>0.05	45	No consistent effect

Curcumin supplementation shows modest, clinically small but statistically significant improvements in fasting glucose, HbA1c, HDL-C, triglycerides, CRP, and blood pressure (particularly SBP). Effects on weight, insulin sensitivity, total/LDL cholesterol, and oxidative markers remain inconclusive due to heterogeneity and formulation variability.

**Table 4:** Overall quality of evidence (GRADE) for outcomes included in the meta-analysis.

Outcome Category	Specific Outcomes	No. of Trials (Approx.)	Effect Direction	Certainty (GRADE)	Reasons for Downgrading
Glycemic control	Fasting blood sugar (FBS)	18	↓ Significant reduction	Moderate	Moderate heterogeneity, some unclear RoB
	HbA1c	10	↓ Significant reduction	Moderate	Some inconsistency and small-trial effects
Insulin resistance indices	HOMA-IR, HOMA-B, fasting insulin, QUICKI	15	↔/↓ Inconsistent or null	Low–Very Low	Very high heterogeneity, imprecision, small samples
Lipid profile	Triglycerides (TG)	16	↓ Significant reduction	Moderate	Moderate heterogeneity (dose/formulation)
	Total cholesterol (TC)	15	↓ Modest reduction	Low–Moderate	Heterogeneity and imprecision
	LDL-C	14	↓ Inconsistent reduction	Low	Small-study bias, variable results
	HDL-C	14	↑ Significant increase	Moderate	Moderate heterogeneity
Anthropometric measures	BMI	17	↓ Slight reduction	Low	Short intervention durations, small samples
	Waist circumference	14	↓ Moderate reduction	Moderate	Some heterogeneity, limited long-term data
	Waist-to-hip ratio	7	↓ Minimal reduction	Low	Imprecision and limited sample size
Inflammatory markers	C-reactive protein (CRP)	18	↓ Significant reduction	Moderate	High heterogeneity, possible publication bias
	TNF-α	10	↓ Moderate reduction	Low	Fewer studies, wide CIs
Oxidative stress markers	Total antioxidant capacity (TAC)	7	↑ Moderate increase	Low	Inconsistent assays, small samples
	Glutathione (GSH)	4	↑ Inconsistent	Very Low	Scarce data, high heterogeneity
	Malondialdehyde (MDA)	5	↓ Inconsistent	Low	Imprecision, limited data

Abbreviations: RoB = risk of bias; CI = confidence interval; HOMA-IR = homeostatic model assessment of insulin resistance; QUICKI = quantitative insulin sensitivity check index; TAC = total antioxidant capacity; GSH = glutathione; MDA = malondialdehyde; LDL-C = low-density lipoprotein cholesterol; HDL-C = high-density lipoprotein cholesterol; ↑ = increase; ↓ = decrease. ↔ = no difference.

## Data Availability

No new data were created or analyzed in this study.

## Supplementary Materials

The following supporting information can be downloaded at: https://www.mdpi.com/article/10.3390/foods15010060/s1.

## Abbreviations

The following abbreviations are used in this manuscript:

BMI	Body mass index
BP	Blood pressure
CAT	Catalase
CCNP	Curcumin Nanoparticle
CRP	C-reactive protein
Glu	Glucose
GSH	Glutathione
GST	Glutathione S-Transferase
Hb1Ac	Hemoglobin A1C
HDL	High-density lipoprotein
HFD	High fat diet
IL-10	Interleukin-10
IL-12	Interleukin-12
IL-1β	Interleukin-1beta
IL-6	Interleukin-6
INF-γ	Interferon-gamma
INS	Insulin
LDL	Low-density lipoprotein
MDA	Malondialdehyde
MMP-9	Matrix metalloproteinase-9
MPO	Myeloperoxidase
NAFLD	Non-alcoholic fatty liver disease
NCUR	Nanocurcumin
NFκB	Nuclear factor kappa-light-chain-enhancer of activated B cells
NO	Nitric oxide
NOx	Total nitrites and nitrates
SOD	Superoxide dismutase
TAC	Total antioxidant capacity
TC	Total cholesterol
TG	Triglyceride
