# Supplementary material for: Clinical Potential of Curcuma longa Linn. as Nutraceutical/Dietary Supplement for Metabolic Syndrome: Systematic Review and Meta-Analysis of Randomized Controlled Trials
Source: Foods. 2025 Dec 24;15(1):60. doi: 10.3390/foods15010060 (PMC12785604; doi:10.3390/foods15010060)
Supplement: Supplementary file 1 [file foods-15-00060-s001.zip › foods-4017895-supplementary.pdf]

**Supplementary Table S1**

**Database search strategy for in Clinical Randomized Control Trial studies investigating the beneficial effects of Curcumin/*Curcuma longa* extracts consumption on metabolic syndrome.**

| Database | Keywords | Results |
|---|---|---|
| PudMed (2024.08.23) | In this database, applied filters are - full-text, clinical trial, Randomized Controlled Trial and Clinical Study.<br>KP-1: ((curcumin [Title]) OR (*Curcuma longa* [Title]) OR (diferuloylmethane [Title])) AND (Clinical [Title/Abstract]) OR (randomized control [Title/ Abstract])) OR (RCT[Title/Abstract]) AND ((metabolic syndromes [Title/ Abstract]) AND (insulin resistance [Title/Abstract]) OR (Hyperinsulinemia [Title/Abstract]) AND (type 2 diabetes [Title/Abstract]) | 105 |
| | KP-2: ((curcumin [Title]) OR (*Curcuma longa* [Title]) OR (diferuloylmethane [Title)) AND (Clinical [Title/Abstract]) OR (randomized control [Title/Abstract])) OR (RCT[Title/Abstract]) AND ((metabolic syndromes [Title/Abstract]) AND (obesity [Title/Abstract]) OR (abdominal obesity [Title/Abstract]) OR (hyperlipidemia [Title/Abstract]) AND (Dyslipidemia [Title/Abstract]) | 170 |
| | KP-3: (((curcumin [Title] OR curcuma [Title]) OR (diferuloylmethane [Title])) AND (Clinical [Title/Abstract]) OR (randomized control [Title/Abstract])) OR (RCT[Title/Abstract]) AND ((metabolic syndromes [Title/Abstract]) AND (hypertension [Title/Abstract] OR (high blood pressure [Title/Abstract] AND (cardiovascular diseases [Title/Abstract]) | 26 |
| | KP-4: ((curcumin [Title/Abstract]) OR (*Curcuma longa* [Title/Abstract]) OR (diferuloylmethane [Title/Abstract])) AND (Clinical [Title/Abstract]) OR (randomized control [Title/Abstract])) OR (RCT[Title/Abstract]) AND ((metabolic syndromes [Title/Abstract]) OR (heart diseases [Title/Abstract]) AND (hypertension [Title/Abstract]) AND (stroke [Title/Abstract]) | 1 |
| | KP-5:((curcumin [Title/Abstract]) OR (curcuma [Title/Abstract]) OR (diferuloylmethane [Title/Abstract])) AND (Clinical [Title/Abstract]) OR (randomized control [Title/Abstract])) OR (RCT[Title/Abstract]) AND ((metabolic syndromes [Title/Abstract]) AND (Abnormal cholesterol levels [Title/Abstract]) OR (hypercholesterolemia [Title/Abstract]) AND (cholesterol profile [Title/Abstract]) | 1 |
| Scopus (2024.08.28) | In this database, applied filters are - Article Type and Only English Article.<br>KS-1: (TITLE-ABS-KEY (curcumin) OR TITLE-ABS-KEY (diferuloylmethane) OR (TITLE-ABS-KEY (curcuma longa) AND TITLE-ABS-KEY (Clinical) OR TITLE-ABS-KEY (RCT) OR TITLE-ABS-KEY (Randomized Trial) AND TITLE-ABS-KEY (metabolic syndromes) OR TITLE-ABS-KEY (insulin resistance) OR TITLE-ABS-KEY (type 2 diabetes) OR TITLE-ABS-KEY (hyperglycemia) | 290 |
| | KS -2: (TITLE-ABS-KEY (curcumin) OR (TITLE-ABS-KEY (curcuma longa) OR TITLE-ABS-KEY (diferuloylmethane) AND TITLE-ABS-KEY (Clinical) OR TITLE-ABS-KEY (RCT) OR TITLE-ABS-KEY (Randomized Trial) AND TITLE-ABS-KEY (metabolic syndromes) AND TITLE-ABS-KEY (obesity) OR TITLE-ABS-KEY (hyperlipidemia) OR TITLE-ABS-KEY (dyslipidemia) OR TITLE-ABS-KEY (abdominal obesity)] | 40 |

| | | |
|---|---|---|
| | KS-3: (TITLE-ABS-KEY (curcumin) OR (TITLE-ABS-KEY (curcuma longa) OR TITLE-ABS-KEY (diferuloylmethane) AND TITLE-ABS-KEY (Clinical) OR TITLE-ABS-KEY (RCT) OR TITLE-ABS-KEY (Randomized Trial) AND TITLE-ABS-KEY (metabolic syndromes) AND TITLE-ABS-KEY (hypertension) OR TITLE-ABS-KEY (high blood pressure) OR TITLE-ABS-KEY (cardiovascular diseases) | 7 |
| | KS-4: (TITLE-ABS-KEY (curcumin) OR TITLE-ABS-KEY (diferuloylmethane) OR TITLE-ABS-KEY (curcuma longa) AND TITLE-ABS-KEY (Clinical) OR TITLE-ABS-KEY (RCT) OR TITLE-ABS-KEY (Randomized Trial) AND TITLE-ABS-KEY (metabolic syndromes) AND TITLE-ABS-KEY (heart disease) OR TITLE-ABS-KEY (stroke) | 43 |
| | KS-5: (TITLE-ABS-KEY (curcumin) OR TITLE-ABS-KEY (diferuloylmethane) OR TITLE-ABS-KEY (curcuma longa) AND TITLE-ABS-KEY (Clinical) OR TITLE-ABS-KEY (RCT) OR TITLE-ABS-KEY (Randomized Trial) AND TITLE-ABS-KEY (metabolic syndromes) OR TITLE-ABS-KEY (abnormal cholesterol levels) OR TITLE-ABS-KEY (cholesterol profile) OR TITLE-ABS-KEY (hypercholesterolemia) | 179 |
| AMED (2024.08.28) | In this database, all the searches use<br>Limiters - Full Text; English Language; Exclude Pre-CINAHL<br>Expanders - Apply equivalent subjects.<br>Search modes - SmartText Searching<br>KA-1: TITLE curcumin or *Curcuma longa* or diferuloylmethane AND AB (clinical or RCT or Randomized Trial) AND AB metabolic syndrome OR AB insulin resistance OR AB hyperglycemia OR AB type 2 diabetes | 27 |
| | KA-2: TITLE curcumin or *Curcuma longa* or diferuloylmethane AND AB (clinical or RCT or Randomized Trial) AND AB metabolic syndromes OR AB obesity OR AB abdominal obesity OR AB hyperlipidemia OR AB dyslipidemia | 27 |
| | KA-3: TITLE curcumin or *Curcuma longa* or diferuloylmethane AND AB (clinical or RCT or Randomized Trial) AND AB metabolic syndromes OR AB hypertension OR AB high blood pressure OR AB cardiovascular diseases | 27 |
| | KA-4: TITLE curcumin or *Curcuma longa* or diferuloylmethane AND AB (clinical or RCT or Randomized Trial) AND AB metabolic syndromes OR AB heart diseases OR AB stroke | 27 |
| | KA-5: TITLE curcumin or *Curcuma longa* or diferuloylmethane AND AB (clinical or RCT or Randomized Trial) AND AB metabolic syndromes OR AB abnormal cholesterol levels OR AB cholesterol profile OR AB Hypercholesterolemia | 27 |
| LILACS (2024.08.30) | From this database, all the searches use the filters Full-text, Curcumin and Curcuma and Controlled Clinical Trial and English.<br>KL-1: Title, abstract, subject: curcumin or *Curcuma longa* or diferuloylmethane and clinical or RCT or randomized clinical trial and metabolic syndromes or insulin resistance or type 2 diabetes or hyperglycemia | 377 |
| | KL-2: Title, abstract, subject: curcumin or *Curcuma longa* or diferuloylmethane and clinical or RCT or randomized clinical trial and metabolic syndromes and | 15 |

| | | |
|---|---|---|
| | obesity or hyperlipidemia or abdominal obesity or dyslipidemia | |
| | KL-3: Title, abstract, subject: curcumin or *Curcuma longa* or diferuloylmethane and clinical or RCT or randomized clinical trial and metabolic syndromes or hypertension or high blood pressure or cardiovascular diseases | 377 |
| | KL-4: Title, abstract, subject: curcumin or *Curcuma longa* or diferuloylmethane and clinical or RCT or randomized clinical trial and metabolic syndromes and heart diseases or stroke | 377 |
| | KL-5: Title, abstract, subject: curcumin or *Curcuma longa* or diferuloylmethane and clinical or RCT or randomized clinical trial and metabolic syndromes and abnormal cholesterol levels or cholesterol profile or hypercholesterolemia | 15 |
| MDPI (2024.08.28) | In this database, all the searches use the filters Document type- articles.<br>KM-1: Title: curcumin or diferuloylmethane or *Curcuma longa* and Abstract: Clinical or RCT or Randomized Clinical Trial and metabolic syndromes and Keywords: insulin resistance or type 2 diabetes and hyperglycemia | 29 |
| | KM-2: Title: curcumin or diferuloylmethane or *Curcuma longa* and Abstract: Clinical or RCT or Randomized Clinical Trial and metabolic syndromes and obesity or abdominal obesity and hyperlipidemia | 6 |
| | KM-3: Title: curcumin or diferuloylmethane or *Curcuma longa* and Abstract: Clinical or RCT or Randomized Clinical Trial and metabolic syndromes and hypertension or high blood pressure and cardiovascular diseases | 50 |
| | KM-4: Title: curcumin or diferuloylmethane or *Curcuma longa* and Abstract: Clinical or RCT or Randomized Clinical Trial and metabolic syndromes or Keywords: heart diseases and stroke | 28 |
| | KM-5: Title: curcumin or diferuloylmethane or *Curcuma longa* and Abstract: Clinical or RCT or Randomized Clinical Trial and metabolic syndromes and hypercholesterolemia and Full text: abnormal cholesterol levels or cholesterol Profile | 6 |
| Google Scholar (2024.09.02) | Only English and relevant articles are deducted. Other irrelevant articles are removed and excluded.<br>Main keywords- Curcumin, Clinical or RCT or Randomized Controlled Trial.<br>Exclude the words - review, meta-analysis, overview, rat, mice, vivo, animal<br>Exact Word - Curcumin<br>Method - Title Screening<br>Additional Keywords for each search: Insulin Resistance, Type-2-diabetes, Diabetes, Metabolic syndrome, Obesity, Abdominal obesity, Hypertension, Stroke, heart disease, cholesterol, Cardiovascular, Glucose, Blood Pressure, Glycemic, obese. | 89 |

**Supplementary Table S2**
**Risk of Bias Assessment of the Included Studies**

| Author, Year | Selection Bias | | | Performance Bias | | | Attribution Bias | | Other Bias | Overall Bias |
|---|---|---|---|---|---|---|---|---|---|---|
| | D1 | D2 | D3 | D4 | D5 | D6 | D7 | D8 | D9 | D10 |
| Abed & Abdulridha [18] | Unclear | Unclear | Low | High | High | Low | Low | Low | Low | High |
| Adibian et al. [19] | Low | Low | Low | Low | Low | Low | Low | Low | Low | Low |
| Afshar et al. [20] | Low | Unclear | Low | Low | Low | Low | Low | Low | Low | Low |
| Alizadeh et al. [21] | Low | Low | Low | Low | Low | Low | Low | Low | Low | Low |
| Alvarenga et al. [22] | Low | Low | Low | Low | Low | Low | Low | Low | Low | Low |
| Alvarenga et al. [23] | Low | Low | Unclear | Low | Low | Low | Low | Low | Low | Low |
| Asadi et al. [24] | Low | Unclear | Low | Low | Low | Low | Low | Low | Low | Low |
| Asan et al. [25] | Unclear | Unclear | Low | Low | High | Low | Unclear | Low | Low | High |
| Asghari et al. [26] | Low | Low | Low | Low | Low | Low | Low | Low | Low | Low |
| Askari et al. [27] | Low | Low | Low | Low | Low | Low | Low | Low | High | Unclear |
| Atakan et al. [28] | Low | Unclear | Low | Unclear | Unclear | Low | High | Low | Low | High |
| Barber-Chamoux et al. [29] | Low | Low | Unclear | Low | Low | Low | Low | Low | Low | Low |
| Bateni et al. [30] | Low | Unclear | Low | Low | Low | Low | Low | Low | Low | Low |
| Boshagh et al. [31] | Low | Low | Low | Low | Low | Low | Low | Low | High | Unclear |
| Campbell et al. [32] | Unclear | Unclear | Unclear | Low | Low | Low | Low | Low | Low | Unclear |
| Chashmniam et al. [33] | Low | Low | Unclear | Low | Low | Low | High | High | Low | High |
| Chuengsamarn et al. [34] | Low | Low | Low | Low | Low | Low | Low | Low | Low | Low |
| Chuengsamarn et al. [35] | Low | Low | Low | Low | Low | Low | Low | Low | Low | Low |
| Cicero et al. [36] | Low | Low | Low | Low | Low | Low | Low | Low | Low | Low |
| Darmian et al. [37] | Low | Unclear | Low | Low | High | Low | Low | Low | Low | Unclear |
| Dolati et al. [38] | Low | Unclear | Low | Low | Low | Low | Low | Low | Low | Low |
| Dolati et al. [39] | Low | Unclear | Low | Low | Low | Low | Low | Low | Low | Low |
| Fergusona et al. [40] | Low | Low | Low | Low | Low | Low | Low | Low | Low | Low |
| Funamoto et al. [41] | Unclear | Unclear | Unclear | Low | Low | Low | High | High | High | High |
| Funamoto et al. [42] | Low | Low | Low | Low | Low | Low | High | High | High | High |
| Funamoto et al. [43] | Low | Unclear | Low | Low | Low | Low | Low | Low | High | Unclear |
| Garg et al. [44] | Low | Unclear | Low | Low | Low | Low | Low | Low | Low | Low |
| Ghaffari et al. [45] | Low | Low | Low | Low | Low | Low | Low | Low | Low | Low |
| Ghazimoradi et al. [46] | Low | Unclear | Low | Low | Low | Low | Low | Low | Low | Low |
| Hariri et al. [47] | Low | Low | Low | Low | Low | Low | Low | Low | Low | Low |
| Haroyan et al. [48] | Low | Low | Low | Low | Low | Low | Low | Low | Low | Low |
| Hellmann et al. [49] | Low | Low | Low | Low | Low | Low | Low | Low | Low | Low |

| | | | | | | | | | | |
|---|---|---|---|---|---|---|---|---|---|---|
| Hellmann et al. [50] | Low | Low | Low | Low | Low | Low | Low | Low | Low | Low |
| Heshmati et al. [51] | Low | Low | Low | Low | Low | Low | Low | Low | Low | Low |
| Heshmati et al. [52] | Low | Unclear | Low | Low | Low | Low | Low | Low | Low | Low |
| Hondaei et al. [53] | Low | Low | Low | Low | Low | Low | Low | Low | Low | Low |
| Ismail et al. [54] | Unclear | High | Low | High | High | Low | Low | Low | Low | High |
| Ismail et al. [55] | Unclear | Unclear | Low | High | High | Low | Low | Low | Low | High |
| Jamilian et al. [56] | Low | Unclear | Low | Low | Low | Low | Low | Low | Low | Low |
| Jarhahzaden et al. [57] | Unclear | Unclear | Low | Low | Low | Low | Low | Low | Low | Unclear |
| Javandoosi et al. [58] | Unclear | Unclear | Unclear | Low | Low | Low | Low | Low | Low | Unclear |
| Jazayeri-Tehrani et al. [59] | Low | Low | Low | Low | Low | Low | Low | Low | Low | Low |
| Jimenez-Osorio et al. [60] | Unclear | Unclear | Unclear | Low | Low | Low | Low | Low | Low | Unclear |
| Karandish et al. [61] | Low | Low | Low | Low | Low | Low | Low | Low | Low | Low |
| Karandish et al. [62] | Low | Low | Low | Low | Low | Low | Low | Low | Low | Low |
| Kelardeh et al. [63] | Low | Low | Low | Low | Low | Low | Low | Low | Low | Low |
| Khajehdehi et al. [64] | Unclear | Low | Low | Low | Low | Low | Low | Low | Low | Low |
| Khajehdehi et al. [65] | Unclear | Low | Low | Low | Low | Low | Low | Low | Low | Low |
| Kisiolek et al. [66] | Unclear | Unclear | Low | Low | Low | Low | Low | Low | Low | Unclear |
| Kocher et al. [67] | Unclear | Unclear | Unclear | Low | Low | Low | Low | Low | High | High |
| Krishnareddy et al. [68] | Low | Low | Low | Low | Low | Low | Low | Low | Low | Low |
| Majeed et al. [69] | Low | Low | Low | Low | Low | Low | Low | Low | Low | Low |
| Mamsharifi et al. [70] | Low | Low | Low | Low | Low | Low | Low | Low | Low | Low |
| Mankowski et al. [71] | Unclear | Unclear | Low | Unclear | Unclear | Low | Low | High | Low | High |
| Mirhafez et al. [72] | Low | High | Unclear | Low | Low | Low | Low | Low | Low | Unclear |
| Mirhafez et al. [73] | Low | Unclear | Low | Low | Low | Low | Low | Low | Low | Low |
| Mirhafez et al. [74] | Low | Low | Low | Low | Low | Low | Low | Low | Low | Low |
| Mirhafez et al. [75] | Low | Low | Low | Low | Low | Low | Low | Low | Low | Low |
| Mirzabeigi et al. [76] | Unclear | Unclear | Low | Low | Low | Low | Low | Low | Low | Unclear |
| Mohammadi et al. [77] | Unclear | Unclear | Low | Unclear | Unclear | Low | Low | Low | Low | Unclear |
| Mohammadi et al. [78] | Low | Low | Unclear | Low | Low | Low | Low | Low | Low | Low |
| Mokhtari et al. [79] | Low | Low | Low | Low | Low | Low | Low | Low | Low | Low |
| Na et al. [80] | Low | Low | Low | Low | Low | Low | Low | Low | Low | Low |
| Neta et al. [81] | Low | Low | Low | Low | Low | Low | High | High | Low | High |
| Nowak et al. [82] | Low | Low | Low | Low | Low | Low | Low | Low | Low | Low |
| Osali [83] | Unclear | Unclear | Unclear | Low | Low | Low | Unclear | Unclear | Low | High |
| Panahi et al. [84] | Unclear | Unclear | Unclear | Low | Low | Low | Low | Low | Low | Unclear |
| Panahi et al. [85] | Unclear | Unclear | Low | Low | Low | Low | Low | Low | High | High |
| Panahi et al. [86] | Low | Unclear | Low | Low | Low | Low | High | High | Low | High |

| | | | | | | | | | | |
|---|---|---|---|---|---|---|---|---|---|---|
| Panahi et al. [87] | Unclear | Unclear | Low | Low | Low | Low | Unclear | Unclear | High | High |
| Panahi et al. [88] | Unclear | Unclear | Unclear | High | High | Low | Low | Low | Low | High |
| Panahi et al. [89] | Unclear | Unclear | Unclear | Low | Low | Low | Low | Low | High | High |
| Panahi et al. [90] | Unclear | Unclear | Unclear | Low | Low | Low | Low | Low | High | High |
| Panahi et al. [91] | Unclear | Unclear | Unclear | Unclear | Unclear | Low | Low | Low | Low | High |
| Panahi et al. [92] | Unclear | Unclear | Low | Low | Low | Low | Low | Low | High | High |
| Pashine et al. [93] | Low | Unclear | Low | Low | Low | Low | Low | Low | Unclear | Unclear |
| Pierro et al. [94] | Low | Low | Low | High | High | Low | Low | Low | High | High |
| Porasgari et al. [95] | Low | Unclear | Low | Low | Low | Low | Low | Low | Low | Low |
| Rahimi et al. [96] | Low | Unclear | Low | Low | Low | Low | Low | Low | Low | Low |
| Rahmani et al. [97] | Unclear | Unclear | Unclear | Low | Low | Low | Low | Low | Low | Unclear |
| Reis et al. [98] | Low | Low | Low | Low | High | Low | High | High | Low | High |
| Rezaei et al. [99] | Low | Low | Low | Low | Low | Low | Low | Low | Low | Low |
| Saadati et al. [100] | Low | Low | Low | Low | Low | Low | Low | Low | Low | Low |
| Saadati et al. [101] | Low | Unclear | Low | Low | Low | Low | Low | Low | Low | Low |
| Saberi-Karimian et al. [102] | Unclear | Unclear | Low | Low | Low | Low | Low | Low | Low | Unclear |
| Saberi-Karimian et al. [103] | Low | Unclear | Low | Low | Low | Low | Low | Low | Low | Low |
| Saberi-Karimian et al. [104] | Low | Low | Low | Low | Low | Low | Low | Low | Low | Low |
| Sadeghzadeh et al. [105] | Low | Low | Unclear | Low | Low | Low | Low | Low | Low | Low |
| Sangouni et al. [106] | Low | Low | Low | Low | Low | Low | Low | Low | Low | Low |
| Saraf-Bank et al. [107] | Low | Low | Low | Low | Low | Low | Low | Low | Low | Low |
| Saraf-Bank et al. [108] | Low | Low | Low | Low | Low | Low | Low | Low | Low | Low |
| Sedighiyan et al. [109] | Low | Low | Low | Low | Low | Low | Low | Low | Low | Low |
| Shafabakhsh et al. [110] | Unclear | Low | Low | Low | Low | Low | Low | Low | Low | Low |
| Shirmohammadi et al. [111] | Unclear | Low | Low | Low | Low | Low | Low | Low | Low | Low |
| Sohaei et al. [112] | Low | Low | Low | Low | Low | Low | Low | Low | Low | Low |
| Soltani et al. [113] | Low | Low | Low | Low | Low | Low | Low | Low | Low | Low |
| Srinivasan et al. [114] | Low | Low | Low | Low | Low | Low | Low | Low | Low | Low |
| Tamaddoni et al. [115] | Low | Low | Low | Low | Low | Low | Low | Low | Low | Low |
| Thota et al. [116] | Low | Unclear | Low | Low | Low | Low | Low | Low | Low | Low |
| Uchio et al. [117] | Low | Low | Low | Low | Low | Low | Low | Low | High | Unclear |
| Yaikwawong et al. [118] | Low | Low | Low | Low | Low | Low | Low | Low | Low | Low |
| Yaikwawong et al. [119] | Low | Low | Low | Low | Low | Low | Low | Low | Low | Low |
| Yang et al. [120] | Low | Low | Low | Low | Low | Low | Low | Low | Low | Low |
| Zohrabi et al. [121] | Low | Low | Low | Low | Low | Low | Low | Low | Low | Low |

**Supplementary Table S3**

**Treatment protocols of Eligible Randomized Controlled Trial Clinical Studies**

| Author, Year | Formulation | Treatment | Dosage | Duration |
|---|---|---|---|---|
| Abed & Abdulridha [18] | Capsules | Each capsule contains 750mg Curcumin | 2 capsules daily | 3 months |
| Adibian et al. [19] | Capsules | Each 500mg curcumin capsule contains 440 mg of curcuminoids (347 mg of curcumin + 84mg of DMC + 9mg of BDMC) + 38 mg of turmeric oil | 3 capsules per day | 10 weeks |
| Afshar et al. [20] | Nano | Nano-curcumin supplement | 120 mg divided into 3 capsules daily | 12 weeks |
| Alizadeh et al. [21] | Nano | 80 mg curcumin Nano-micelle | 80 mg daily | 10 weeks |
| Alvarenga et al. [22] | Juice | Juice containing 100mL of orange juice, 12g of carrot, and 2.5g of turmeric (95% curcumin) | 3 times a week | 12 weeks |
| Alvarenga et al. [23] | Juice | Juice containing 100mL of orange juice, 12g of carrots, and 2.5g of turmeric extract (95% of curcumin) | 3 times per week | 12 weeks |
| Asadi et al. [24] | Nano | Each 80 mg Nano curcumin capsule (curcumin 72%+ DMC 25%+ BDMC 3%) | 1 capsule per day | 8 weeks |
| Asan et al. [25] | Capsules | Each capsule contains 46.67 mg gel highly bioavailable formulation of curcumin equivalent to 950g dry extract of *Curcuma longa* L. | 2 capsules daily | 8 weeks |
| Asghari et al. [26] | Nano | CP Group: 80 mg of Nano-curcumin and placebo | 1 capsule of curcumin and 2 capsules of a placebo for omega 3 fatty acids daily | 12 weeks |
| Askari et al. [27] | Capsules | Each capsule contains 500mg curcumin + 5mg piperine | 2 capsules per day | 2 weeks |
| Atakan et al. [28] | Powder | 4 g of turmeric added to their main meals | 2g for lunch and 2g for dinner daily | 8 weeks |
| Barber-Chamoux et al. [29] | Capsules | 500mg curcumin capsule | 10 capsules per day | 2 hours |
| Bateni et al. [30] | Nano | 80mg curcumin in Nano-micelle form | 80mg/day | 12 weeks |
| Boshagh et al. [31] | Tablets | Each tablet contains 500mg curcumin + 5 mg piperine | 1 tablet per day | 12 weeks |
| Campbell et al. [32] | Pills | Each 500mg pill contains 193mg of curcuminoids in the form of curcumin, 15.3% DMC, and 2.8% BDMC infused into 60% soluble fiber from fenugreek | 1 pill per day | 12 weeks |
| Chashmniam et al. [33] | Capsules | Each capsule contains 250 mg, equivalent to 50 mg pure curcumin | 1 capsule daily | 8 weeks |
| Chuengsamarn et al. [34] | Capsules | 250 mg of Curcuminoids | 3 capsules per time (twice a day) | 9 months |
| Chuengsamarn et al. [35] | Capsules | Each curcumin capsule contains 250mg of curcuminoid content | 3 capsules per time (twice a day) | 6 months |
| Cicero et al. [36] | Tablets | Each tablet contains 800 mg phytosomal curcumin (200mg curcumin+ 120mg phosphatidylserine+ 480 mg | 2 tablets per day | 8 weeks |

| | | | | |
|---|---|---|---|---|
| | | phosphatidylcholine + 8mg piperine from *Piper Nigrum L.* dry extract) | | |
| Darmian et al. [37] | Capsules | Each capsule contains 700mg of turmeric powder | 3 capsules daily | 8 weeks |
| Dolati et al. [38] | Capsules | Curcumin group: 250 mg curcumin capsules | 2 capsules daily | 8 weeks |
| Dolati et al. [39] | Capsules | Cur Group: 500mg of curcumin | 500 mg per day | 8 weeks |
| Fergusona et al. [40] | Tablets | Curcumin only group: 25g/d fat spread +200 mg/d curcumin | 2 tablets of curcumin each day | 4 weeks |
| Funamoto et al. [41] | Capsules | Each capsule contains 30mg theracurmin | 3 capsules per time, twice daily | 24 weeks |
| Funamoto et al. [42] | Capsules | 30mg per capsule of Theracurmin | 3 capsules per day | 6 months |
| Funamoto et al. [43] | Capsules | Each Capsule contains 90 mg of curcumin | 2 capsules per day | 24 weeks |
| Garg MD et al. [44] | Capsules | The curcumin capsules contained 95% curcumin extracted from the rhizome of *Curcuma longa* (turmeric) | 4000mg of curcumin per day in 2 divided doses | 4 days |
| Ghaffari et al. [45] | Powder | Turmeric only group: 1000mg turmeric per time | 3 times per day | 12 weeks |
| Ghazimoradi et al. [46] | Capsules | 500mg of curcumin<br>500mg of phospholipidated curcumin | 1g per day, two capsules daily | 6 weeks |
| Hariri et al. [47] | Capsules | Each capsule contains 250mg phospholipidated curcumin (50mg curcumin) | 1 capsule daily | 8 weeks |
| Haroyan et al. [48] | Capsules | Curamed group: (500-mg of curcuminoid+49-52 mg volatile oil from *Curcuma longa* Linn Rhizome) capsules<br>Curamin group: 500-mg capsule (350 curcuminoids + 150 mg boswellic acid) | 1 capsule, three times per day | 12 weeks |
| Hellmann et al. [49] | Tablets | Each tablet contains Mervia 1000mg corresponding to 400 mg of curcuminoids | Twice daily | 6 weeks |
| Hellmann et al. [50] | Tablets | Group C: Each 1000 mg Meriva tablet contain 200 mg of curcumin and 1 prednisolone 50mg | Curcumin tablets twice daily + prednisolone 1 tablet per day | 11 days |
| Heshmati et al. [51] | Capsules | Each capsule contains 500mg of curcumin powder or maltodextrin | 3 capsules daily | 12 weeks |
| Heshmati et al. [52] | Capsules | Each capsule contains 500 mg of curcumin | 3 capsules per day | 12 weeks |
| Hondaei et al. [53] | Capsules | Each 500 mg capsule of curcumin contains 444 mg of curcuminoid and 38mg of turmeric oil | 3 capsules daily | 10 weeks |
| Ismail et al. [54] | Capsules | Each capsule contains 500mg curcumin | 1 capsule daily | 4 weeks |
| Ismail et al. [55] | Capsules | 500mg contains 95% of Curcuminoids | 500mg once daily | 4 weeks |
| Jamilian et al. [56] | Pills | 500 mg of curcumin | 500 mg/day curcumin | 12 weeks |
| Jarhahzaden et al. [57] | Capsules | Turmeric capsules | 2 g daily | 8 weeks |

| | | | | |
|---|---|---|---|---|
| Javandoosi et al. [58] | Capsules | 500 mg of curcumin<br>500 mg of curcumin Phospholipid complex | Twice a day | 6 weeks |
| Jazayeri-Tehrani et al. [59] | Nano | 40 mg Nano-curcumin capsules | 2 capsules daily | 3 months |
| Jimenez-Osorio et al. [60] | Capsules | 1 Capsule containing curcumin 107mg | 1 capsule each meal (total 320 mg/day) | 8 weeks |
| Karandish et al. [61] | curcumin Capsules | Curcumin Group: Curcumin supplement containing 500mg of the turmeric extracts (at least 95 % curcuminoids) with the placebo for zinc | 1 tablet of placebo for zinc before breakfast & 1 capsule of curcumin after breakfast daily | 3 months |
| Karandish et al. [62] | Capsules | 500 mg-BCM95/ Curcugreen | 1 capsule per day | 3 months |
| Kelardeh et al. [63] | Nano | Curcumin group : Curcumin 80 mg as Nano-micelle | 1 capsule per day | 12 weeks |
| Khajehdehi et al. [64] | Capsules | Each capsule contains 500 mg turmeric with 22.1 mg was the active ingredient curcumin | 3 capsules daily with each meal | 2 months |
| Khajehdehi et al. [65] | Capsules | Each capsule contains 500 mg turmeric (including 22.1 mg of active ingredient curcumin) | 3 capsules daily with each meal | 3 months |
| Kisiolek et al. [66] | Capsules | 1000mg of Longvida Optimized Curcumin | 1000mg per day | 2 weeks |
| Kocher et al. [67] | Capsules | 4 capsules equivalent to 80.4mg curcumin + 15.6mg DMC + 2mg BDMC | 4 capsules x Three times per day | 6 weeks |
| Krishnareddy et al. [68] | Capsules | Each CGM capsule 250mg contains 39.1% curcuminoid | 1 capsule daily | 8 weeks |
| Majeed et al. [69] | Capsules | Each capsule contains 25 mg of Calebin A (Curcousin) + 3mg of piperine (BioPerine) | 2 capsules daily | 3 months |
| Mamsharifi et al. [70] | Nano | 80mg of Nano Curcumin | 80 mg daily | 3 months |
| Mankowski et al. [71] | Capsules | Each capsule contains 500 mg of curcumin + DMC + BDMC | Twice daily | 12 weeks |
| Mirhafez et al. [72] | Capsules | Each phospholipidated curcumin capsule contains 250 mg/day, equivalent to 50 mg/day pure curcumin | 1 capsule per day | 8 weeks |
| Mirhafez et al. [73] | Capsules | Meriva, 250mg phospholipidated curcumin equivalent to 50mg curcumin | 1 capsule per day | 8 weeks |
| Mirhafez et al. [74] | Capsules | 500 mg of complexTM plus BioperineTM 5mg | 500mg per day | 8 weeks |
| Mirhafez et al. [75] | Capsules | Each 250mg of phospholipidated curcumin capsule contains 50mg of pure curcumin | 250mg per day | 2 months |
| Mirzabeigi et al. [76] | Capsules | 500mg of curcumin | 1 capsule four times per day | 2 months |
| Mohammadi et al. [77] | Capsules | Curcumin phospholipid complex Group: Each 500 mg capsule (Absorption-enhanced curcumin containing 200mg pure curcumin)<br>Curcumin Group: Unformulated curcumin | 1 capsule 500 mg twice a day (Total 1g/day) | 6 weeks |

| | | | | |
|---|---|---|---|---|
| Mohammadi et al. [78] | Powder | Curcumin-only group: One sachet contains 1 g of curcumin + low calorie diet | 1 sachet per day along with calorie diet | 8 weeks |
| Mokhtari et al. [79] | Nano | 80 mg of Nano-curcumin | 80 mg/day | 12 weeks |
| Na et al. [80] | Capsules | Each capsule contains 150 mg curcuminoids | 150mg capsule twice daily | 3 months |
| Neta et al. [81] | Capsules | 500mg of *Curcuma longa L*. + 5 mg of piperine | Once a day | 4 months |
| Nowak et al. [82] | Powder | Curcumin supplementation powder form mixed with food | 25 mg/kg body weight per day | 12 months |
| Osali [83] | Capsules | Curcumin-only group: Each capsule contains 80mg Nano-curcumin supplement | 80mg/day | 6 weeks |
| Panahi et al. [84] | Capsules | Each capsule contains 500 mg of curcuminoid + 5 mg of Bioperine | Twice a day | 8 weeks |
| Panahi et al. [85] | Capsules | Each capsule contains 500 mg curcuminoid (curcumin+DMC+BDMC)+ 5mg piperine | 2 capsules daily | 8 weeks |
| Panahi et al. [86] | Capsules | Each capsule contains 500 mg curcuminoids + 5 mg bioperine | 3 dose daily | 6 weeks |
| Panahi et al. [87] | Capsules | Each capsule contains 5 mg piperine + 500 mg curcuminoid | 2 capsules (daily) | 8 weeks |
| Panahi et al. [88] | Capsules | 500mg Capsule contain 20%curcumin + soy phosphatidylcholine + microcrystalline | 2 capsules per day | 8 weeks |
| Panahi et al. [89] | Capsules | One capsule contains 500mg of curcumin +5mg of piperine | 2 capsules per day | 8 weeks |
| Panahi et al. [90] | Capsules | Each capsule: 5mg of piperine added to 500mg of curcuminoids | 2 capsules per day | 12 weeks |
| Panahi et al. [91] | Capsules | 500mg phytosomal curcumin capsule | 1000mg/day in 2 divided doses | 8 weeks |
| Panahi et al. [92] | Capsules | Each 500mg capsule contains Curcuminoides+ 5mg piperine | 1 capsule daily | 3 months |
| Pashine et al. [93] | Capsules | 1.4 gm per day aqueous extract of Turmeric | 1.4gm per day in two divided dose | 3 months |
| Pierro et al. [94] | Capsules | Each capsule contains 800 mg/dose/die of *Curcuma longa* extract (95% curcumin) complexed with sunflower phospholipid and blended with 8 mg/dose/die of piperine | 1 dose daily | 1 month |
| Porasgari et al. [95] | Capsules | Curcumin group: Each capsule contains 450 mg of turmeric rhizome powder + 50 mg of turmeric extract (including 47.5 mg of curcumin) | 1 capsule daily | 8 weeks |
| Rahimi et al. [96] | Nano | Nano-curcumin as Nano-micelle 80mg | 80mg/day | 3 months |
| Rahmani et al. [97] | Capsules | Each capsule contains 500mg of an amorphous dispersion preparation comprising 70mg curcuminoids | 1 capsule per day | 8 weeks |
| Reis et al. [98] | Capsules | 500 mg of Curcumin | 1 capsule daily | 3 months |
| Rezaei et al. [99] | Nano | Each Nano-curcumin capsule contains curcuminoid + polysorbate surfactant + ascorbic acid + vitamin E + natural oils + distilled $H_20$. | 80mg/day | 12 weeks |

| | | | | |
|---|---|---|---|---|
| Saadati et al. [100] | Capsules | Each capsule contains 500 mg curcumin | 3 capsules daily | 12 weeks |
| Saadati et al. [101] | Capsules | Each 500mg curcumin capsule contains Biocurcumin - 95(95%curcuminoids + essential oil of turmeric-ar-turmerone) | 3 capsules per day | 12 weeks |
| Saberi-Karimian et al. [102] | Capsules | Curcumin Phospholipid Complex Group: Each absorption-enhanced curcumin 500 mg capsule contained 100mg pure curcumin | 500 mg capsule, twice per day | 6 weeks |
| Saberi-Karimian et al. [103] | Capsules | Curcumin Group: Unformulated curcumin capsules<br>Simple formula curcumin 200mg<br>Modified formula curcumin contained 200mg pure curcumin | 1 g per day | 6 weeks |
| Saberi-Karimian et al. [104] | Capsules | 500 mg curcuminoids plus 5 mg piperine | 1 capsule per day | 8 weeks |
| Sadeghzadeh et al. [105] | Nano | Curcumin Group: Each capsule contains 80 mg of Nano-curcumin | 1 dose per day | 6 months |
| Sangouni et al. [106] | Capsules | Curcumin powder 200mg + placebo | 1 capsule daily | 12 weeks |
| Saraf-Bank et al. [107] | Tablets | 500 mg tablet containing standardized 95% turmeric extract | 1 tablet per day | 10 weeks |
| Saraf-Bank et al. [108] | Tablets | 500 mg tablet containing standardized 95% turmeric extract | 1 tablet per day | 10 weeks |
| Sedighiyan et al. [109] | Nano | Each capsule contains 40mg of Nano-curcumin | 2 capsules per day | 2 months |
| Shafabakhsh et al. [110] | Tablets | Curcumin | 1000mg/day | 12 weeks |
| Shirmohammadi et al. [111] | Capsules | Each capsule contains 500mg of the curcumin-phosphatidylcholine complex | 2 capsules daily | 6 weeks |
| Sohaei et al. [112] | Pellets | 500 mg of Curcumin Supplement contained standardized turmeric extract 95% in form of pellets (475 mg curcuminoids covering 70–80% curcumin, 15–20% DMC and 2.5–6.5% BDMC) | 500mg × twice a day | 6 weeks |
| Soltani et al. [113] | Nano | Nano-curcumin contains curcuminoid mixture (79%curcumin + 17.6%(DMC) + 3% (BDMC)) +polysorbate + vitamin E + vitamin C+ natural oil | 80mg/day | 12 weeks |
| Srinivasan et al. [114] | Capsules | *Curcuma longa* 400mg | 3 times per day | 3 months |
| Tamaddoni et al. [115] | Capsules | Each capsule contains 500 mg curcuminoids = 450 mg curcumin + 30 mg DMC and 20 mg BDMC | 2 capsules daily | 12 weeks |
| Thota et al. [116] | Tablets | Each 500 mg tablet contains 90 mg of curcumin | 2 tablets per day | 12 weeks |
| Uchio et al. [117] | Tablets | Each 900mg tablet of hot water extract of *Curcuma longa L.* (WEC) equivalent to 400μg bisacurone, 80μg turmeronnol A and 20μg turmeronnol B | 3 tablets daily | 12 weeks |
| Yaikwawong et al. [118] | Capsules | Each curcumin capsule contains 250 mg of curcuminoids | 3 capsules per time, twice per day | 12 months |
| Yaikwawong et al. [119] | Capsules | Each curcumin capsule contains 250 mg of curcuminoids | 3 capsules per time, twice per day | 12 months |

| | | | | |
|---|---|---|---|---|
| Yang et al. [120] | Capsules | Each capsule contains 630mg of curcumin extract | Thrice a day | 12 weeks |
| Zohrabi et al. [121] | Capsules | Curcumin Group: Each capsule 500mg contains 475 mg of curcuminoids along with natural turmeric essential oil and a standard diet | Twice daily | 12 weeks |

**Supplementary Table S4**

**Summary of outcomes related to parameters associated with metabolic syndrome in the treatment group compared to the control group that highlights the effects of Curcumin Supplementation in the Controlled Clinical Studies**

| First Author, Year | BMI | WC | W/H | FPS | INS | HbA1c | GLU | FBS | PPBS | OGTT | HOMA-IR | HOMA-β | QUICKI | TG | TC | LDL | VLDL | HDL | SBP | DBP |
|---|---|---|---|---|---|---|---|---|---|---|---|---|---|---|---|---|---|---|---|---|
| Abed & Abdulridha [18] | - | - | - | - | - | - | - | - | - | - | - | - | - | - | - | - | - | - | ↓ | ↓ |
| Adibian et al. [19] | - | - | - | - | - | - | - | - | - | - | - | - | - | ↓ | ↓ | ↓ | - | ↓ | - | - |
| Afshar et al. [20] | ↓ | - | - | - | - | - | - | ↑ | - | - | - | - | - | ↓ | ↓ | ↓ | - | ↓ | - | - |
| Alizadeh et al. [21] | ↓ | - | - | - | - | - | - | - | - | - | - | - | - | - | - | - | - | - | - | - |
| Alvarenga et al. [22] | ↑ | ↑ | - | - | - | ↑ | ↑ | - | - | - | - | - | - | ↓ | ↓ | ↓ | - | ↑ | - | - |
| Alvarenga et al. [23] | ↑ | ↓ | - | - | - | ↓ | - | ↓ | ↓ | - | - | - | - | - | - | - | - | - | - | - |
| Asadi et al. [24] | ↓ | ↓ | - | - | ↓ | - | - | ↓ | - | - | ↓ | - | - | ↓ | ↓ | ↓ | - | ↓ | - | - |
| Asan et al. [25] | - | - | - | - | ↓ | ↓ | - | ↓ | - | - | ↓ | - | ↔ | ↓ | ↓ | ↓ | - | ↔ | - | - |
| Asghari et al. [26] | - | - | - | - | - | - | - | ↑ | - | - | - | - | - | ↔ | ↓ | ↓ | ↓ | ↑ | - | - |
| Askari et al. [27] | ↓ | ↓ | ↑ | - | - | - | - | ↓ | - | - | - | - | - | ↓ | ↓ | ↓ | - | ↑ | - | - |
| Atakan et al. [28] | - | - | - | - | - | - | - | - | - | - | - | - | - | - | - | - | - | - | ↓ | ↑ |
| Barber-Chamoux et al. [29] | ↑ | ↓ | - | - | ↑ | ↔ | - | ↑ | - | - | ↑ | ↑ | - | ↓ | ↓ | ↓ | - | ↑ | ↓ | ↓ |
| Bateni et al. [30] | ↓ | ↓ | - | - | - | - | - | - | - | - | - | - | - | ↓ | ↓ | ↓ | - | ↑ | ↓ | ↓ |
| Boshagh et al. [31] | - | - | - | - | ↑ | - | ↑ | - | - | - | - | - | - | - | - | ↑ | - | ↑ | - | - |
| Campbell et al. [32] | ↑ | - | - | - | - | - | - | ↑ | - | - | - | - | - | ↑ | ↑ | ↑ | - | ↓ | - | - |
| Chashmniam et al. [33] | - | ↓ | - | - | ↓ | ↓ | - | ↓ | - | ↓ | ↓ | ↑ | - | - | - | - | - | - | - | - |
| Chuengsamarn et al. [34] | - | ↓ | - | - | - | - | - | - | - | - | ↓ | - | - | ↓ | - | - | - | - | - | - |
| Chuengsamarn et al. [35] | ↓ | ↓ | - | ↓ | - | - | - | ↓ | - | - | ↓ | - | - | ↓ | ↓ | ↓ | - | ↑ | ↓ | ↑ |
| Cicero et al. [36] | - | ↓ | - | - | - | - | - | ↓ | - | - | - | - | - | ↓ | - | - | - | ↑ | ↓ | ↓ |
| Darmian et al. [37] | ↓ | ↑ | ↑ | - | ↓ | - | - | ↓ | - | - | ↓ | - | ↑ | ↑ | ↑ | ↑ | - | ↑ | - | - |
| Dolati et al. [38] | ↓ | - | - | - | - | - | - | - | - | - | ↓ | - | - | - | - | - | - | - | - | - |
| Dolati et al. [39] | - | - | - | - | - | - | ↑ | - | - | - | - | - | - | ↑ | ↑ | ↓ | - | ↑ | - | - |
| Fergusona et al. [40] | ↑ | - | - | - | - | ↓ | ↓ | - | - | - | - | - | - | ↑ | - | ↓ | - | ↑ | ↑ | ↓ |
| Funamoto et al. [41] | - | - | - | - | - | - | ↔ | - | - | - | - | - | - | - | - | - | - | - | - | - |
| Funamoto et al. [42] | ↓ | ↓ | ↔ | - | - | - | - | - | - | - | - | - | - | ↓ | ↓ | ↓ | - | ↑ | - | - |
| Funamoto et al. [43] | ↑ | ↑ | ↑ | - | - | - | - | - | - | - | - | - | - | - | - | - | - | - | - | - |
| Garg MD et al. [44] | ↑ | ↓ | - | ↓ | - | - | - | ↓ | - | - | ↓ | - | ↔ | - | - | - | - | - | - | - |
| Ghaffari et al. [45] | ↑ | ↑ | - | - | ↓ | ↓ | - | ↑ | - | - | ↓ | ↓ | - | - | - | - | - | - | ↔ | ↑ |
| Ghazimoradi et al. [46] | - | ↓ | - | - | - | - | - | - | - | - | ↓ | - | - | - | - | - | - | - | - | - |
| Hariri et al. [47] | - | - | - | - | ↓ | - | - | ↑ | - | - | - | - | - | - | - | - | - | - | - | - |
| Haroyan et al. [48] | - | - | - | - | ↓ | - | - | ↓ | - | - | ↔ | - | ↑ | ↑ | ↓ | ↓ | ↔ | ↓ | - | - |

| | | | | | | | | | | | | | | | | | | | | |
|---|---|---|---|---|---|---|---|---|---|---|---|---|---|---|---|---|---|---|---|---|
| Hellmann et al. [49] | - | - | - | - | - | - | - | ↑ | - | - | - | - | - | ↓ | ↑ | ↑ | ↓ | ↓ | - | - |
| Hellmann et al. [50] | - | - | - | - | - | - | - | ↓ | - | - | - | - | - | ↓ | ↑ | ↑ | - | ↑ | - | - |
| Heshmati et al. [51] | ↓ | ↓ | - | ↓ | - | ↓ | - | ↓ | - | - | ↓ | - | ↑ | ↓ | ↓ | ↓ | - | ↑ | ↓ | ↓ |
| Heshmati et al. [52] | ↑ | - | - | - | - | - | ↓ | - | - | - | - | - | - | ↓ | ↓ | - | - | - | ↑ | ↑ |
| Hondaei et al. [53] | ↓ | - | - | - | ↓ | - | - | ↓ | - | - | - | - | - | - | - | - | - | - | - | - |
| Ismail et al. [54] | - | - | - | - | - | - | - | - | - | - | - | - | - | ↓ | ↓ | ↓ | - | ↑ | - | - |
| Ismail et al. [55] | ↑ | - | - | - | - | - | - | - | - | - | - | - | - | - | - | - | - | - | - | - |
| Jamilian et al. [56] | - | - | - | - | - | - | - | ↑ | ↑ | - | - | - | - | ↑ | ↑ | ↑ | - | ↑ | ↑ | ↓ |
| Jarhahzaden et al. [57] | - | - | - | - | - | - | - | - | - | - | - | - | - | - | - | - | - | - | ↑ | ↑ |
| Javandoosi et al. [58] | ↔ | - | - | - | ↑ | - | - | ↓ | - | - | ↑ | - | - | ↓ | ↓ | ↓ | - | ↑ | - | - |
| Jazayeri-Tehrani et al. [59] | ↑ | - | - | - | - | - | - | - | - | - | - | - | - | ↓ | ↓ | ↓ | - | ↑ | ↓ | ↓ |
| Jimenez-Osorio et al. [60] | ↓ | ↓ | - | - | - | ↑ | - | ↑ | - | - | ↓ | ↑ | - | ↔ | ↓ | ↓ | ↑ | ↑ | ↑ | ↓ |
| Karandish et al. [61] | - | - | - | - | ↔ | - | - | ↓ | - | - | ↔ | - | - | ↑ | ↔ | ↔ | ↑ | ↓ | - | - |
| Karandish et al. [62] | - | - | - | - | - | - | ↓ | - | - | - | - | - | - | - | - | - | - | - | - | - |
| Kelardeh et al. [63] | ↑ | - | - | - | - | - | - | ↓ | - | - | - | - | - | ↓ | ↑ | ↑ | - | ↓ | - | - |
| Khajehdehi et al. [64] | ↓ | ↓ | ↓ | - | - | - | - | - | - | - | - | - | - | - | - | - | - | - | - | - |
| Khajehdehi et al. [65] | ↑ | - | ↔ | - | - | - | - | ↓ | - | - | - | - | - | ↑ | ↑ | ↑ | - | ↓ | ↓ | ↓ |
| Kisiolek et al. [66] | - | - | - | - | - | - | - | ↑ | - | - | - | - | - | ↑ | ↑ | ↑ | ↑ | ↓ | - | - |
| Kocher et al. [67] | ↓ | R | - | - | - | - | - | - | - | - | - | - | - | - | - | - | - | - | - | - |
| Krishnareddy et al. [68] | - | - | - | - | - | - | - | - | - | - | - | - | - | ↑ | ↑ | ↔ | - | ↑ | - | - |
| Majeed et al. [69] | ↓ | - | - | - | ↓ | ↑ | - | ↓ | - | - | ↓ | - | ↑ | ↓ | ↓ | ↓ | ↓ | ↑ | - | - |
| Mamsharifi et al. [70] | ↓ | - | - | - | - | ↓ | ↓ | - | - | - | ↓ | - | - | ↓ | ↓ | ↓ | - | ↑ | - | - |
| Mankowski et al. [71] | ↑ | - | - | - | ↑ | ↑ | - | ↑ | - | - | ↑ | ↓ | - | ↑ | - | ↓ | - | ↑ | ↓ | ↓ |
| Mirhafez et al. [72] | ↑ | ↓ | - | - | - | - | ↓ | - | - | - | - | - | - | ↓ | - | - | - | ↑ | ↓ | - |
| Mirhafez et al. [73] | - | - | - | - | - | - | - | - | - | - | - | - | - | ↓ | ↑ | ↑ | - | ↑ | - | - |
| Mirhafez et al. [74] | - | - | - | - | - | ↑ | - | ↓ | - | - | - | - | - | - | - | - | - | - | ↓ | ↓ |
| Mirhafez et al. [75] | ↓ | - | - | - | - | - | - | - | - | - | - | - | - | - | - | - | - | - | - | - |
| Mirzabeigi et al. [76] | - | - | - | - | ↓ | ↑ | - | ↑ | - | - | ↑ | ↑ | ↔ | ↓ | ↓ | ↓ | - | ↓ | - | - |
| Mohammadi et al. [77] | ↓ | - | - | - | - | - | - | - | - | - | - | - | - | ↑ | ↓ | ↓ | - | ↑ | - | - |
| Mohammadi et al. [78] | ↓ | ↓ | - | - | - | - | - | - | - | - | - | - | - | - | - | - | - | - | ↓ | ↓ |
| Mokhtari et al. [79] | ↓ | - | - | - | ↓ | ↓ | ↓ | - | - | - | ↑ | ↑ | - | - | - | - | - | - | - | - |
| Na et al. [80] | - | - | - | - | - | - | - | - | - | - | - | - | - | ↓ | ↓ | ↓ | ↓ | ↑ | - | - |
| Neta etal. [81] | ↓ | ↓ | - | - | - | - | - | - | - | - | - | - | - | - | - | - | - | - | - | - |
| Nowak et al. [82] | ↑ | - | ↑ | - | - | - | - | - | - | - | - | - | - | ↑ | ↔ | ↓ | - | ↓ | - | - |
| Osali [83] | ↓ | - | - | - | - | ↓ | - | ↓ | - | - | - | - | - | ↑ | ↑ | ↑ | - | ↑ | - | - |
| Panahi et al. [84] | ↓ | - | - | - | - | ↓ | - | ↓ | - | - | - | - | - | ↑ | ↓ | ↓ | - | ↓ | - | - |
| Panahi et al. [85] | ↓ | ↑ | - | - | - | - | - | - | - | - | - | - | - | - | ↓ | ↓ | - | - | - | - |

| | | | | | | | | | | | | | | | | | | | | |
|---|---|---|---|---|---|---|---|---|---|---|---|---|---|---|---|---|---|---|---|---|
| Panahi et al. [86] | - | - | - | - | - | - | - | - | - | - | - | - | - | ↑ | ↓ | ↓ | - | ↔ | - | - |
| Panahi et al. [87] | ↑ | ↓ | ↓ | - | ↓ | - | ↓ | - | - | - | ↓ | - | ↔ | ↓ | ↓ | ↓ | - | ↓ | - | - |
| Panahi et al. [88] | - | - | - | - | ↓ | - | - | ↔ | - | - | ↔ | - | - | ↑ | ↓ | ↑ | - | - | - | - |
| Panahi et al. [89] | ↓ | ↓ | - | - | - | - | - | ↔ | - | - | - | - | - | ↓ | ↓ | ↓ | - | ↑ | ↓ | ↓ |
| Panahi et al. [90] | ↑ | ↑ | ↓ | - | ↑ | - | - | ↓ | - | - | ↑ | - | - | ↓ | ↓ | ↓ | - | ↔ | ↑ | ↑ |
| Panahi et al. [91] | ↑ | - | - | - | - | - | - | - | - | - | - | - | - | - | - | - | - | - | - | - |
| Panahi et al. [92] | ↓ | ↑ | - | - | - | - | - | - | - | - | - | - | - | - | - | - | - | - | - | - |
| Pashine et al. [93] | - | - | - | - | ↓ | - | - | ↑ | - | - | ↓ | - | ↑ | ↑ | ↑ | ↑ | - | ↓ | - | - |
| Pierro et al. [94] | - | - | - | - | ↑ | ↔ | - | ↑ | - | - | ↑ | - | - | - | - | - | - | - | ↓ | ↓ |
| Porasgari et al. [95] | - | - | - | - | - | - | - | - | - | - | - | - | - | - | - | - | - | - | ↓ | ↑ |
| Rahimi et al. [96] | ↓ | - | - | - | ↓ | - | - | - | - | - | ↓ | - | - | ↓ | ↑ | ↑ | - | ↑ | - | - |
| Rahmani et al. [97] | ↓ | - | - | - | - | ↓ | ↓ | - | - | - | ↓ | - | - | - | - | - | - | - | - | - |
| Reis et al. [98] | - | ↓ | - | - | - | ↓ | - | ↓ | - | - | ↓ | - | - | - | - | ↓ | - | - | - | - |
| Rezaei et al. [99] | ↑ | - | - | - | - | ↓ | - | ↓ | - | - | - | - | - | ↑ | ↑ | ↑ | ↓ | ↑ | - | - |
| Saadati et al. [100] | ↓ | - | ↓ | - | ↓ | - | - | ↓ | - | - | ↓ | - | ↑ | - | - | - | - | - | - | - |

↑=Value increase in the treatment group compared to control group, ↓=Value decrease in the treatment group compared to control group, ↔ = Value approximately or exactly equal between treatment group and control group, BMI=Body Mass Index, WC=Wrist circumstance, W/H=Waist/Hip ratio, FPS=Fasting Plasma Insulin, Ins=Insulin, HbA1c=Glycated Hemoglobin, Glu=Blood Glucose, FBS=Fasting blood sugar, PPBS=2Hour Post prandial blood sugar, OGTT=Oral Glucose Tolerance Test, HOMA-IR=Homeostatic Model Assessment of Insulin Resistance, HOMA-β=Homeostatic Model Assessment of Beta-cell Function, QUICKI=Quantitative Insulin Sensitivity Check Index, TG=Triglyceride, TC=Total Cholesterol, LDL=Low density lipoprotein, VLDL=Very low density lipoprotein, HDL=High density lipoprotein, SBP=Systolic blood pressure, DBP=Diastolic Blood Pressure.

**Supplementary Table S5**

**Evaluating the effects of Curcumin consumption on Body Mass Index (BMI), Waist Circumference (WC), Waist–Hip Ratio (W/H Ratio) HOMA-IR, HOMA-β, and QUICKI in clinical studies of metabolic syndrome.**

| Author, Year, Intervention | BMI (kg/m²) | WC (cm) | W/H Ratio | HOMA-IR | HOMA- β | QUICKI |
|---|---|---|---|---|---|---|
| Afshar et al. [20] | | | | | | |
| Treatment | 25.95±4.97 | | | | | |
| Placebo | 27.11±5.04 | | | | | |
| Alizadeh et al. [21] | | | | | | |
| Treatment | 26.19±3 | | | | | |
| Placebo | 26.65±3.06 | | | | | |
| Alvarenga et al. [22] | | | | | | |
| Treatment | 27±4.1 | 93.3±12.5 | | | | |
| Placebo | 26.3±2.9 | 92.9±8.4 | | | | |
| Asadi et al. [24] | | | | | | |
| Treatment | 31.01±4.1 | 101.1±9.5 | | | | |
| Placebo | 30.8±3.7 | 103.5±9.3 | | | | |
| Asan et al. [25] | | | | | | |
| Treatment | 28.1±6.7 | 85.5±13.2 | | 1.6±1.9 | | |
| Placebo | 29.2±4.3 | 89.9±10.6 | | 1.7±1.1 | | |
| Asghari et al. [26] | | | | | | |
| Curcumin group | | | | 6.22±2.96 | | 0.129±0.008 |
| Placebo | | | | 6.69±2.95 | | 0.128±0.008 |
| Atakan et al. [28] | | | | | | |
| Treatment | 29.38±5.41 | 82.13±13.51 | 0.87±0.14 | | | |
| Placebo | 29.88±5.04 | 83.67±14.62 | 0.85±0.17 | | | |
| Bateni et al. [30] | | | | | | |
| Treatment | 29.8±4.5 | 102.4±10.9 | | 3.1±1.9 | 54.4±30.9 | |
| Placebo | 29.1±4.1 | 104.1±8.4 | | 2.6±2.7 | 35.6±26.6 | |
| Boshagh et al. [31] | | | | | | |
| Treatment | 26.82±4.89 | 89.8±12.97 | | | | |
| Placebo | 27.1±4.25 | 92.81±11.2 | | | | |
| Chashmniam et al. [33] | | | | | | |
| Treatment | 29.6±3.45 | | | | | |
| Placebo | 27.72±4.25 | | | | | |
| Chuengsmarn et al. [34] | | | | | | |
| Treatment | | 84.4±9.95 | | 3.22±2.11 | 58.54±52.4% | |
| Placebo | | 91.6±15.72 | | 4.08±3.24 | 48.78±47.2% | |
| Chuengsmarn et al. [35] | | | | | | |
| Treatment | | 88.2±11.94 | | 2.75±1.99 | | |
| Placebo | | 90.4±11.14 | | 5.66±2.59 | | |
| Cicero et al. [36] | | | | | | |
| Treatment | 26.3±1.4 | 89±4 | | 3.8±1.1 | | |
| Placebo | 26.4±1.7 | 90±9 | | 4.7±1.4 | | |
| Darmian et al. [37] | | | | | | |
| Turmeric group | | 95.22±1.54 | | | | |
| Placebo | | 98.23±2.17 | | | | |
| Dolati et al. [38] | | | | | | |
| Curcumin group | 26.59±2.06 | 85.35±5.56 | 0.82±0.06 | 1.69±0.65 | | 0.36±0.02 |
| Placebo | 26.89±1.56 | 81.85±4 | 0.77±0.01 | 1.98±1.14 | | 0.35±0.02 |
| Dolati et al. [39] | | | | | | |
| Curcumin group | 26.51±1.8 | | | 1.7±0.6 | | |
| Placebo | 26.8±1.5 | | | 2.04±1.1 | | |
| Funamoto et al. [42] | | | | | | |
| Treatment | 25±4.5 | | | | | |
| Placebo | 24.8±2.7 | | | | | |

| | | | | | | |
|---|---|---|---|---|---|---|
| Ghaffari et al. [45] | | | | | | |
| Turmeric only | 31.5±4.73 | 101±11.3 | 0.93±0.05 | | | |
| Placebo | 32.8±4.94 | 105±10 | 0.93±0.45 | | | |
| Hariri et al. [47] | | | | | | |
| Treatment | 29.83±6.03 | 100.99±14.68 | 0.96±0.07 | | | |
| Placebo | 28.75±3.47 | 97.13±9.08 | 0.95±0.06 | | | |
| Heshmati et al. [51] | | | | | | |
| Treatment | 27.71±5.01 | 88.92±11.78 | | 3.03±1.87 | | 0.33±0.04 |
| Placebo | 26.93±4.03 | 90.76±11.74 | | 3.15±2.6 | | 0.33±0.04 |
| Hondaei et al. [53] | | | | | | |
| Treatment | 28.9±3.73 | 100±8.92 | | 62.4±42 | | |
| Placebo | 28.1±2.5 | 96±8.09 | | 65±44 | | |
| Ismail et al. [54] | | | | | | |
| Treatment | | 92.77 ± 8.85 | | 2.36± 1.49 | 134±98 | |
| Placebo | | 97.95 ± 6.30 | | 3.05± 1.01 | 140±66 | |
| Jamilian et al. [56] | | | | | | |
| Treatment | | | | 2.4±0.8 | | 0.338±0.017 |
| Placebo | | | | 2.4±0.5 | | 0.335±0.012 |
| Jazayeri-Tehrani et al. [59] | | | | | | |
| Treatment | 29.7±2.1 | 99.6±5.7 | | 1.39±0.21 | | 0.3643±0.0092 |
| Placebo | 29.9±2.53 | 102.5±6.9 | | 1.65±0.18 | | 0.3543±0.0068 |
| Jimenez-Osorio et al. [60] | | | | | | |
| Treatment | 29.9±6.35 | | | | | |
| Placebo | 26.8±8.63 | | | | | |
| Karandish et al. [61] | | | | | | |
| Curcumin group | 28.78± 2.86 | | | | | |
| Placebo | 30.05± 2.65 | | | | | |
| Kelardeh et al. [63] | | | | | | |
| Curcumin group | 27.48±1.3 | | | | | |
| Placebo | 27.21±1.29 | | | | | |
| Kocher et al. [67] | | | | | | |
| Treatment | 26.9±4.7 | | | 2±1.2 | | |
| Placebo | 26.9±4.8 | | | 1.9±0.8 | | |
| Krishnareddy et al. [68] | | | | | | |
| Treatment | 30.8± 3.5 | | | | | |
| Placebo | 29.88± 4.5 | | | | | |
| Majeed et al. [69] | | | | | | |
| Treatment | 29.44±2.3 | 92.82±10.09 | | 3.88** | 60.74** | |
| Placebo | 30.79±1.91 | 98.53±10.23 | | 4.30** | 40.79** | |
| Mamsharifi et al. [70] | | | | | | |
| Treatment | | | | 1.7±0.5 | | |
| Placebo | | | | 1.71±0.37 | | |
| Mirhafez et al. [72] | | | | | | |
| Treatment | 29.75±5.84 | | | | | |
| Placebo | 28.78±4.22 | | | | | |
| Mirhafez et al. [73] | | | | | | |
| Treatment | 28±9.13 | 98±15.1 | 0.96±0.17 | | | |
| Placebo | 29.97±6.72 | 102±8.17 | 0.97±0.07 | | | |
| Mirhafez et al. [74] | | | | | | |
| Treatment | 29.1±3.8 | | 0.9±0.1 | | | |
| Placebo | 28.6±3.8 | | 0.9±0.1 | | | |
| Mohammadi et al. [77] | | | | | | |
| Cu-P complex | 31.03±5.11 | 100.8±11.57 | | | | |
| Curcumin | 30.36±3.8 | 97.01±11.14 | | | | |
| Placebo | 31.3±4.87 | 99.42±11.86 | | | | |
| Mokhtari et al. [79] | | | | | | |
| Treatment | 27.3±4.7 | | | 5.3±3.3 | | 0.31±0.02 |

| | | | | | | |
|---|---|---|---|---|---|---|
| Placebo | 29.9±5.2 | | | 5.8±2 | | 0.29±0.01 |
| Na et al. [80] | | | | | | |
| Treatment | 26.32±2.82 | | | 4.14± 1.81 | | |
| Placebo | 26.77±3.73 | | | 5.49± 2.15 | | |
| Neta et al. [81] | | | | | | |
| Treatment | 29.5±4.6 | | | 5.5±5 | 66.4±77.5 | |
| Placebo | 28.6±4.8 | | | 3.9±4.1 | 77.2±126.1 | |
| Osali [83] | | | | | | |
| Curcumin group | 29.66±2.6 | 103.9±6.87 | | | | |
| Placebo | 29.11±1.51 | 104.2±6.47 | | | | |
| Panahi et al. [87] | | | | | | |
| Treatment | 26.04±2.35 | | | 3±1.15 | 119.36±30.77 | 0.33±0.02 |
| Placebo | 27.57±1.63 | | | 2.59±0.74 | 109.62±34.07 | 0.33±0.01 |
| Panahi et al. [90] | | | | | | |
| Treatment | 27.98±3.05 | 95.33±10.77 | | | | |
| Placebo | 28.92±3.69 | 101.66±11.25 | | | | |
| Panahi et al. [92] | | | | | | |
| Treatment | 26±2 | | | 7.4±2 | 85±45.8 | |
| Placebo | 28±1 | | | 3.1±0.4 | 74.95±24.04 | |
| Pierro et al. [94] | | | | | | |
| Treatment | 26.2 ± 4.2** | 95.4 ± 11.8** | | | | |
| Placebo | 28.7 ± 3.4** | 99.5 ± 13.2** | | | | |
| Porasgari et al. [95] | | | | | | |
| Treatment | 38.87±5.43 | | 0.92±0.037 | | | |
| Placebo | 29.03±4.65 | | 0.88±0.036 | | | |
| Rahimi et al. [96] | | | | | | |
| Treatment | 25.57±2.71 | | | | | |
| Placebo | 27.5±3.38 | | | | | |
| Rahmani et al. [97] | | | | | | |
| Treatment | 30.11±4.39 | | | | | |
| Placebo | 31.37±5.33 | | | | | |
| Reis et al. [98] | | | | | | |
| Treatment | 30.35±5.81 | 96.85±14.99 | | | | |
| Placebo | 30.7±4.81 | 93.67±6.9 | | | | |
| Saadati et al. [100] | | | | | | |
| Treatment | 31.44±5.02 | 96.93±9.29 | 0.88±0.06 | 2.76±1.24 | | 0.2±0.008 |
| Placebo | 31.33±6.08 | 99.5±11.39 | 0.92±0.06 | 2.99±1.57 | | 0.2±0.009 |
| Sadeghzadeh et al. [105] | | | | | | |
| Curcumin group | | | | 0.78±0.46 | | |
| Placebo | | | | 0.77±0.48 | | |
| Sangouni et al. [106] | | | | | | |
| Curcumin + Placebo | 29.4±4.2 | 100.2±8.5 | | | | |
| Placebo | 29.7±4.7 | 101.4±7.4 | | | | |
| Shafabakhsh et al. [110] | | | | | | |
| Treatment | 30.3±5.9 | | | | | |
| Placebo | 30±3 | | | | | |
| Saraf-Bank et al. [107] | | | | | | |
| Treatment | 31±2.85 | 97.86±1.13 | 0.86±1.07 | 3.41±1.49 | | |
| Placebo | 30±2.82 | 96.96±1.09 | 0.87±1.06 | 3.17±1.32 | | |
| Shirmohammadi et al. [111] | | | | | | |
| Treatment | 31.03±5.11 | 100.8±11.57 | | | | |
| Placebo | 31.3±4.87 | 99.42±11.86 | | | | |
| Sohaei et al. [112] | | | | | | |
| Treatment | | | | 3.26±2.26 | | 0.33±0.03 |
| Placebo | | | | 3.45±1.66 | | 0.32±0.25 |
| Soltani et al. [113] | | | | | | |

| | | | | | |
|---|---|---|---|---|---|
| Treatment | | | | 3.7±2.5 | |
| Placebo | | | | 3±1.9 | |
| Tamaddoni et al. [115] | | | | | |
| Treatment | 20.6±1.71 | | | 1.28±0.61 | |
| Placebo | 21.37±1.88 | | | 1.35±0.58 | |
| Yaikwawong et al. [118] | | | | | |
| Treatment | 25.94±4.84 | | | 4.86±1.95 | |
| Placebo | 29.34±4.85 | | | 6.04±3.12 | |
| Yaikwawong et al. [119] | | | | | |
| Treatment | | 88.7±9.15 | | 4.86±1.95 | |
| Placebo | | 95.46±15.32 | | 6.04±3.12 | |
| Yang et al. [120] | | | | | |
| Treatment | 33.7±5.15 | | | | |
| Placebo | 28.88±4.88 | | | | |
| Zohrabi et al. [121] | | | | | |
| Curcumin + Std diet | 28.72±2.9 | | 0.795±0.07 | 0.32±0.15 | 0.46±0.32 |
| Placebo | 29.26±3.39 | | 0.8±0.05 | 0.47±0.27 | 0.388±0.299 |

BMI=Body Mass Index, WC=Waist Circumference, W/H Ratio=Waist Hip Ratio, Cu-P=Curcumin-Phospholipid, HOMA-IR=Homeostatic Model Assessment for Insulin Resistance, HOMA-β= Homeostatic Model Assessment for Beta-cell function, QUICKI= Quantitative Insulin Sensitivity Check Index. **=Median Value.

**Supplementary Table S6**

**Evaluating the effects of curcumin consumption on blood glucose (Glu), fasting blood sugar (FBS), 2-Hour post-prandial blood sugar (PPBS), Oral Glucose Tolerance Test (OGTT), Fasting Plasma Insulin (FPI), Insulin, and HbA1c in the clinical studies on metabolic syndrome.**

| Author, Year | Glu (mg/dL) | FBS (mg/dL) | PPBS (mg/dL) | OGTT (mg/dL) | FPI (μIU/mL) | Insulin (μIU/mL) | HbA1c (%) |
|---|---|---|---|---|---|---|---|
| Afshar et al. [20] | | | | | | | |
| Treatment | | 105.41±29.55 | | | | | |
| Placebo | | 99.96±38.79 | | | | | |
| Alvarenga et al. [22] | | | | | | | |
| Treatment | | | | | | | 6.8±1 |
| Placebo | | | | | | | 6.3±0.9 |
| Asadi et al. [24] | | | | | | | |
| Treatment | | 150.90±58.10 | 207.6±74.7 | | | | 8.18±1.96 |
| Placebo | | 189.70±62.50 | 256.2±78.1 | | | | 9.22±1.72 |
| Asan et al. [25] | | | | | | | |
| Treatment | | 87.30±8.20 | | | | 7.90±9.30 | |
| Placebo | | 89.40±6.80 | | | | 8.10±4.80 | |
| Asghari et al. [26] | | | | | | | |
| Curcumin group | | 176.16±41.84 | | | | 13.73±3.80 | 8.89±1.03 |
| Placebo | | 177.43±59.26 | | | | 14.96±3.70 | 9.3±1.36 |
| Askari et al. [27] | | | | | | | |
| Treatment | | 126.78±61.53 | | | | | |
| Placebo | | 109.61±26.21 | | | | | |
| Atakan et al. [28] | | | | | | | |
| Treatment | | 96.17±17.43 | | | | | |
| Placebo | | 101.83±17.58 | | | | | |
| Bateni et al. [30] | | | | | | | |
| Treatment | | 138.70±45.40 | | | | 8.90±3.50 | 7.55±1.51 |
| Placebo | | 134.80±27.40 | | | | 7.20±6.80 | 7.58±1.44 |
| Campbell et al. [32] | | | | | | | |
| Treatment | 95.82±14.64 | | | | | 41.71±64.25 | |
| Placebo | 93.82±10.96 | | | | | 7.64±1.99 | |
| Chashmniam et al. [33] | | | | | | | |
| Treatment | | 93.00±12.05 | | | | | |
| Placebo | | 91.30±9.12 | | | | | |
| Chuengsmarn et al. [34] | | | | | | | |
| Treatment | | 86.47±9.75 | | 123.35±20.5 | | 15.50±1.6 | 5.6±0.38 |

| | | | | | | |
|---|---|---|---|---|---|---|
| Placebo | | 108.21±11.54 | 155.09±39.8 | | 15.79±1.75 | 6.02±0.46 |
| Cicero et al. [36] | | | | | | |
| Treatment | | 101.00±6.00 | | 15.00±3.00 | | |
| Placebo | | 105.00±8.00 | | 18.00±5.00 | | |
| Darmian et al. [37] | | | | | | |
| Tumeric group | | 147.45± 2.06 | | | | |
| Placebo | | 158.60± 1.84 | | | | |
| Dolati et al. [38] | | | | | | |
| Curcumin group | | 88.20±4.68 | | | 7.83±2.99 | |
| Placebo | | 93.40±17.57 | | | 8.38±3.64 | |
| Fergusona et al. [40] | | | | | | |
| Curcumin group | 93.51±9.55 | | | | | |
| Placebo | 92.61±9.93 | | | | | |
| Funamoto et al. [42] | | | | | | |
| Treatment | 99** | | | | | 6.1** |
| Placebo | 134** | | | | | 6.3** |
| Garg et al. [44] | | | | | | |
| Treatment | 126.13** | | | | | |
| Placebo | 126.13** | | | | | |
| Heshmati et al. [51] | | | | | | |
| Treatment | | 100.17±13.91 | | 12.16±7.43 | | |
| Placebo | | 101.11±11.63 | | 13.02±10.05 | | |
| Hondaei et al. [53] | | | | | | |
| Treatment | | 153.00±33.00 | | | 9.40±6.00 | 11±2 |
| Placebo | | 147.00±40.40 | | | 9.70±4.70 | 11.1±1.8 |
| Ismail et al. [55] | | | | | | |
| Treatment (pediatrics) | | 91.33±9.97 | | | 5.52±4.58 | |
| Placebo | | 87.21±12.00 | | | 7.08±7.39 | |
| Treatment (adults) | | 99.07±17.67 | | | 9.27±5.07 | |
| Placebo | | 88±10.74 | | | 10.61±6.56 | |
| Jamilian et al. [56] | | | | | | |
| Treatment | | 91.20±4.60 | | | 10.10±3.20 | |
| Placebo | | 92.50±4.50 | | | 10.60±2.40 | |
| Jarhahzaden et al. [57] | | | | | | |
| Treatment | | 101.08±10.51 | | | | |
| Placebo | | 97.00±10.94 | | | | |
| Javandoosi et al. [58] | | | | | | |

| | | | | | | |
|---|---|---|---|---|---|---|
| Curcumin | | 101.83±15.63 | | | | |
| Curcumin Complex | | 107.59±13.33 | | | | |
| Placebo | | 102.15±16.34 | | | | |
| Jazayeri-Tehrani et al. [59] | | | | | | |
| Treatment | | 86.30±5.20 | | 6.50±0.90 | | 5.1±0.229 |
| Placebo | | 88.20±5.50 | | 7.60±0.70 | | 5.2±0.188 |
| Jimenez-Osorio et al. [60] | | | | | | |
| Treatment | 120.90±46.56 | | | | | |
| Placebo | 122.60±56.12 | | | | | |
| Karandish et al. [61] | | | | | | |
| Curcumin group | | 103.48±8.87 | | | 15.42±2.71 | |
| Placebo | | 110.80±10.46 | | | 19.09±4.43 | |
| Khajehdehi et al. [64] | | | | | | |
| Treatment | | 155.80±90.90 | 241±130.3 | | | |
| Placebo | | 123.60±41.90 | 233.2±88.5 | | | |
| Kocher et al. [67] | | | | | | |
| Treatment | | 91.90±10.00 | | | 8.90±4.50 | |
| Placebo | | 92.50±9.50 | | | 8.50±3.10 | |
| Majeed et al. [69] | | | | | | |
| Treatment | | 103** | | | | 6** |
| Placebo | | 102** | | | | 5.9** |
| Mamsharifi et al. [70] | | | | | | |
| Treatment | | 85.74±9.85 | | | 7.86±2.14 | |
| Placebo | | 86.76±10.75 | | | 7.86±1.69 | |
| Mankowski et al. [71] | | | | | | |
| Treatment | 92.30±6.70 | | | | | |
| Placebo | 97.10±8.80 | | | | | |
| Mirhafez, 2019 | | | | | | |
| Treatment | | 101.00±8.02 | | | | |
| Placebo | | 107.74±22.69 | | | | |
| Mirhafez et al. [73] | | | | | | |
| Treatment | | 95.20±12.70 | | | | |
| Placebo | | 107.10±46.50 | | | | |
| Mirzabeigi et al. [76] | | | | | | |
| Treatment | | 122.50±35.68 | | | | |
| Placebo | | 116.46±24.96 | | | | |
| Mokhtari et al. [79] | | | | | | |

| | | | | | |
|---|---|---|---|---|---|
| Treatment | | 136.10±32.50 | | 10.60±5.50 | 8.3±2.2 |
| Placebo | | 148.00±45.00 | | 12.90±3.70 | 8.1±1.7 |
| Na et al. [80] | | | | | |
| Treatment | 131.17±31.89 | | | | 7.02±2.04 |
| Placebo | 147.21±37.12 | | | | 7.99±2.86 |
| Neta et al. [81] | | | | | |
| Treatment | | 197.30±118.50 | | 12.10±7.90 | 8.5±2.9 |
| Placebo | | 151.20±62.90 | | 10.00±6.90 | 7.2±1.4 |
| Osali, [83] | | | | | |
| Curcumin group | 111.40±9.59 | | | | |
| Placebo | 182.10±51.34 | | | | |
| Panahi et al. [85] | | | | | |
| Treatment | | 129.72±9.81 | | | 6.38±1.23 |
| Placebo | | 142.60±36.66 | | | 5.84±1 |
| Panahi et al. [87] | | | | | |
| Treatment | | 100.27±11.92 | | 10.53±2.64 | 5.95±1.13 |
| Placebo | | 99.33±10.27 | | 11.90±3.37 | 5.77±0.53 |
| Panahi et al. [92] | | | | | |
| Treatment | 154.00±34.00 | | | 19.00±2.00 | 6.5±1 |
| Placebo | 171.00±26.00 | | | 21.00±2.00 | 7.3±0.8 |
| Rahimi, 2016 | | | | | |
| Treatment | | 120.29±38.01 | | | 7.31±1.54 |
| Placebo | | 176.00±61.56 | | | 9±2.33 |
| Rahmani, 2016 | | | | | |
| Treatment | | 107.57±28.34 | | | 5.53±1.27 |
| Placebo | | 118.18±47.35 | | | 7.53±1.43 |
| Reis et al. [96] | | | | | |
| Treatment | | 80.80±11.10 | | | |
| Placebo | | 94** | | | |
| Saadati et al. [100] | | | | | |
| Treatment | 93.96±15.87 | | | 10.26±5.02 | |
| Placebo | 96.24±14.39 | | | 11.17±6.33 | |
| Sadeghzadeh et al. [105] | | | | | |
| Curcumin group | | 106.26±29.50 | 3.30±1.33 | | |
| Placebo | | 106.85±26.07 | 3.40±2.15 | | |
| Sangouni et al. [106] | | | | | |
| Curcumin + Placebo | | 126.20±8.50 | | | |

| | | | |
|---|---|---|---|
| Placebo | 126.30±13.80 | | |
| Saraf-Bank et al. [107] | | | |
| Treatment | 85.97±7.02 | 14.81 ± 1.51 | |
| Placebo | 86.91±4.88 | 13.58 ± 1.52 | |
| Sohaei et al. [112] | | | |
| Treatment | 104.85±7.68 | 12.35±6.79 | |
| Placebo | 104.37±8.94 | 13.29±6.17 | |
| Soltani et al. [113] | | | |
| Treatment | 116.30±24.40 | 12.60±7.20 | 6.3±0.8 |
| Placebo | 114.90±24.30 | 10.60±5.90 | 6.3±0.8 |
| Tamaddoni et al. [115] | | | |
| Treatment | | 5.82±2.82 | |
| Placebo | | 6.24±2.59 | |
| Yaikwawong et al. [119] | | | |
| Treatment | 115.49±17.91 | | 6.12±0.84 |
| Placebo | 130.7±19.1 | | 6.47±1.1 |
| Yang et al. [120] | | | |
| Treatment | 114.82±16.15 | | 6.2±0.73 |
| Placebo | 124.32±11.91 | | 6.56±1.06 |
| Zohrabi et al. [121] | | | |
| Curcumin + Standard diet | 85.24±9.85 | 45.61±60.86 | |
| Placebo | 88.45±10.91 | 104.19±110.19 | |

Glu=Blood Glucose, FBS=Fasting blood sugar, PPBS=2-Hour post-prandial blood sugar, OGTT=Oral Glucose Tolerance Test, FPI=Fasting Plasma Insulin **=Median Value.

**Supplementary Table S7**

**Evaluating the effects of Curcumin consumption on Triglyceride (TG), Total Cholesterol (TC), LDL Cholesterol (LDL), VLDL Cholesterol (VLDL), HDL Cholesterol (HDL), Systolic Blood Pressure (SBP), and Diastolic Blood Pressure (DBP) in the clinical studies on metabolic syndrome.**

| Author, Year | TG (mg/dL) | TC (mg/dL) | LDL (mg/dL) | VLDL (mg/dL) | HDL (mg/dL) | SBP (mmHg) | DBP (mmHg) |
|---|---|---|---|---|---|---|---|
| Abed & Abdulridha [18] | | | | | | | |
| Treatment | | | | | | 142.07±20.73 | 92.27±20.54 |
| Placebo | | | | | | 155.67±21.18 | 96.39±22.24 |
| Adibian et al. [19] | | | | | | | |
| Treatment | 109.00±36.00 | 163±39 | 108±36 | | 30±2 | | |
| Placebo | 121.00±44.00 | 175±47 | 118±47 | | 32±5 | | |
| Afshar et al. [20] | | | | | | | |
| Treatment | 132.00±43.37 | 141.22±32.98 | 82.15±21.52 | | 33.81±8.14 | | |
| Placebo | 161.26±119.30 | 152.56±42.69 | 86.26±29.15 | | 34.63±9.73 | | |
| Alvarenga et al. [22] | | | | | | | |
| Treatment | 108.30±66.00 | 137.1±35 | 74.6±28 | | 40.7±12.5 | | |
| Placebo | 139.40±86.90 | 148.9±41.5 | 81.9±39.4 | | 39.1±9.7 | | |
| Asan et al. [25] | | | | | | | |
| Treatment | 99.80±44.50 | 187.5±26.9 | 116.1±25.8 | | 51.4±15.4 | | |
| Placebo | 135.80±63.50 | 202±20.9 | 122.7±26 | | 52.1±18.5 | | |
| Asghari et al. [26] | | | | | | | |
| Curcumin group | 172.73±73.67 | 205.61±73.3 | 116.73±78.94 | | 54.33±16.71 | | |
| Placebo | 180.11±76.06 | 245.12±48.05 | 154.54±54.21 | | 54.56±13.24 | | |
| Askari et al. [27] | | | | | | | |
| Treatment | 144.00±103.00 | 181.34±43.82 | 100.39±33.21 | 26.61±15.74 | 41.52±5.18 | | |
| Placebo | 144.57±58.70 | 188.08±48.81 | 108.22±29.57 | 28.82±11.66 | 40.3±5.47 | | |
| Atakan et al. [28] | | | | | | | |
| Treatment | 134.57±25.32 | 176.09±24.91 | 109.44±14.47 | | 64.83±23.13 | | |
| Placebo | 136.74±19.70 | 178.89±35.54 | 111.34±17.44 | | 57.06±23.75 | | |
| Barber-Chamoux et al. [29] | | | | | | | |
| Treatment | | | | | | 123.33±16.55 | 75.14±10.24 |
| Placebo | | | | | | 125.64±20.02 | 74.56±11.19 |
| Bateni et al. [30] | | | | | | | |
| Treatment | 201.70±89.06 | 174.5±34.9 | 86.4±39.8 | | 45.6±13.04 | 114.9±27.5 | 76.2±17.4 |
| Placebo | 224.09±94.05 | 175.6±32.5 | 93.9±40.9 | | 38.6±10.5 | 132.1±20.1 | 81.8±14.3 |
| Boshagh et al. [31] | | | | | | | |

| | | | | | | | |
|---|---|---|---|---|---|---|---|
| Treatment | 134.18±62.42 | 162.07±36.48 | 108.4±40.45 | | 73.07±22.61 | 121.1±8 | 83.3±6.7 |
| Placebo | 179.24±72.94 | 178.58±44.87 | 113.27±40.45 | | 68.79±18.46 | 127.2±6.4 | 88.9±7.7 |
| Campbell et al. [32] | | | | | | | |
| Treatment | | | 103.08±49.53 | | 54.56±38.92 | | |
| Placebo | | | 86.38±47.37 | | 48.82±18.22 | | |
| Chashmniam et al. [33] | | | | | | | |
| Treatment | 176.52±72.5 | 208.12±40.55 | 123.43±35.45 | | 43.03±8.1 | | |
| Placebo | 147.15±73.1 | 193.95±28.26 | 110.02±33.81 | | 45.11±10.24 | | |
| Chuengsmarn et al. [35] | | | | | | | |
| Treatment | 82.98±68.06 | | | | | | |
| Placebo | 166.87±114.43 | | | | | | |
| Cicero et al. [36] | | | | | | | |
| Treatment | 151.00±16.00 | 185±13 | 111±8 | | 44±4 | 126±6 | 84±7 |
| Placebo | 157.00±19.00 | 189±19 | 116±14 | | 42±3 | 127±9 | 81±6 |
| Darmian et al. [37] | | | | | | | |
| Turmeric group | 177.86±4.13 | | | | 36.55±1.82 | 128.04±2.32 | 84.03±1.39 |
| Placebo | 186.22±2.88 | | | | 30.19±1.44 | 133.75±3.16 | 87.79±1.14 |
| Dolati et al. [38] | | | | | | | |
| Curcumin group | 108.30±63.27 | 174.7±26.91 | 96.7±27.64 | | 58.4±18.22 | | |
| Placebo | 83.70±22.98 | 164.3±25.25 | 91.3±23.79 | | 54.1±16.6 | | |
| Fergusona et al. [40] | | | | | | | |
| Curcumin group | 129.3** | 255.2±68.91 | 168.19±59.03 | | 59.53±19.68 | | |
| Placebo | 95.65** | 253.4±34.45 | 173.2±31.17 | | 58.76±18.02 | | |
| Funamoto et al. [42] | | | | | | | |
| Treatment | 120** | | 100.9±19.2 | | 60.2±13.1 | 129.3±17.8 | 69.3±11.1 |
| Placebo | 118** | | 101.9±26.8 | | 52±12.9 | 125.1±18.1 | 70.5±13.8 |
| Ghaffari et al. [45] | | | | | | | |
| Turmeric only | 202.00±80.20 | 204±39.1 | 119±31.8 | | 44.7±7.19 | | |
| Placebo | 248.00±106.00 | 217±32.1 | 128±28.3 | | 40.8±7.67 | | |
| Hondaei et al. [53] | | | | | | | |
| Treatment | | | | | | 120±16.5 | 80±12.5 |
| Placebo | | | | | | 120±16.6 | 70±7.6 |
| Jamilian et al. [56] | | | | | | | |
| Treatment | 154.00±29.40 | 175.5±39.7 | 103.6±38.5 | 30.8±5.8 | 41±9.6 | | |
| Placebo | 153.80±33.30 | 179.7±28.1 | 106.4±29.4 | 30.8±6.6 | 42.5±8 | | |
| Jarhahzaden et al. [57] | | | | | | | |
| Treatment | 141.78±65.57 | 186.5±36.49 | 108.4±26.83 | 32.53±16.52 | 42.34±4.13 | | |

| | | | | | | | |
|---|---|---|---|---|---|---|---|
| Placebo | 155.62±85.35 | 182.62±29.36 | 104.6±22.99 | 33.63±16.55 | 44.59±7.17 | | |
| Javandoosi et al. [58] | | | | | | | |
| Curcumin | 153.53±60.90 | 246.67±48.9 | 159.48±40.55 | | 51.97±7.39 | | |
| Curcumin Complex | 189.86±75.99 | 224.68±47.6 | 137.3±40.23 | | 48.26±10.17 | | |
| Placebo | 154.28±54.36 | 224.9±40.59 | 146.7±27.27 | | 48.74±7.89 | | |
| Jazayeri-Tehrani et al. [59] | | | | | | | |
| Treatment | 142.50±49.90 | 195.2±19.8 | 114.6±20.5 | | 51.4±6.6 | 118.2±4.4 | 77.9±2.9 |
| Placebo | 175.30±62.50 | 205±20.5 | 125.7±22.2 | | 43.8±5.4 | 119.6±4.9 | 78.7±3.4 |
| Jimenez-Osorio et al. [60] | | | | | | | |
| Treatment | 162.30±70.9 | 218.4±60.85 | | | | 128.7±23.28 | 77.9±10.05 |
| Placebo | 189.00±108.85 | 251.8±108.42 | | | | 123.1±17.27 | 73.8±11.03 |
| Karandish et al. [62] | | | | | | | |
| Curcumin group | 107.48±11.90 | 175.71±17.01 | 97.74±18.9 | | 56.47±6.14 | | |
| Placebo | 123.10±18.60 | 184.25±24.4 | 109.48±22.77 | | 50.15±5.34 | | |
| Khajehdehi et al. [64] | | | | | | | |
| Treatment | 196.70± 118.10 | 187.5± 59.2 | 110.3± 41.9 | | 40.4±9 | 125.8±10.9 | 75.8±5.9 |
| Placebo | 190.50± 129.90 | 163.7± 46.5 | 84.4± 12.2 | | 36.2±9.6 | 123.6±11.9 | 83.6±5.9 |
| Khajehdehi et al. [65] | | | | | | | |
| Treatment | | | | | | 124±18 | 77±8 |
| Placebo | | | | | | 123±15 | 76±7 |
| Kocher et al. [67] | | | | | | | |
| Treatment | 111.10±47.20 | 237.2±45.9 | 160.8±36.4 | | 55.6±14.9 | | |
| Placebo | 121.40±53.20 | 239.3±50.1 | 162.5±38 | | 55.3±15.1 | | |
| Krishnareddy et al. [68] | | | | | | | |
| Treatment | 176.20±42.70 | 234.3±20.1 | 100.4±15.1 | | 53.21±4.3 | 121.3±3.4 | 124.7±3.3 |
| Placebo | 191.30±34.20 | 258.2±32.7 | 120.7±18.1 | | 53.21±4.3 | 124.7±3.3 | 84.54±2.7 |
| Majeed et al. [69] | | | | | | | |
| Treatment | 153** | 153** | 79** | 31** | 44.5** | 131.38±7.69 | 82.74±6.64 |
| Placebo | 153** | 167** | 99** | 30** | 42.0** | 130.12±8.76 | 83.48±7.04 |
| Mamsharifi et al. [70] | | | | | | | |
| Treatment | 168.52±34.69 | 186.93±31.69 | 112.78±31.15 | 35.16±5.54 | 38.71±5.48 | | |
| Placebo | 161.15±35.02 | 186.16±24.53 | 112.53±25.99 | 33±6.29 | 40.5±5.07 | | |
| Mirhafez et al. [73] | | | | | | | |
| Treatment | 130.37±83.68 | 195.12±40.61 | 130.98±42.75 | | 41.93±13.93 | | |
| Placebo | 140.53±66.39 | 187.76±31.34 | 119.73±24.6 | | 42.41±10.59 | | |
| Mirhafez et al. [74] | | | | | | | |
| Treatment | 161** | 199.4±44.5 | 118±34.4 | | 42.3±7.8 | 115.7±13.4 | 81.1±8.6 |

| | | | | | | | |
|---|---|---|---|---|---|---|---|
| Placebo | 130.5** | 188.7±36 | 107.5±32.1 | | 43.5±8.9 | 116.6±15.3 | 83±9.7 |
| Mirzabeigi et al. [76] | | | | | | | |
| Treatment | 120.15±10.47 | 154.13±40.1 | 84.15±28.15 | 22.68±8.1 | 41.13±9.2 | | |
| Placebo | 108.75±53.32 | 151.07±50.31 | 81.38±43.41 | 17.25±7.31 | 42.28±6.54 | | |
| Mohammadi et al. [77] | | | | | | | |
| Treatment | 193.71±136.86 | 173.26±40.26 | 145.25±53.26 | | | | |
| Placebo | 150.97±135.6 | 171.36±39.92 | 145.72±52.75 | | | | |
| Mohammadi et al. [78] | | | | | | | |
| Curcumin group | | | | | 42.53±9.69 | | |
| Placebo | | | | | 41.64±8.68 | | |
| Mokhtari et al. [79] | | | | | | | |
| Treatment | 138.90±72.60 | 148.8±33.4 | 79.9±22 | 27.8±14.5 | 41.8±9.1 | | |
| Placebo | 147.70±47.00 | 165±44.2 | 96.2±41.2 | 29.5±9.4 | 39.3±5.1 | | |
| Na et al. [80] | | | | | | | |
| Treatment | 157.63± 49.60 | 215.71± 41.76 | 146.85± 39.83 | | 54.91±11.21 | | |
| Placebo | 186.89± 66.43 | 228.15± 45.99 | 160.46± 45.22 | | 51.82±8.89 | | |
| DeFigueiredo Neta et al. [81 | | | | | | | |
| Treatment | 186.20±82.80 | | 95.9±37.7 | | 62.3±11.2 | 131.6±17.4 | 72.5±9.4 |
| Placebo | 154.30±78.20 | | 98.2±40.9 | | 61.2±12.7 | 135.3±16.5 | 74.2±10.5 |
| Osali [83] | | | | | | | |
| Curcumin group | 164.60±24.13 | | | | 52.6±3.2 | 132.1±10.26 | |
| Placebo | 180.30±55.20 | | | | 44.7±2.79 | 152.7±11.47 | |
| Panahi et al. [84] | | | | | | | |
| Treatment | 183.62±22.34 | 195.88** | 165.58** | | 36.96±6.93 | | |
| Placebo | 187.80±39.83 | 186** | 161.8** | | 36.68±6.93 | | |
| Panahi et al. [85] | | | | | | | |
| Treatment | | | | | | 126.8±9.68 | 80.78±5.02 |
| Placebo | | | | | | 129±7.95 | 85.1±7.52 |
| Panahi et al. [89] | | | | | | | |
| Treatment | 125.32±62.70 | 158.29±40.75 | 100.35±25.26 | | 45.09±8.33 | | |
| Placebo | 155.68±68.68 | 199.37±59.65 | 129.95±44.23 | | 46.56±10.06 | | |
| Panahi et al. [91] | | | | | | | |
| Treatment | 205.48±64.52 | 195.48±33.39 | 160.94±28.32 | | | 120.7±6.9 | 78.9±6.2 |
| Placebo | 187.06±44.34 | 213.98±55.12 | 168.9±29.91 | | | 120.7±6.9 | 79.1±6.5 |
| Porasgari et al. [95] | | | | | | | |
| Curcumin group | 135.75±51.8 | 179.25±51.8 | 96.25±9.43 | | 41.62±12.13 | | |
| Placebo | 90.87±46.31 | 179.5±45.26 | 115.12±39.09 | | 42.12±21.39 | | |

| | | | | | | | |
|---|---|---|---|---|---|---|---|
| Rahimi et al. [96] | | | | | | | |
| Treatment | 131** | 158.62±44.06 | 91.04±28.72 | | 60.95±15.68 | | |
| Placebo | 113** | 149±24.62 | 84±12.59 | | 55±11.09 | | |
| Rahmani et al. [97] | | | | | | | |
| Treatment | 173.43±95.44 | 174.38±39.56 | 95.59±28.22 | | 46.68±10.98 | | |
| Placebo | 153.58±50.12 | 196.82±37.04 | 125±24.23 | | 46.72±15.38 | | |
| Reis et al. [98] | | | | | | | |
| Treatment | 144** | 170.55** | 83.43±22.02 | | 59.55±7.8 | | |
| Placebo | 140.35±15.51 | 191** | 107.23±37.53 | | 49** | | |
| Rezaei et al. [99] | | | | | | | |
| Treatment | 163.60±94.90 | 179.8±44.3 | 89.9±26.9 | | 50.6±15.4 | | |
| Placebo | 151.20±69.90 | 183.8±40.1 | 94.2±22.4 | | 50.2±11.4 | | |
| Saadati et al. [100] | | | | | | | |
| Treatment | 145.96±64.70 | 170.19±31.99 | 103.66±29.28 | | 37.33±9.47 | | |
| Placebo | 166.00±81.36 | 187.65±37.7 | 114.46±36.34 | | 39.98±0.34 | | |
| Sadeghzadeh et al. [105] | | | | | | | |
| Curcumin group | 108.66±18.81 | 166.44±30.41 | 137.33±43.88 | | | | |
| Placebo | 102.50±19.00 | 171.5±32.32 | 112.77±55.03 | | | | |
| Sangouni et al. [106] | | | | | | | |
| Curcumin + Placebo | 166.70±72.80 | 186.5±30.4 | 75.3±5.4 | | 42±5.9 | 130.8±8.4 | 91.5±6.6 |
| Placebo | 237.80±88.60 | 237.7±53.9 | 87.3±7.4 | | 34.2±8.6 | 134.9±15.2 | 91.8±9.9 |
| Saraf-Bank et al. [107] | | | | | | | |
| Treatment | 102.16±1.46 | 161.41±30.99 | 85.12±1.32 | | 50.77±1.18 | 111.36±12.01 | 75.14±9.09 |
| Placebo | 106.30±1.42 | 162.32±28.02 | 85.41±1.28 | | 50.73±1.2 | 109.56±9.79 | 73.72±7.27 |
| Sohaei et al. [112] | | | | | | | |
| Treatment | 166.07±110.53 | 182.5±38.35 | 96.16±29.31 | | 50.83±8.14 | | |
| Placebo | 148.45±95.48 | 177.87±32.68 | 90.32±22.58 | | 52.67±11.25 | | |
| Soltani et al. [113] | | | | | | | |
| Treatment | | | | | | 124.8±12.2 | 84.6±7.6 |
| Placebo | | | | | | 125.3±11.8 | 85±12.1 |
| Srinivasan et al. [114] | | | | | | | |
| Treatment | | | | | | | 77.8±7.97 |
| Placebo | | | | | | 129.6±13.74 | 77.17±8.13 |
| Tamaddoni et al. [115] | | | | | | | |
| Treatment | 161.90±30.90 | 162.19±19.45 | 89.52±19.96 | | 40.29±5.93 | | |
| Placebo | 172.10±38.70 | 155.73±24.29 | 84.28±29.41 | | 37.03±8.86 | | |
| Yaikwawong et al. [119] | | | | | | | |

| | | | | | |
|---|---|---|---|---|---|
| Treatment | | | 86.77±25.47 | | |
| Placebo | | | 105.98±29.45 | | |
| Yang et al. [120] | | | | | |
| Treatment | 160.79±75.46 | 175.86±30.63 | 106.51±25.02 | 25.55±22.37 | 43.76±9.54 |
| Placebo | 144.65±56.06 | 167.53±37.6 | 102.75±26.76 | 28.32±14.59 | 40.92±9.47 |

TG=Triglyceride, TC=Total Cholesterol, LDL=LDL Cholesterol, VLDL=VLDL Cholesterol, HDL=HDL Cholesterol, SBP=Systolic Blood Pressure, DBP=Diastolic Blood Pressure, **=Median Value.

**Supplementary Table S8**

**Summary of outcomes related to parameters associated with inflammation in the treatment group compared to control group that highlights the effects of curcumin supplementation in the controlled clinical studies**

| First Author, Year | CRP | IL6 | IL8 | IL10 | IL-1β | IL-1ra | TNF-α | NF-κB | IFN-γ | TGFβ | MCP-1 | Chemerin | VEGF | Resistin | Visfatin | Fe-A | NLRP3 | MPO |
|---|---|---|---|---|---|---|---|---|---|---|---|---|---|---|---|---|---|---|
| Adibian et al. [19] | ↓ | - | - | - | - | - | - | - | - | - | - | - | - | - | - | - | - | - |
| Afshar et al. [20] | ↓ | - | - | - | - | - | - | - | - | - | - | - | - | - | - | - | - | - |
| Alizadeh et al. [21] | ↓ | - | - | - | - | - | ↓ | - | - | - | - | - | - | - | - | - | - | - |
| Alvarenga et al. [22] | ↓ | - | - | - | ↓ | - | - | ↓ | - | - | - | - | - | - | - | - | ↓ | - |
| Alvarenga et al. [23] | - | ↓ | - | - | - | - | ↓ | - | - | - | - | - | - | - | - | - | - | - |
| Asan et al. [25] | ↓ | - | - | - | - | - | - | - | - | - | - | - | - | - | - | - | - | - |
| Asghari et al. [26] | ↓ | - | - | - | - | - | - | - | - | - | - | - | ↑ | - | - | - | - | - |
| Askari et al. [27] | ↑ | - | - | - | - | - | - | - | - | - | - | - | - | - | - | - | - | - |
| Boshagh et al. [31] | ↓ | - | - | - | - | - | - | - | - | - | - | - | - | - | - | - | - | - |
| Darmian et al. [37] | ↓ | - | - | - | - | - | - | - | - | - | - | - | - | - | - | - | - | - |
| Dolati et al. [39] | - | - | - | - | - | - | - | - | - | - | - | - | - | - | - | - | - | ↑ |
| Funamoto et al. [42] | ↓ | - | - | - | - | - | - | - | - | - | - | - | - | - | - | - | - | - |
| Garg et al. [44] | ↔ | - | - | - | - | - | - | - | - | - | - | - | - | - | - | - | - | - |
| Haroyan et al. [48] | ↓ | - | - | - | - | - | - | - | - | - | - | - | - | - | - | - | - | - |
| Ismail et al. [54] | - | - | - | - | - | - | - | - | - | - | - | - | - | ↓ | - | ↓ | - | - |
| Jazayeri-Tehrani et al. [59] | ↓ | ↓ | - | - | - | - | ↓ | - | - | - | - | - | - | - | - | - | - | - |
| Khajehdehi et al. [64] | - | - | ↓ | - | - | - | ↔ | - | - | ↓ | - | - | - | - | - | - | - | - |
| Kisiolek et al. [66] | ↓ | ↓ | - | - | - | ↓ | - | - | - | - | - | - | - | - | - | - | - | - |
| Kocher et al. [67] | ↔ | ↓ | - | - | - | - | - | - | - | - | - | - | - | - | - | - | - | - |
| Krishnareddy et al. [68] | ↑ | ↓ | - | - | - | - | - | - | - | - | - | - | - | - | - | - | - | - |
| Majeed et al. [69] | ↓ | - | - | - | - | - | - | - | - | - | - | - | - | - | - | - | - | - |
| Mamsharifi et al. [70] | ↓ | - | - | - | - | - | - | - | - | - | - | - | - | - | - | - | - | - |
| Mirzabeigi et al. [76] | ↑ | - | - | - | - | - | - | - | - | - | - | - | - | - | - | - | - | - |
| Mokhtari et al. [79] | ↓ | - | - | - | - | - | - | - | - | - | - | - | - | - | - | - | - | - |
| Nowak et al. [82] | ↑ | ↓ | - | - | - | - | - | - | ↔ | - | - | - | - | - | - | - | - | - |
| Osali [83] | ↓ | ↓ | - | ↑ | - | - | - | - | - | - | - | - | - | - | - | - | - | - |
| Panahi et al. [85] | ↓ | - | - | - | - | - | - | - | - | - | - | - | - | - | - | - | - | - |
| Panahi et al. [87] | - | ↓ | - | - | - | - | ↓ | - | - | ↓ | ↓ | - | - | - | - | - | - | - |
| Panahi et al. [92] | ↑ | - | - | - | - | - | - | - | - | - | - | - | - | - | - | - | - | - |
| Saadati et al. [100] | ↓ | - | - | - | - | - | ↔ | ↓ | - | - | - | - | - | - | - | - | - | - |
| Saraf-Bank et al. [108] | ↓ | ↑ | - | - | - | - | - | - | - | - | - | ↓ | - | - | - | - | - | - |
| Sedighiyan et al. [109] | - | - | - | - | - | - | - | - | - | - | ↓ | - | - | ↓ | ↓ | - | - | - |

| | | | | | | | | | | | | | | | | | | |
|---|---|---|---|---|---|---|---|---|---|---|---|---|---|---|---|---|---|---|
| Sohaei et al. [112] | ↑ | - | - | - | - | - | - | - | - | - | - | - | - | - | - | - | - | - |
| Soltani et al. [113] | ↔ | - | - | - | - | - | - | - | - | - | - | - | - | - | ↓ | - | - | - |
| Tamaddoni et al. [115] | ↓ | - | - | - | - | - | - | - | - | - | - | - | - | - | - | - | - | - |
| Yaikwawong et al. [118] | - | ↓ | - | - | ↓ | - | ↓ | - | - | - | - | - | - | - | - | - | - | - |
| Yaikwawong et al. [119] | ↓ | ↓ | - | - | ↓ | - | ↓ | - | - | - | - | - | - | - | - | - | - | - |

↑=Value increase in the treatment group compared to control group, ↓=Value decrease in the treatment group compared to control group, ↔ = Value approximately or exactly equal between treatment group and control group, CRP=C reactive protein, IL6=Interleukin-6, IL8=Interleukin-8, IL10=Interleukin-10, IL-1β= Interleukin-1β, IL-1ra= Interleukin-1 receptor antagonist, TNF-α= Tumor necrosis factor, NF-κB= Nuclear factor kappa B cell, IFN-γ= Interferon, TGFβ= Transforming growth factor beta, MCP-1= Monocyte chemoattractant protein-1, VEGF=Vascular Endothelial Growth Factors, Fe-A=Fetuin-A, NLRP3= NOD-, LRR-, and Pyrin Domain-Containing Protein 3, MPO= Myeloperoxidase.

**Supplementary Table S9**

**Evaluating the effects of curcumin consumption on inflammation: Interleukin-6 (IL-6), Interleukin-8 (IL-8), Interleukin-10 (IL-10), Interleukin-1β (IL-1β), Interleukin-1ra (IL-1ra), and C-reactive Protein (CRP) in the clinical studies on metabolic syndrome.**

| Author, Year | IL-6 (pg/mL) | IL-8 (pg/mL) | IL-10 (pg/mL) | IL-1β (pg/mL) | IL-1ra (pg/mL) | CRP (mg/L) |
|---|---|---|---|---|---|---|
| Adibian et al. [19] | | | | | | |
| Treatment | | | | | | 2.9±2.9 |
| Placebo | | | | | | 3.4±4.2 |
| Afshar et al. [20] | | | | | | |
| Treatment | | | | | | 6.9±3.58 |
| Placebo | | | | | | 11.1±7.17 |
| Alizadeh et al. [21] | | | | | | |
| Treatment | | | | | | 4.76±1.4 μM |
| Placebo | | | | | | 6.8±2 μM |
| Alvarenga et al. [22] | | | | | | |
| Treatment | | | | 0.96** | | 2** |
| Placebo | | | | 1.05** | | 3.3** |
| Alvarenga et al. [23] | | | | | | |
| Treatment | 11.8±9.94 | | | | | |
| Placebo | 14.3±14 | | | | | |
| Asan et al. [25] | | | | | | |
| Treatment | | | | | | 4±5 |
| Placebo | | | | | | 6±6 |
| Asghari et al. [26] | | | | | | |
| Curcumin group | | | | | | 7.31±2.21 |
| Placebo | | | | | | 9.11±2.55 |
| Askari et al. [27] | | | | | | |
| Treatment | | | | | | 3.26±11.47 |
| Placebo | | | | | | 2.63±8.3 |
| Boshagh et al. [31] | | | | | | |
| Treatment | | | | | | 0.48±0.74 |
| Placebo | | | | | | 2.39±5.11 |
| Darmian et al. [37] | | | | | | |
| Tumeric group | | | | | | 2.7±0.25 |
| Placebo | | | | | | 2.94±0.12 |
| Funamoto et al. [42] | | | | | | |
| Treatment | | | | | | 5** |
| Placebo | | | | | | 9** |
| Garg et al. [44] | | | | | | |
| Treatment | | | | | | 58 μg/mL** |
| Placebo | | | | | | 58 μg/mL** |
| Haroyan et al. [48] | | | | | | |
| Curamed | | | | | | 3.88±0.33 |
| Curamin | | | | | | 3.7±0.3 |
| Placebo | | | | | | 3.9±0.29 |
| Jazayeri-Tehrani et al. [59] | | | | | | |
| Treatment | 3.81±1.63 | | | | | 3.6±1.58 |
| Placebo | 7.02±7.6 | | | | | 5.2±2.47 |
| Kisiolek et al. [66] | | | | | | |
| CURF group | 1.01±0.99 | | | | 107.32±35.4 | 0.75±0.77 |
| CURS group | 2.16±2.22 | | | | 130.21±22.1 | 1.68±1.93 |
| Placebo | 1.94±1.87 | | | | 138.93±89.2 | 2.2±2.33 |
| Khajehdehi et al. [64] | | | | | | |
| Treatment | | 30.6±75.2 | | | | |
| Placebo | | 88.4±116.3 | | | | |

| | | | | |
|---|---|---|---|---|
| Kocher et al. [67] | | | | |
| Treatment | 7.2±7.9 | | | 3.4±1.8 |
| Placebo | 9.6±15.7 | | | 3.4±2.0 |
| Krishnareddy et al. [68] | | | | |
| Treatment | 17.69±7.31 | | | 1±0.43mg/mL |
| Placebo | 23.08±6.92 | | | 0.96±0.29mg/ml |
| Majeed et al. [69] | | | | |
| Treatment | | | | 4.5±0.6 |
| Placebo | | | | 4.9±1 |
| Mamsharifi et al. [70] | | | | |
| Treatment | | | | 4.93±1.21 |
| Placebo | | | | 5.44±1.4 |
| Mirzabeigi et al. [76] | | | | |
| Treatment | | | | 0.59±0.76 |
| Placebo | | | | 0.23±0.18 |
| Mokhtari et al. [79] | | | | |
| Treatment | | | | 15.4±16.3 |
| Placebo | | | | 22.1±17.5 |
| Nowak et al. [82] | | | | |
| Treatment | 3.6** | | | 0.63** |
| Placebo | 4.3** | | | 0.44** |
| Osali [83] | | | | |
| Curcumin group | 15.68±2.38 | 10.72±1.41 | | 1.18±0.67 pg/mL |
| Placebo | 18.68±2.71 | 7.85±1.15 | | 2.31±0.79 pg/mL |
| Panahi et al. [85] | | | | |
| Treatment | | | | 4.4±1.74 |
| Placebo | | | | 7.16±1.49 |
| Panahi et al. [88] | | | | |
| Treatment | 1.37±1.52 | | | |
| Placebo | 1.81±0.55 | | | |
| Panahi et al. [92] | | | | |
| Treatment | 10.6±3.3 g/L | | | |
| Placebo | 9.9±2.4 g/L | | | |
| Saadati et al. [101] | | | | |
| Treatment | | | | 0.47 ± 0.42 |
| Placebo | | | | 0.53 ± 0.51 |
| Saraf-Bank et al. [108] | | | | |
| Treatment | 60.74±34.8 | | | 0.14±0.03 |
| Placebo | 52.51±21.71 | | | 0.16±0.04 |
| Sohaei et al. [112] | | | | |
| Treatment | | | | 46.2±40 |
| Placebo | | | | 38.3±33.8 |
| Soltani et al. [113] | | | | |
| Treatment | | | | 1.1±0.5 |
| Placebo | | | | 1.1±0.6 |
| Tamaddoni et al. [115] | | | | |
| Treatment | | | | 2.57±0.83 |
| Placebo | | | | 3.01±1.01 |
| Yaikwawong et al. [119] | | | | |
| Treatment | 6.12±1.85 | | 0.31±0.26 | 2.6±2.88 |
| Placebo | 15.84±3.05 | | 0.74±0.31 | 3.75±5.97 |

CRP=C reactive protein, IL-6=Interleukin-6, IL-8=Interleukin-8, IL-10=Interleukin-10, IL-1β=Interleukin-1β, IL-1ra=Interleukin-1ra, **=Median Value.

**Supplementary Table S10**

**Evaluating the effects of curcumin consumption on inflammation; Tumor necrosis factor (TNF-α), Nuclear factor kappa B (NF-kB), Transforming growth factor beta (TGF-β), Vascular Endothelial Growth Factors (VEGF), Resistin, Visfatin in the clinical studies on metabolic syndrome.**

| Author, Year | TNF-α (pg/mL) | NF-kB | TGF-β (pg/mL) | VEGF | Resistin (ng/mL) | Visfatin (ng/mL) |
|---|---|---|---|---|---|---|
| Alizadeh et al. [21] | | | | | | |
| Treatment | 10.57±1.4 µM | | | | | |
| Placebo | 12.57±1.86 µM | | | | | |
| Alvarenga et al. [22] | | | | | | |
| Treatment | | 0.52** | | | | |
| Placebo | | 1.07** | | | | |
| Alvarenga et al. [23] | | | | | | |
| Treatment | 6.17** | | | | | |
| Placebo | 16.4** | | | | | |
| Asghari et al. [26] | | | | | | |
| Curcumin group | | | | 1532.24±271.88 | | |
| Placebo | | | | 1414.33±273.32 | | |
| Ismail et al. [54] | | | | | | |
| Treatment | | | | 3.85±1.6 | | |
| Placebo | | | | 7.42±5.38 | | |
| Jazayeri-Tehrani et al. [59] | | | | | | |
| Treatment | 7.3±2.9 | | | | | |
| Placebo | 13.7±4 | | | | | |
| Khajehdehi et al. [64] | | | | | | |
| Treatment | 18.4±24.1 | | 397.3±55.2 | | | |
| Placebo | 18.4±22.3 | | 412.5±129.8 | | | |
| Panahi et al. [87] | | | | | | |
| Treatment | 63.02±8.18 | | 2490±990 | | | |
| Placebo | 78.9±8.17 | | 5490±1050 | | | |
| Saadati et al. [100] | | | | | | |
| Treatment | 16.51±4.5 | 1.65±0.63 | | | | |
| Placebo | 16.54±2.1 | 2.03±0.51 | | | | |
| Sedighiyan et al. [109] | | | | | | |
| Treatment | | | | | 119.46±30.41 | 19.09±4.69 |
| Placebo | | | | | 126.24±36.58 | 19.57±4.08 |
| Soltani et al. [113] | | | | | | |
| Treatment | | | | | | 15.6±25.8 |
| Placebo | | | | | | 22.3±40.8 |

TNF-α=Tumor necrosis factor, NF-kB=Nuclear factor kappa B, TGF-β = Transforming growth factor beta, VEGF= Vascular Endothelial Growth Factors, **=Median Value.

**Supplementary Table S11**

**Evaluating the effects of curcumin consumption on inflammation; Interferon (IFN-γ), Monocyte chemoattractant protein-1 (MCP-1), Chemerin, Fetuin-A, NLRP3 and Myeloperoxidase (MPO in the clinical studies on metabolic syndrome.**

| Author, Year | IFN-γ (pg/mL) | MCP-1 (ng/mL) | Chemerin (pg/mL) | Fetuin-A | NLRP3 | MPO (ng/mL) |
|---|---|---|---|---|---|---|
| Alvarenga et al. [22] | | | | | | |
| Treatment | | | | | 0.80** | |
| Placebo | | | | | 0.94** | |
| Dolati et al. [39] | | | | | | |
| Curcumin group | | | | | | 10.16±2.7 |
| Placebo | | | | | | 8.75±3.2 |
| Ismail et al. [54] | | | | | | |
| Treatment | | | | 236.67±66.85 | | |
| Placebo | | | | 281.6±102.5 | | |
| Nowak et al. [82] | | | | | | |
| Treatment | 1.2** | | | | | |
| Placebo | 1.2** | | | | | |
| Panahi et al. [87] | | | | | | |
| Treatment | | 97.96±19.99 | | | | |
| Placebo | | 132.86±11.27 | | | | |
| Saraf-Bank et al. [107] | | | | | | |
| Treatment | | | 801.4±403.74 | | | |
| Placebo | | | 850.1±349.31 | | | |
| Sedighiyan et al. [109] | | | | | | |
| Treatment | | 153.97±44.89 | | | | |
| Placebo | | 172.87±52.14 | | | | |

IFN-γ= Interferon, MCP-1= Monocyte chemoattractant protein-1, NLRP3=Nucleotide-binding domain, leucine-rich-containing family, pyrin domain-containing-3, MPO=Myeloperoxidase, **=Median Value.

**Supplementary Table S12**

**Summary of outcomes related to parameters associated with oxidative stress in the treatment group compared to the control group that highlights the effects of curcumin supplementation in the controlled clinical studies**

| First Author, Year | MDA | SOD | GPx | GSH | GSSG | GR | TBARS | TAC | TAS | LDL-ox | PAB | CAT | FRSA | NO |
|---|---|---|---|---|---|---|---|---|---|---|---|---|---|---|
| Alizadeh et al. [21] | ↓ | - | - | - | - | - | - | ↑ | - | - | - | - | - | - |
| Alvarenga et al. [23] | ↓ | - | - | - | - | - | - | - | - | ↓ | - | - | - | - |
| Asghari et al. [26] | ↓ | - | - | - | - | - | - | ↑ | - | - | - | - | - | - |
| Boshagh et al. [31] | - | - | - | - | - | - | - | ↑ | - | - | - | - | - | - |
| Darmian et al. [37] | ↓ | - | - | ↑ | - | - | - | ↑ | - | - | - | - | - | - |
| Ghazimoradi et al. [46] | - | - | - | - | - | - | - | - | - | - | ↑ | - | - | - |
| Heshmati et al. [51] | - | ↑ | ↑ | - | - | - | - | - | - | - | - | - | - | - |
| Hondaei et al. [53] | ↔ | - | - | - | - | - | - | ↔ | - | - | - | - | - | - |
| Jarhahzaden et al. [57] | ↓ | - | - | - | - | - | - | - | - | - | - | - | - | - |
| Jimenez-Osorio et al. [60] | ↑ | ↓ | ↓ | ↔ | ↓ | ↓ | - | ↓ | - | - | - | ↓ | ↓ | - |
| Krishnareddy et al. [68] | - | ↑ | ↑ | ↑ | - | - | ↓ | - | - | - | - | - | - | - |
| Mamsharifi et al. [70] | ↓ | - | - | ↑ | - | - | - | ↑ | - | - | - | - | - | ↓ |
| Mirhafez et al. [72] | - | - | - | - | - | - | - | - | - | - | ↓ | - | - | - |
| Mokhtari et al. [79] | ↔ | - | - | ↑ | - | - | - | ↑ | - | - | - | - | - | - |
| Osali [83] | ↓ | - | - | - | - | - | - | ↑ | - | - | - | - | - | - |
| Panahi et al. [85] | ↓ | ↑ | - | - | - | - | - | - | - | - | - | - | - | - |
| Panahi et al. [86] | ↓ | ↑ | - | ↑ | - | - | - | - | - | - | - | - | - | - |
| Panahi et al. [87] | ↓ | ↑ | - | - | - | - | - | ↑ | - | - | - | - | - | - |
| Saraf-Bank et al. [107] | ↓ | - | - | - | - | - | - | ↑ | - | - | - | - | - | - |
| Shafabakhsh et al. [110] | ↔ | - | - | ↓ | - | - | - | ↑ | - | - | - | - | - | - |
| Yaikwawong et al. [119] | ↓ | - | - | - | - | - | - | - | ↑ | - | - | - | - | - |

↑=Value increase in the treatment group compared to control group, ↓=Value decrease in the treatment group compared to control group, ↔ = Value approximately or exactly equal between treatment group and control group, MDA=Malondialdehyde, SOD=Superoxide dismutase, GPx= Glutathione peroxidase, GSH= Glutathione, GSSG= Glutathione disulfide, GR= Glutathione reductase, TBARS=Thiobarbituric acid reactive substances, TAC=Total antioxidant capacity, TAS =Total antioxidant status, LDL-ox=Oxidized Low-Density Lipoprotein, PAB=Pro-oxidant Antioxidant Balance, CAT=Catalase activity, FRSA=Free radical scavenging activity, NO=Nitric oxide.

**Supplementary Table S13**

**Evaluating the effects of curcumin consumption on oxidative stress; Glutathione (GSH), Glutathione disulfide (GSSG), Glutathione reductase (GR), Malondialdehyde (MDA), Superoxide dismutase (SOD), Glutathione peroxidase (GPx) and Catalase (CAT) in the clinical studies on metabolic syndrome.**

| Author, Year | GSH (µmol/L) | GSSG (nM) | GR (U/mL) | MDA (µmol/L) | SOD (U/mL) | GPx (U/L) | CAT (U/mg) |
|---|---|---|---|---|---|---|---|
| Alizadeh et al. [21] | | | | | | | |
| Treatment | | | | 0.73±0.07 | | | |
| Placebo | | | | 1.08±0.1 | | | |
| Alvarenga et al. [23] | | | | | | | |
| Treatment | | | | 1.08 (0.25-4.41) ** | | | |
| Placebo | | | | 1.3 (0.10-2.5) ** | | | |
| Asghari et al. [26] | | | | | | | |
| Curcumin group | | | | 5.08±2.93 | | | |
| Placebo | | | | 6.28±1.92 | | | |
| Darmian et al. [37] | | | | | | | |
| Tumeric group | 460.18±24.29 | | | 2.23±0.17 | | | |
| Placebo | 409.12±5.14 | | | 2.41±0.11 | | | |
| Heshmati et al. [51] | | | | | | | |
| Treatment | | | | | 241.02±81.01 | 141.66±64.57 | |
| Placebo | | | | | 205.4±92.02 | 109.37±43.32 | |
| Hondaei et al. [53] | | | | | | | |
| Treatment | | | | 8±1.7 | | | |
| Placebo | | | | 8±1.7 | | | |
| Jarhahzaden et al. [57] | | | | | | | |
| Treatment | | | | 0.167±0.1 mmol/L | | | |
| Placebo | | | | 0.189±0.13 mmol/L | | | |
| Jimenez-Osorio et al. [60] | | | | | | | |
| Treatment | 3.11±1.12 nM | 0.67±0.36 | 0.11±0.36 | 3.09±0.87 nM | 45.71±24.91 | 170±50.99 | 0.14±0.092 |
| Placebo | 3.11±0.98 nM | 0.76±0.45 | 0.13±0.045 | 2.67±0.94 nM | 51.43±12.37 | 210±178.88 | 0.18±0.08 |
| Krishnareddy et al. [68] | | | | | | | |
| Treatment | 5.75±0.75 protein | | | | 7.4±1 IU/L | 9.57±1 | |
| Placebo | 4.08±0.92 protein | | | | 4.8±0.78 IU/L | 6±1.28 | |
| Mamsharifi et al. [70] | | | | | | | |
| Treatment | 572.89±66.03 | | | 3.47±1.53 | | | |
| Placebo | 549.32±75.01 | | | 3.96±2.34 | | | |
| Mokhtari et al. [79] | | | | | | | |
| Treatment | 948.8±350.5 | | | 2.4±0.2 | | | |
| Placebo | 736.1±230.1 | | | 2.4±0.3 | | | |
| Osali [83] | | | | | | | |
| Curcumin group | | | | 1.32±0.78 nmol/dL | | | |
| Placebo | | | | 2.81±0.8 nmol/dL | | | |
| Panahi et al. [84] | | | | | | | |
| Treatment | | | | 15.62±2.59 | 2.41±0.37 | | |

| | | | |
|---|---|---|---|
| Placebo | | 20.16±3.11 | 1.98±0.29 |
| Panahi et al. [85] | | | |
| Treatment | 124.76±67.45 | 17.78±4.84 | 7±3.49 |
| Placebo | 92.57±43.48 | 20.56±3.67 | 4±1.83 |
| Panahi et al. [87] | | | |
| Treatment | | 3.05±0.91 | 3.86±0.76 |
| Placebo | | 3.88±0.98 | 2.96±0.78 |
| Saraf-Bank et al. [107] | | | |
| Treatment | | 73.78±51.5 | |
| Placebo | | 103.23±39.55 | |
| Yaikwawong et al. [118] | | | |
| Treatment | | 1.45±0.33 | |
| Placebo | | 2.4±0.87 | |

GSH=Glutathione, GSSG=Glutathione disulfide, GR=Glutathione Reductase, MDA=Malondialdehyde, SOD=Superoxide dismutase, GPx=Glutathione peroxidase, CAT=Catalase activity **=Median Value.

**Supplementary Table S14**
**Evaluating the effects of curcumin consumption on oxidative stress, LDL oxidase (LDL-ox), Pro-oxidant Antioxidant Balance (PAB), Free radical scavenging activity (FRSA), Nitric oxide (NO), Thiobarbituric acid reactive substances (TBARS), Total antioxidant capacity (TAC), and Total antioxidant status (TAS) in the clinical studies on metabolic syndrome.**

| Author, Year | LDL-ox (pg/mL) | PAB (HK) | FRSA (%) | NO (μmol/L) | TBARS (nmol/mL) | TAC (mmol/L) | TAS (μmol Trolox eq/L) |
|---|---|---|---|---|---|---|---|
| Alizadeh et al. [21] | | | | | | | |
| Treatment | | | | | | 1.95±0.05 μmol/l | |
| Placebo | | | | | | 1.35±0.09 μmol/l | |
| Alvarenga et al. [23] | | | | | | | |
| Treatment | 967± 301 | | | | | | |
| Placebo | 1058± 518 | | | | | | |
| Asghari et al. [26] | | | | | | | |
| Curcumin group | | | | | | 3.54±1.32 | |
| Placebo | | | | | | 3.17±1.22 | |
| Boshagh et al. [31] | | | | | | | |
| Treatment | | | | | | 2.88±0.19 | |
| Placebo | | | | | | 2.84±0.19 | |
| Darmian et al. [37] | | | | | | | |
| Tumeric group | | | | | | 860.24±14.06 | |
| Placebo | | | | | | 827.14±6.04 | |
| Ghazimoradi et al. [46] | | | | | | | |
| Phospholipid Curcumin | | 98.32** | | | | | |
| Curcumin group | | 142.14** | | | | | |
| Placebo | | 110.43** | | | | | |
| Hondaei et al. [53] | | | | | | | |
| Treatment | | | | | | 0.8±0.1 U/ml | |
| Placebo | | | | | | 0.8±0.1 U/ml | |
| Jimenez-Osorio et al. [60] | | | | | | | |
| Treatment | | | 16.13±3.82 | | | 3300±713.86 μM | |
| Placebo | | | 18.13±2.68 | | | 3600±715.52 μM | |
| Krishnareddy et al. [68] | | | | | | | |
| Treatment | | | | | 38.33±4.46 | | |
| Placebo | | | | | 56.25±6.25 | | |
| Mamsharifi et al. [70] | | | | | | | |
| Treatment | | | | 42.23±7.23 | | 733.05±185.14 | |
| Placebo | | | | 45.49±8.06 | | 699.85±187.54 | |
| Mirhafez et al. [72] | | | | | | | |
| Treatment | | 82.65±27.64 | | | | | |
| Placebo | | 93.94±31.61 | | | | | |
| Mokhtari et al. [79] | | | | | | | |
| Treatment | | | | | | 1499.1±249.1 | |

| | | |
|---|---|---|
| Placebo | 1263.4±229.5 | |
| Osali [83] | | |
| Curcumin group | 1.31±0.78 | |
| Placebo | 0.521±0.8 | |
| Panahi et al. [87] | | |
| Treatment | 3.84 nmol/mg** | |
| Placebo | 2.63 nmol/mg** | |
| Saraf-Bank et al. [107] | | |
| Treatment | 0.23±0.04 | |
| Placebo | 0.22±0.03 | |
| Shafabakhsh et al. [110] | | |
| Treatment | 904.4±185.6 | |
| Placebo | 869.8±174.6 | |
| Saraf-Bank et al. [107] | | |
| Treatment | 0.23±0.04 | |
| Placebo | 0.22±0.03 | |
| Yaikwawong et al. [118] | | |
| Treatment | | 1.86±0.22 |
| Placebo | | 1.63±0.25 |

LDL-ox=LDL oxidase, PAB=Pro-oxidant Antioxidant Balance, FRSA=Free radical scavenging activity, NO=Nitric oxide, TBARS=Thiobarbituric acid reactive substances, TAC=Total antioxidant capacity, TAS=Total antioxidant status, **=Median Value

| Study | Experimental Mean | Experimental SD | Experimental Total | Control Mean | Control SD | Control Total | Weight | Std. Mean Difference IV, Random, 95% CI |
|---|---|---|---|---|---|---|---|---|
| Asadi et al. 2019 | 31.01 | 4.1000 | 40 | 30.80 | 3.7000 | 40 | 8.8% | 0.05 [-0.39; 0.49] |
| Funamoto et al. 2019 | 25.00 | 4.5000 | 15 | 24.80 | 2.7000 | 18 | 6.8% | 0.05 [-0.63; 0.74] |
| Hondaei et al. 2019 | 28.90 | 3.7300 | 21 | 28.10 | 2.5000 | 23 | 7.5% | 0.25 [-0.34; 0.84] |
| Jimenez-Osorio et al. 2016 | 29.90 | 6.3500 | 28 | 26.80 | 8.6300 | 23 | 7.8% | 0.41 [-0.15; 0.97] |
| Mokhtari et al. 2020 | 27.30 | 4.7000 | 25 | 29.90 | 5.2000 | 25 | 7.8% | -0.52 [-1.08; 0.05] |
| Na et al. 2012 | 26.32 | 2.8200 | 50 | 26.77 | 3.7300 | 50 | 9.1% | -0.14 [-0.53; 0.26] |
| Neta et al. 2021 | 29.50 | 4.6000 | 33 | 28.60 | 4.8000 | 28 | 8.2% | 0.19 [-0.32; 0.69] |
| Panahi et al. 2016 | 26.04 | 2.3500 | . | 27.57 | 1.6300 | . | 0.0% | |
| Panahi et al. 2017 | 26.04 | 2.3500 | 50 | 27.57 | 1.6300 | 50 | 9.0% | -0.75 [-1.16; -0.34] |
| Panahi et al. 2018 | 26.00 | 2.0000 | 50 | 28.00 | 1.0000 | 50 | 8.8% | -1.26 [-1.69; -0.82] |
| Rahimi et al. 2016 | 25.57 | 2.7100 | 35 | 27.50 | 3.3800 | 35 | 8.4% | -0.62 [-1.10; -0.14] |
| Shafabakhsh et al. 2020 | 30.30 | 5.9000 | 25 | 30.00 | 3.0000 | 24 | 7.8% | 0.06 [-0.50; 0.62] |
| Yaikwawong et al. 2024 | 25.94 | 4.8400 | 113 | 29.34 | 4.8500 | 114 | 10.0% | -0.70 [-0.97; -0.43] |
| **Total (95% CI)** | | | **485** | | | **480** | **100.0%** | **-0.27 [-0.57; 0.02]** |
| **Prediction interval** | | | | | | | | **[-1.32; 0.77]** |

Heterogeneity: $Tau^2 = 0.2040$; $Chi^2 = 51.84$, df = 11 ($P < 0.0001$); $I^2 = 78.8\%$
Test for overall effect: Z = -1.83 (P = 0.0667)

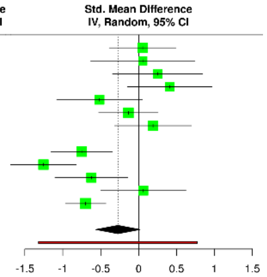


**A**

| Study | Experimental Mean | Experimental SD | Experimental Total | Control Mean | Control SD | Control Total | Weight | Std. Mean Difference IV, Random, 95% CI |
|---|---|---|---|---|---|---|---|---|
| Bateni et al. 2021 | 29.80 | 4.5000 | 22 | 29.10 | 4.1000 | 21 | 13.3% | 0.16 [-0.44; 0.76] |
| Mohammadi et al. 2017 | 30.36 | 3.8000 | 36 | 31.30 | 4.8700 | 36 | 16.4% | -0.21 [-0.68; 0.25] |
| Osali et al. 2020 | 29.66 | 2.6000 | 11 | 29.11 | 1.5100 | 11 | 9.2% | 0.25 [-0.59; 1.09] |
| Rahmani et al. 2016 | 30.11 | 4.3900 | 37 | 31.37 | 5.3300 | 40 | 16.7% | -0.25 [-0.70; 0.19] |
| Sangouni et al. 2022 | 29.40 | 4.2000 | 22 | 29.70 | 4.7000 | 22 | 13.5% | -0.07 [-0.66; 0.53] |
| Shirmohammadi et al. 2019 | 31.03 | 5.1100 | 37 | 31.30 | 4.8700 | 36 | 16.5% | -0.05 [-0.51; 0.41] |
| Yang et al. 2014 | 33.70 | 5.1500 | 30 | 28.88 | 4.8800 | 29 | 14.6% | 0.95 [ 0.41; 1.49] |
| **Total (95% CI)** | | | **195** | | | **195** | **100.0%** | **0.09 [-0.23; 0.41]** |
| **Prediction interval** | | | | | | | | **[-0.81; 0.98]** |

Heterogeneity: $Tau^2 = 0.1064$; $Chi^2 = 14.30$, df = 6 (P = 0.0265); $I^2 = 58.0\%$
Test for overall effect: Z = 0.53 (P = 0.5938)

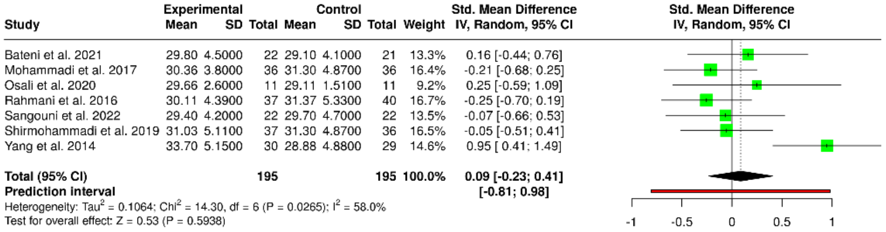


**B**

| Study | Experimental Mean | Experimental SD | Experimental Total | Control Mean | Control SD | Control Total | Weight | Std. Mean Difference IV, Random, 95% CI |
|---|---|---|---|---|---|---|---|---|
| Chashmniam et al. 2019 | 29.60 | 3.4500 | 25 | 27.72 | 4.2500 | 20 | 7.9% | 0.48 [-0.11; 1.08] |
| Ghaffari et al. 2019 | 31.50 | 4.7300 | 21 | 32.80 | 4.9400 | 21 | 7.6% | -0.26 [-0.87; 0.34] |
| Hariri et al. 2020 | 29.83 | 6.0300 | 23 | 28.75 | 3.4700 | 22 | 8.2% | 0.21 [-0.37; 0.80] |
| Jazayeri-Tehrani et al. 2019 | 29.70 | 2.1000 | 42 | 29.90 | 2.5300 | 42 | 15.3% | -0.09 [-0.51; 0.34] |
| Kelardeh et al. 2020 | 27.48 | 1.3000 | 11 | 27.21 | 1.2900 | 11 | 4.0% | 0.20 [-0.64; 1.04] |
| Mirhafez et al. 2021 | 29.10 | 3.8000 | 35 | 28.60 | 3.8000 | 37 | 13.1% | 0.13 [-0.33; 0.59] |
| Mirhafez et al.a 2019 | 29.75 | 5.8400 | 32 | 28.78 | 4.2200 | 29 | 11.1% | 0.19 [-0.32; 0.69] |
| Mirhafez et al.b 2019 | 28.00 | 9.1300 | 22 | 29.97 | 6.7200 | 22 | 8.0% | -0.24 [-0.83; 0.35] |
| Panahi et al. 2017 | 27.98 | 3.0500 | 44 | 28.92 | 3.6900 | 43 | 15.7% | -0.28 [-0.70; 0.15] |
| Saadati et al. 2019 | 31.44 | 5.0200 | 27 | 31.33 | 6.0800 | 23 | 9.1% | 0.02 [-0.54; 0.58] |
| **Total (95% CI)** | | | **282** | | | **270** | **100.0%** | **0.01 [-0.16; 0.17]** |
| **Prediction interval** | | | | | | | | **[-0.19; 0.20]** |

Heterogeneity: $Tau^2 = 0$; $Chi^2 = 7.22$, df = 9 (P = 0.6141); $I^2 = 0.0\%$
Test for overall effect: Z = 0.09 (P = 0.9318)

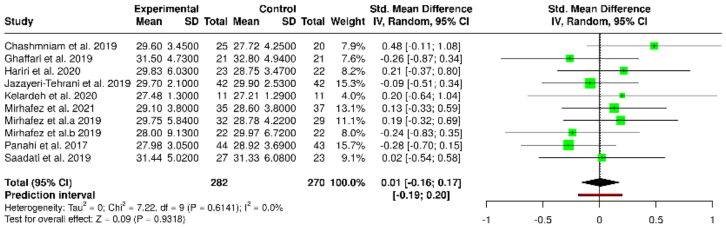


**C**

| Study | Experimental Mean | Experimental SD | Experimental Total | Control Mean | Control SD | Control Total | Weight | Std. Mean Difference IV, Random, 95% CI |
|---|---|---|---|---|---|---|---|---|
| Atakan et al. 2022 | 29.38 | 5.4100 | 35 | 29.88 | 5.0400 | 35 | 14.0% | -0.09 [-0.56; 0.37] |
| Cicero et al. 2019 | 26.30 | 1.4000 | 40 | 26.40 | 1.7000 | 40 | 14.3% | -0.06 [-0.50; 0.37] |
| Dolati et al. 2020 | 26.59 | 2.0600 | 10 | 26.89 | 1.5600 | 10 | 10.4% | -0.16 [-1.04; 0.72] |
| Farzad et al. 2020 | 26.51 | 1.8000 | 10 | 26.80 | 1.5000 | 10 | 10.4% | -0.17 [-1.05; 0.71] |
| Karandish et al. 2021 | 28.78 | 2.8600 | 21 | 30.05 | 2.6500 | 20 | 12.7% | -0.45 [-1.07; 0.17] |
| Majeed et al. 2023 | 29.44 | 2.3000 | 47 | 30.79 | 1.9100 | 47 | 14.5% | -0.63 [-1.05; -0.22] |
| Porasgari et al. 2022 | 38.87 | 5.4300 | 14 | 29.03 | 4.6500 | 13 | 10.0% | 1.88 [ 0.95; 2.81] |
| Saraf-Bank et al. 2019 | 31.00 | 2.8500 | 30 | 30.00 | 2.8200 | 30 | 13.7% | 0.35 [-0.16; 0.86] |
| **Total (95% CI)** | | | **207** | | | **205** | **100.0%** | **0.03 [-0.44; 0.50]** |
| **Prediction interval** | | | | | | | | **[-1.49; 1.55]** |

Heterogeneity: $Tau^2 = 0.3533$; $Chi^2 = 28.11$, df = 7 (P = 0.0002); $I^2 = 75.1\%$
Test for overall effect: Z = 0.13 (P = 0.9000)

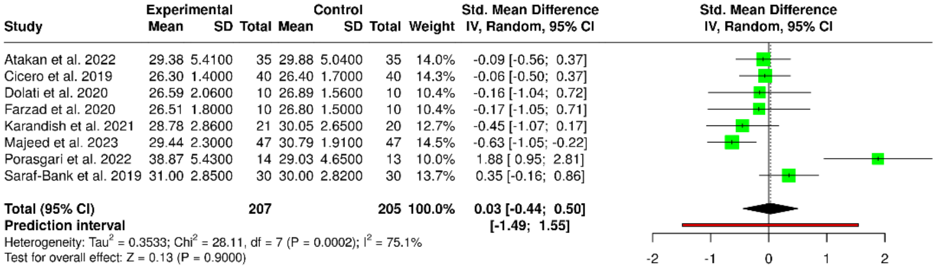


**D**

| Study | Experimental Mean | Experimental SD | Experimental Total | Control Mean | Control SD | Control Total | Weight | Std. Mean Difference IV, Random, 95% CI |
|---|---|---|---|---|---|---|---|---|
| Asan et al. 2020 | 28.10 | 6.7000 | 15 | 29.20 | 4.3000 | 15 | 20.5% | -0.19 [-0.91; 0.53] |
| Heshmati et al. 2020 | 27.71 | 5.0100 | 34 | 26.93 | 4.0300 | 33 | 45.9% | 0.17 [-0.31; 0.65] |
| Zohrabi et al. 2023 | 28.72 | 2.9000 | 25 | 29.26 | 3.3900 | 24 | 33.6% | -0.17 [-0.73; 0.39] |
| **Total (95% CI)** | | | **74** | | | **72** | **100.0%** | **-0.02 [-0.34; 0.31]** |
| **Prediction interval** | | | | | | | | **[-0.73; 0.70]** |

Heterogeneity: $Tau^2 = 0$; $Chi^2 = 1.08$, df = 2 (P = 0.5818); $I^2 = 0.0\%$
Test for overall effect: Z = -0.11 (P = 0.9139)

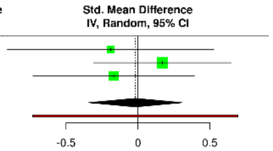


**E**

**Supplementary Figure S1a. Forest plot analysis showing the clinical effects of consuming curcumin/*Curcuma longa* extracts compared to the control group on BMI (Diabetes (A), Mes (B), NAFLD (C), Obese (D), and PCOS (E)) in RCTs.**

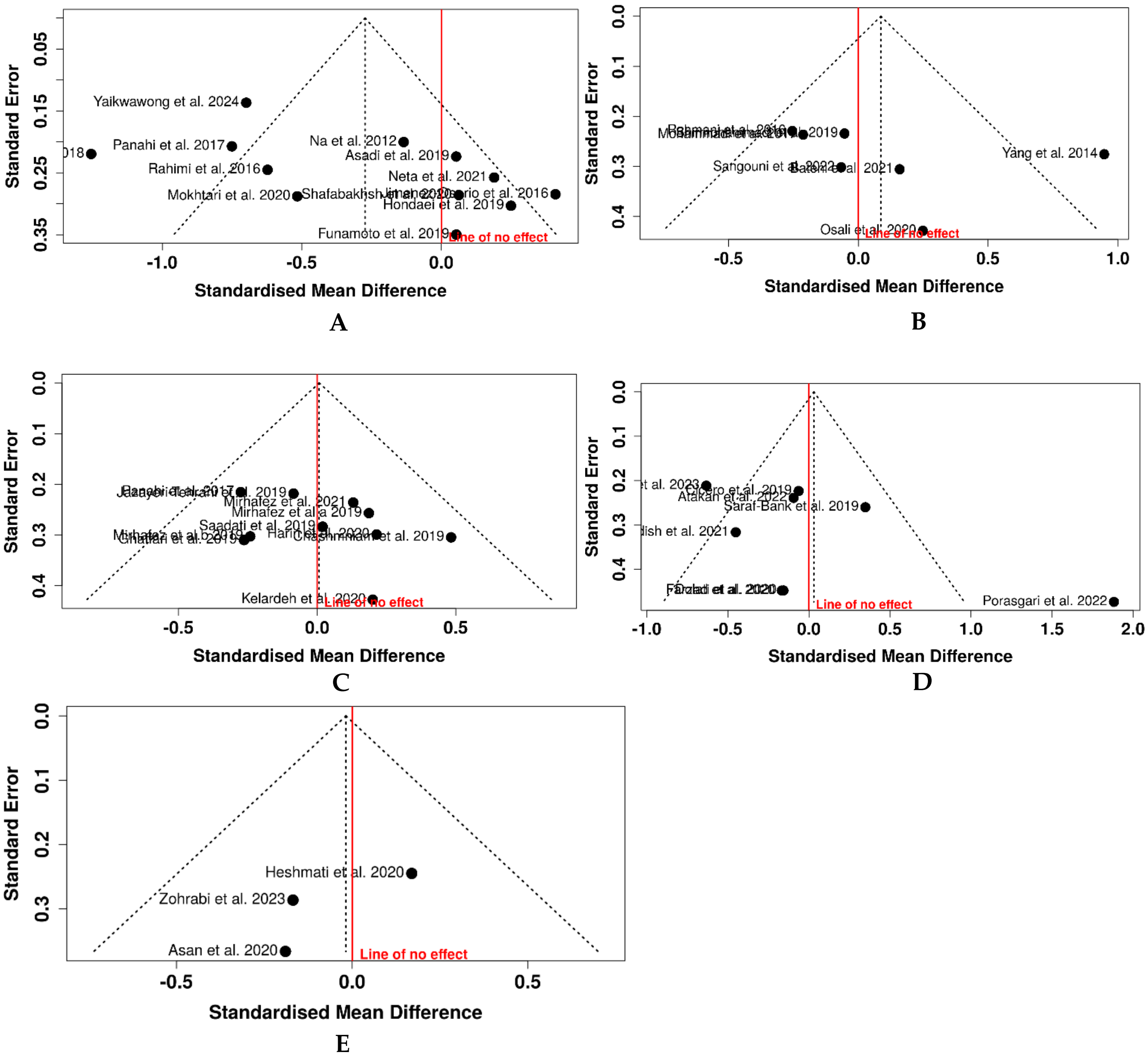


**Supplementary Figure S1b. Funnel plot analysis showing the clinical effects of consuming curcumin/*Curcuma longa* extracts compared to the control group on BMI (Diabetes (A), Mes (B), NAFLD (C), Obese (D), and PCOS (E)) in RCTs.**

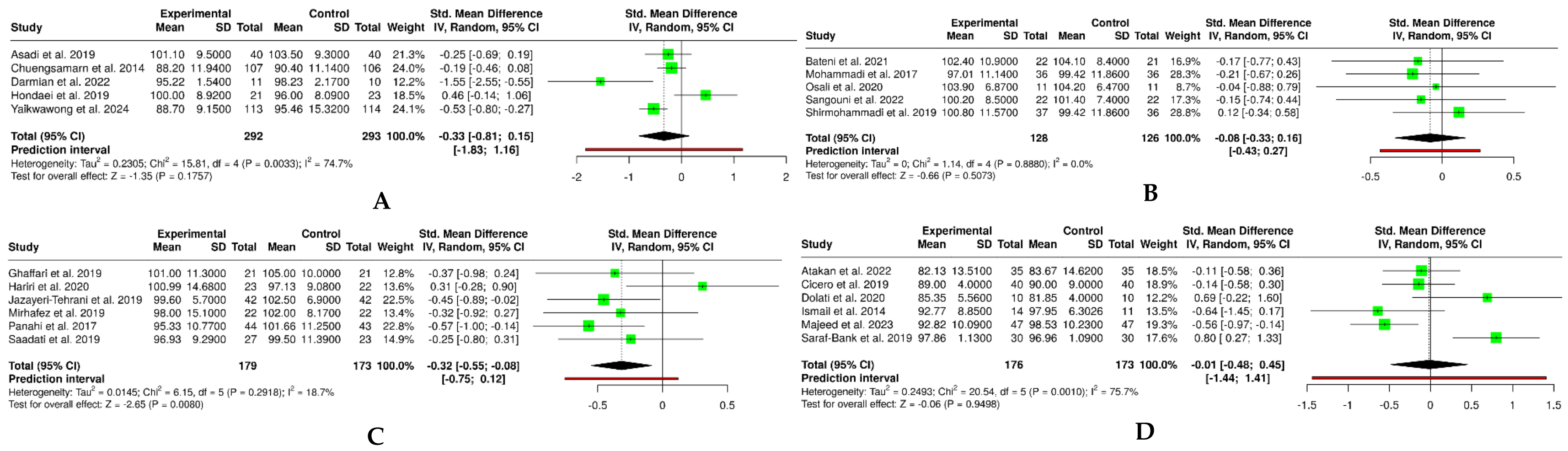


**Supplementary Figure S2a. Forest plot analysis showing the clinical effects of consuming curcumin/*Curcuma longa* extracts compared to the control group on WC (Diabetes (A), Mes (B), NAFLD (C), and Obese (D)) in RCTs.**

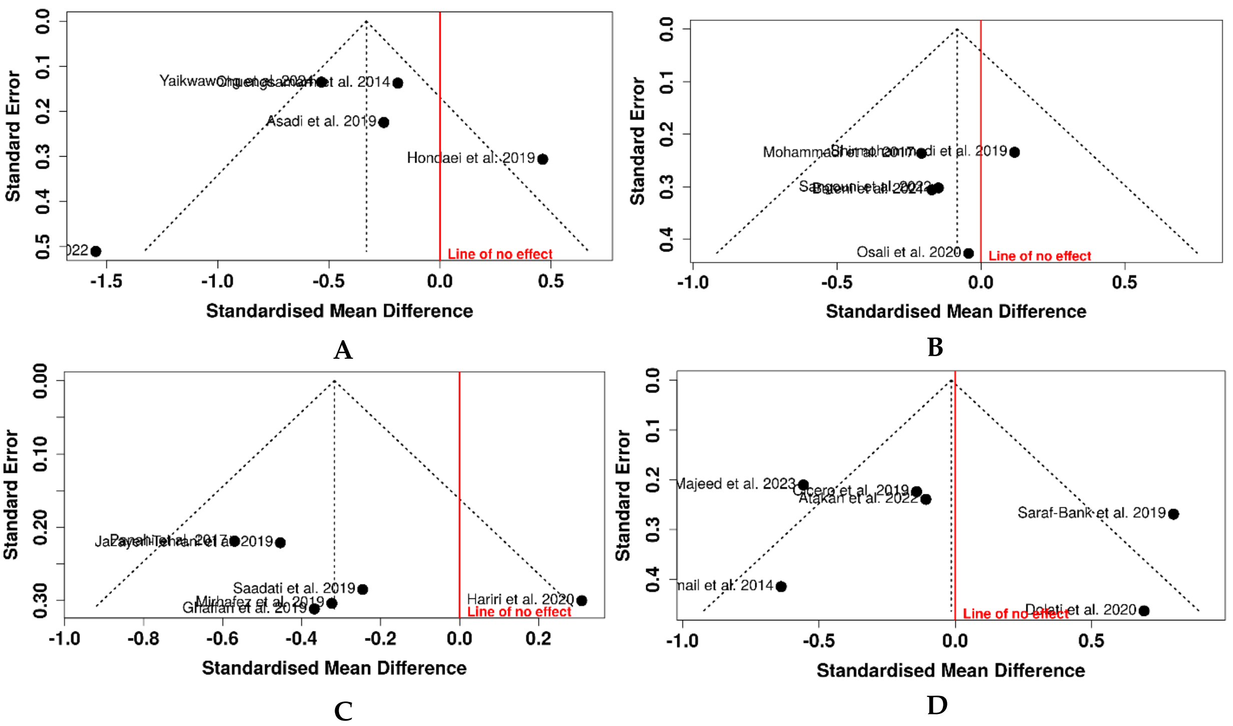


**Supplementary Figure S2b. Funnel plot analysis showing the clinical effects of consuming curcumin/*Curcuma longa* extracts compared to the control group on WC (Diabetes (A), Mes (B), NAFLD (C), and Obese (D)) in RCTs.**

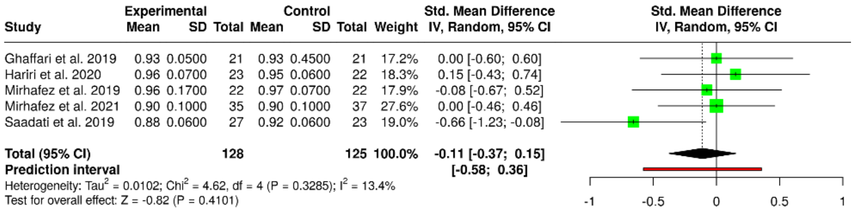


A

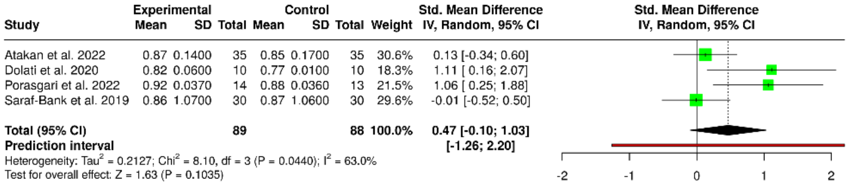


B

**Supplementary Figure S3a. Forest plot analysis showing the clinical effects of consuming curcumin/*Curcuma longa* extracts compared to the control group on WH (NAFLD (A), and Obese (B)) in RCTs.**

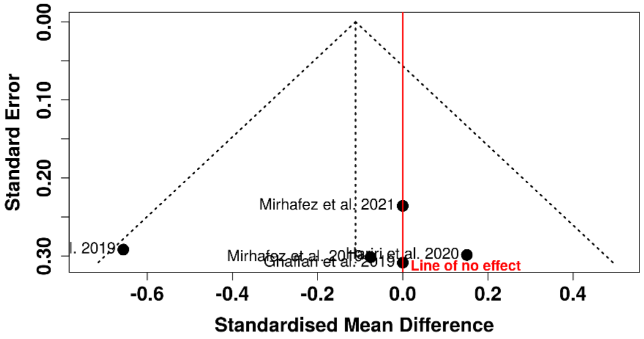


A

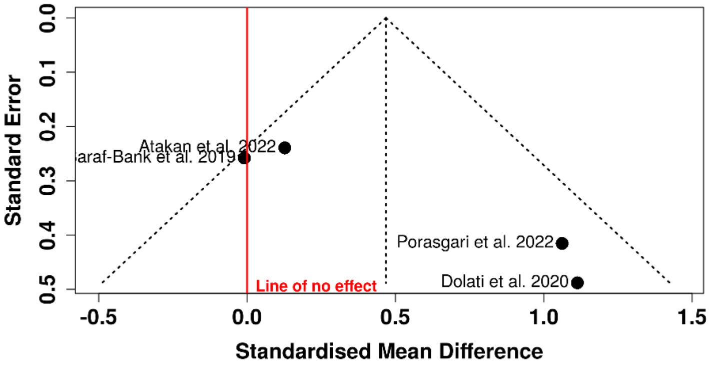


B

**Supplementary Figure S3b. Funnel plot analysis showing the clinical effects of consuming curcumin/*Curcuma longa* extracts compared to the control group on WHR (NAFLD (A), and Obese (B)) in RCTs.**

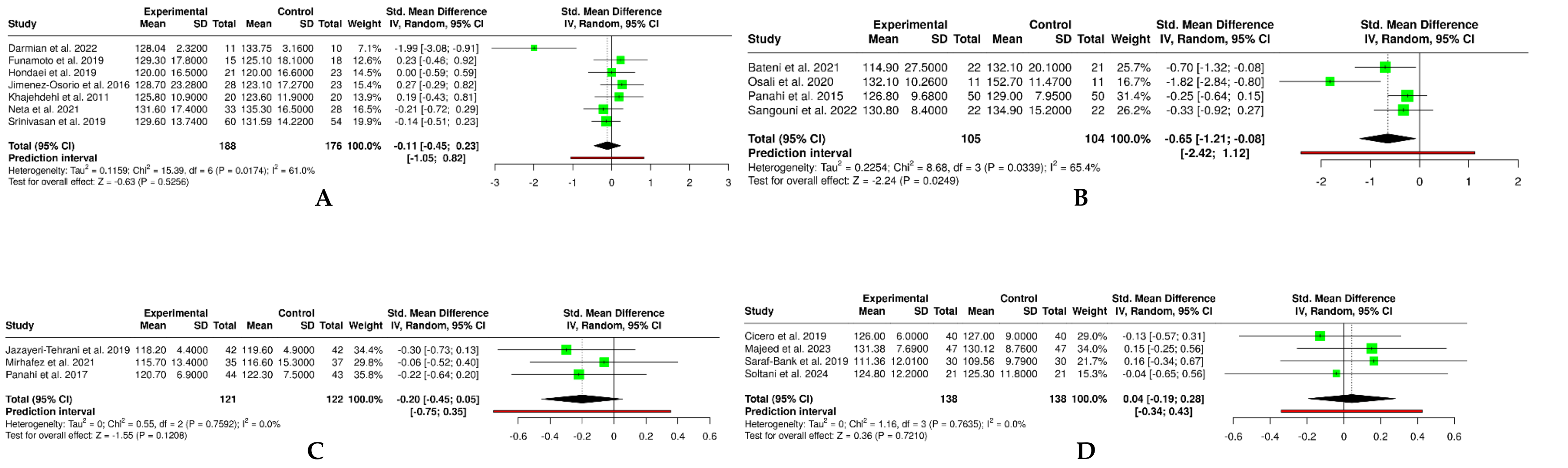


**Supplementary Figure S4a. Forest plot analysis showing the clinical effects of consuming curcumin/*Curcuma longa* extracts compared to the control group on SBP (Diabetes (A), Mes (B), NAFLD (C), and Obese (D)) in RCTs.**

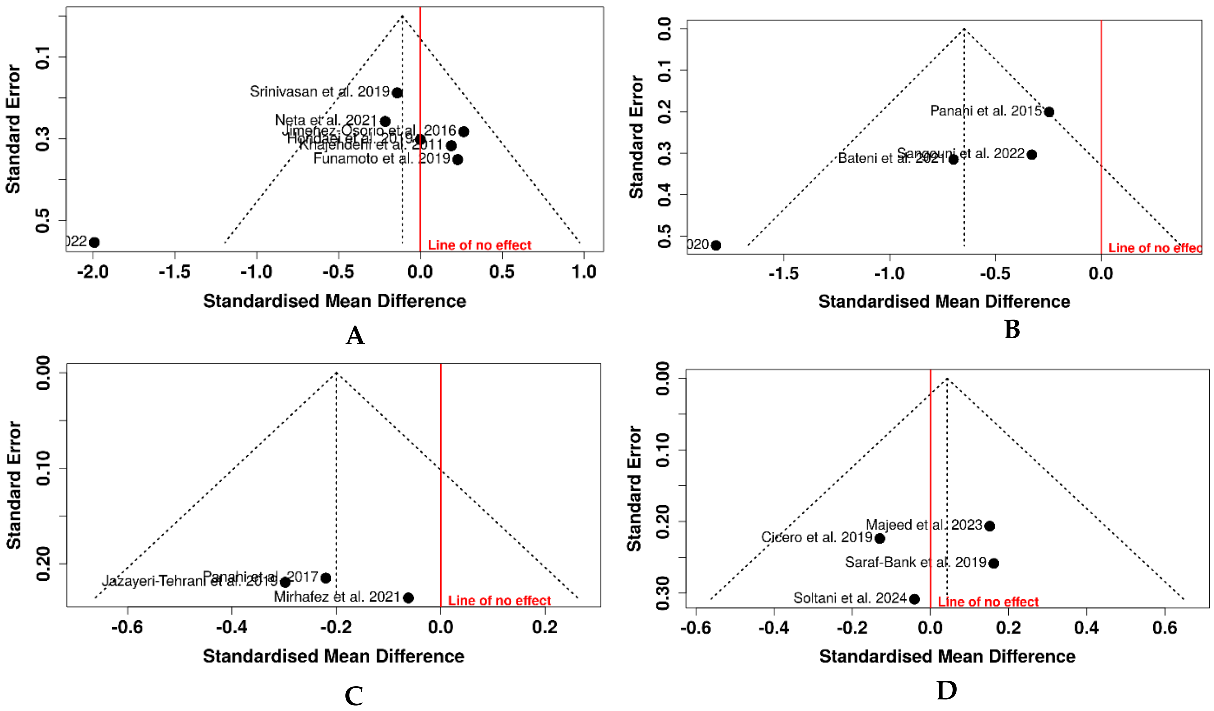


**Supplementary Figure S4b. Funnel plot analysis showing the clinical effects of consuming curcumin/*Curcuma longa* extracts compared to the control group on SBP (Diabetes (A), Mes (B), NAFLD (C), and Obese (D)) in RCTs.**

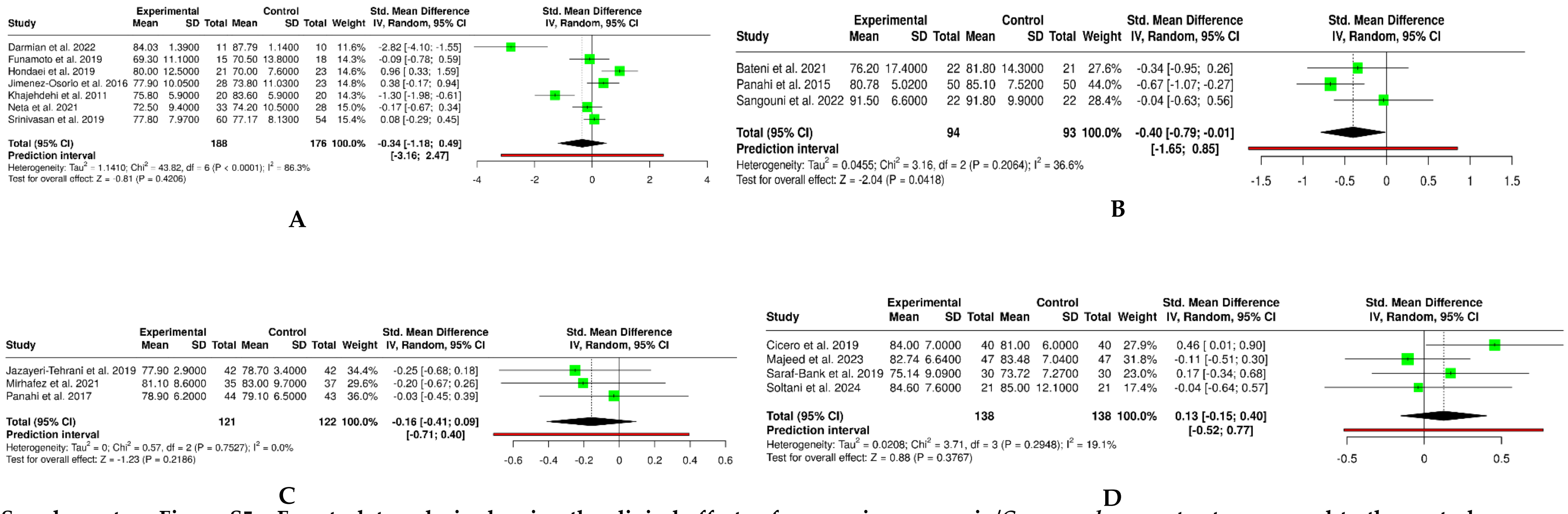


**Supplementary Figure S5a. Forest plot analysis showing the clinical effects of consuming curcumin/*Curcuma longa* extracts compared to the control group on DBP (Diabetes (A), Mes (B), NAFLD (C), and Obese (D)) in RCTs.**

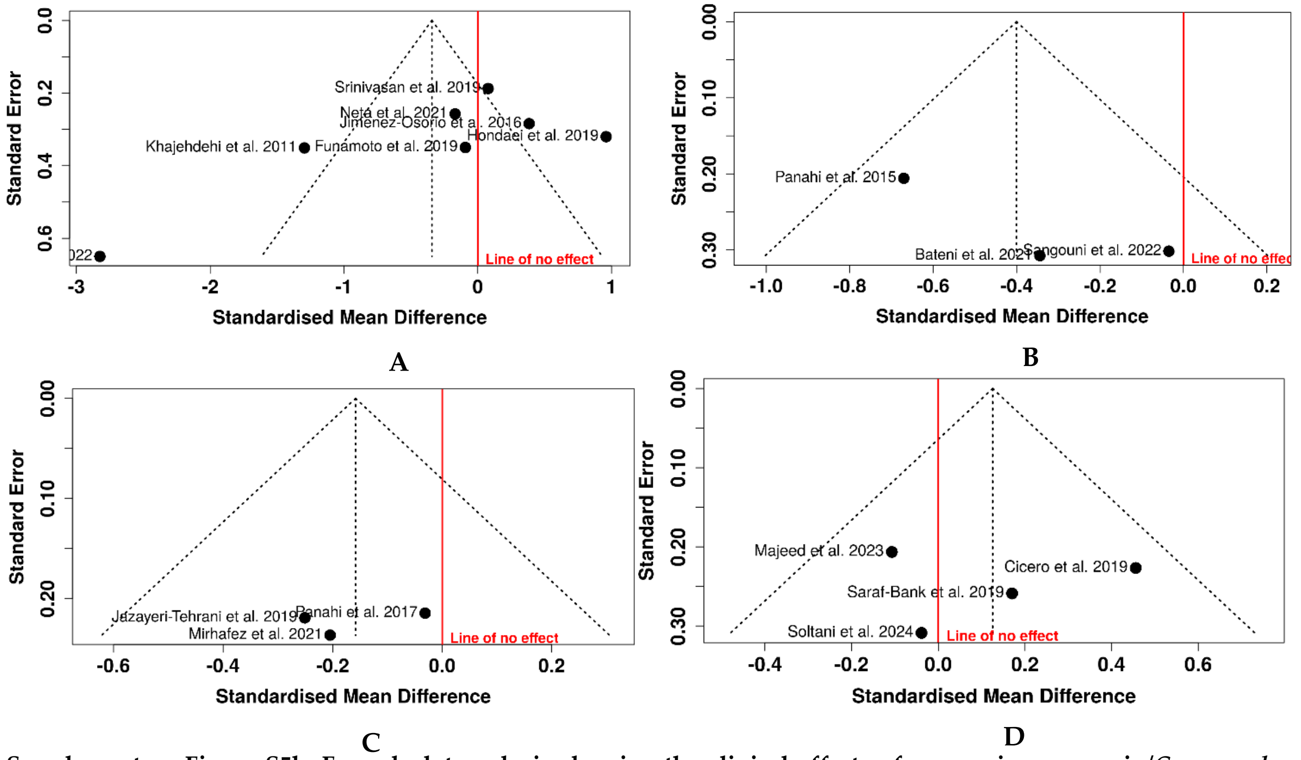


**Supplementary Figure S5b. Funnel plot analysis showing the clinical effects of consuming curcumin/*Curcuma longa* extracts compared to the control group on DBP (Diabetes (A), Mes (B), NAFLD (C), and Obese (D)) in RCTs.**

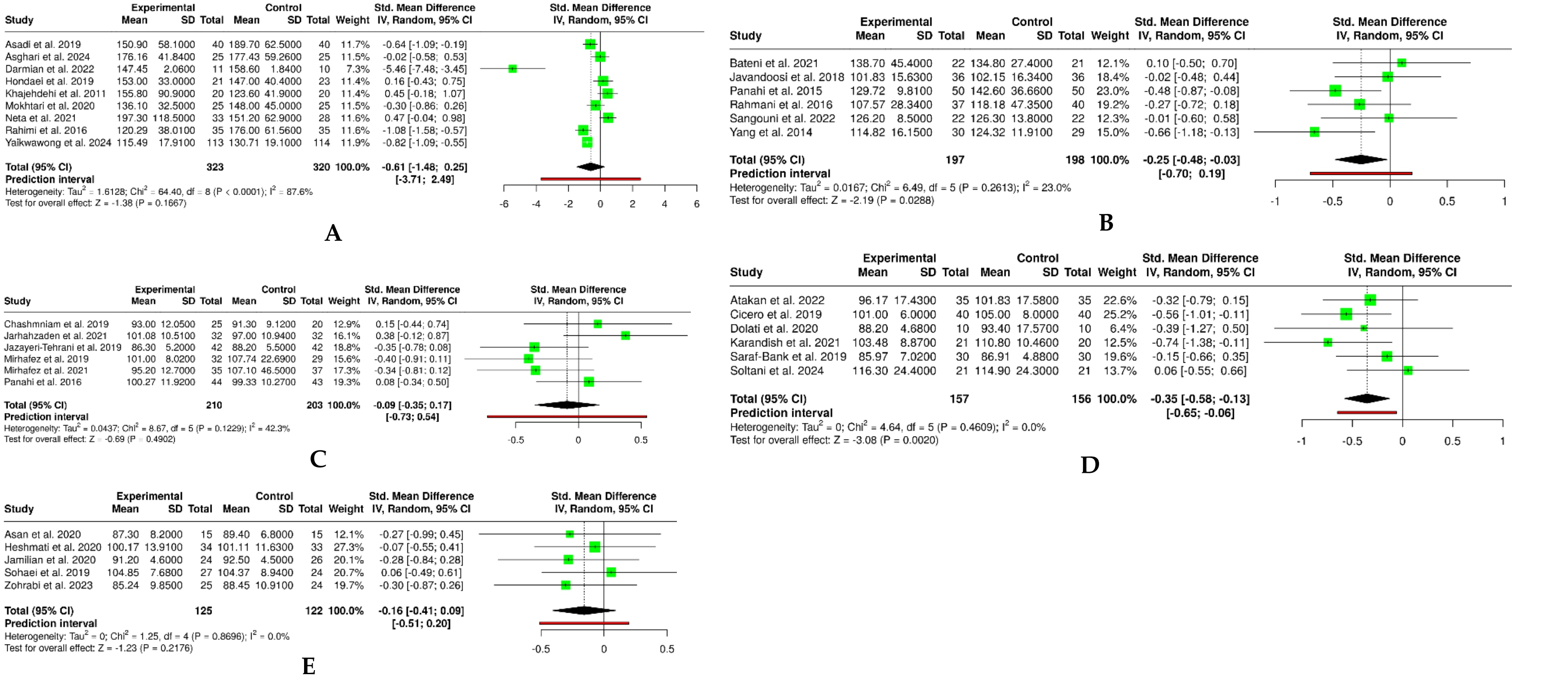


**Supplementary Figure S6a. Forest plot analysis showing the clinical effects of consuming curcumin/*Curcuma longa* extracts compared to the control group on FBS (Diabetes (A), Mes (B), NAFLD (C), Obese (D), and PCOS (E) in RCTs.**

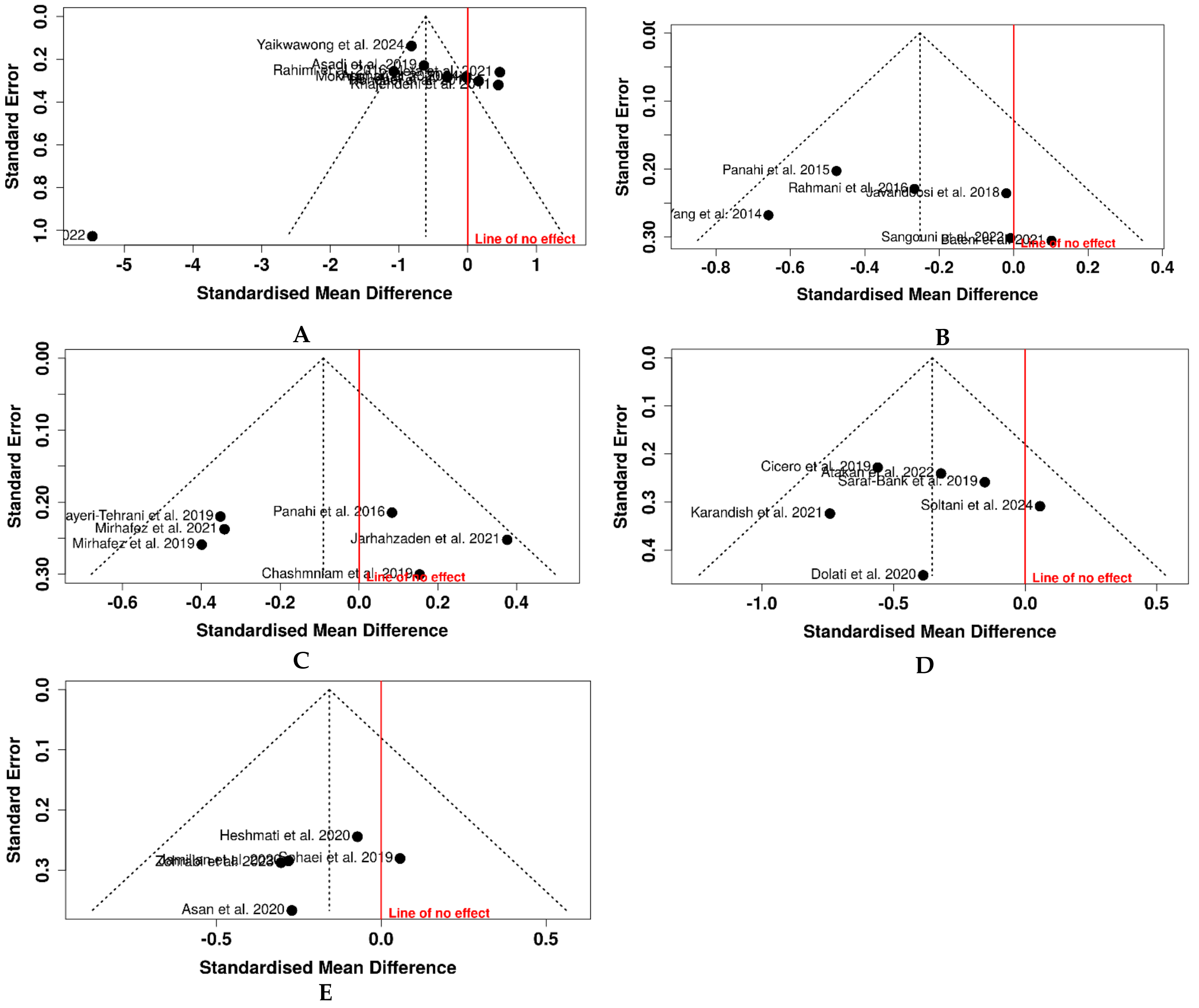


**Supplementary Figure S6b. Funnel plot analysis showing the clinical effects of consuming curcumin/*Curcuma longa* extracts compared to the control group on FBS (Diabetes (A), Mes (B), NAFLD (C), Obese (D), and PCOS (E) in RCTs.**

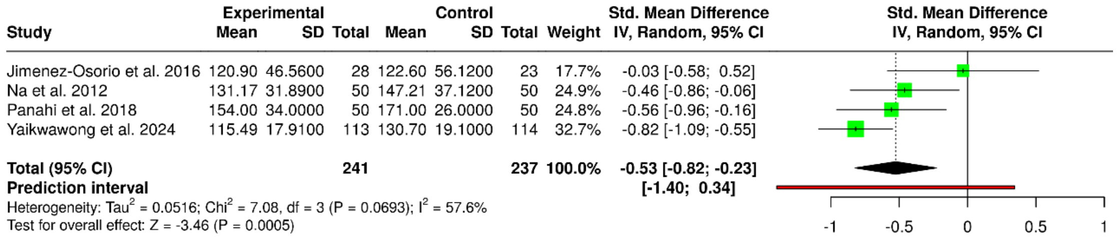


**Supplementary Figure S7a. Forest plot analysis showing the clinical effects of consuming curcumin/*Curcuma longa* extracts compared to the control group on GLU (Diabetes) in RCTs.**

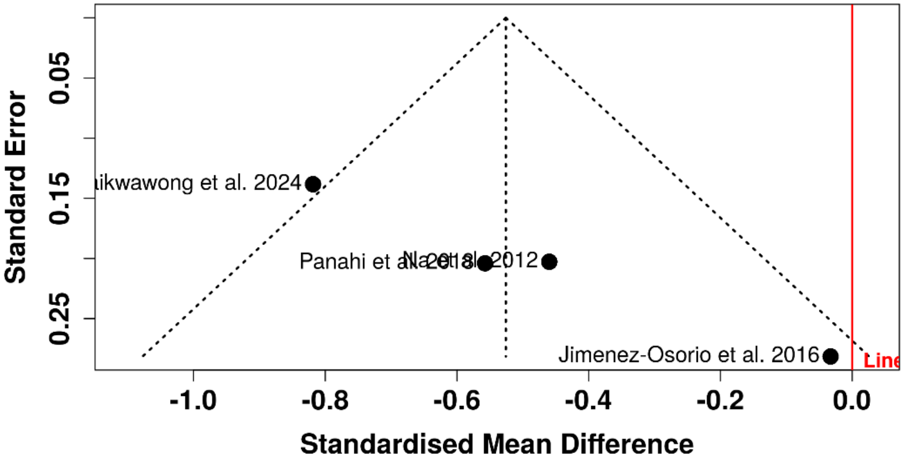


**Supplementary Figure S7b. Funnel plot analysis showing the clinical effects of consuming curcumin/*Curcuma longa* extracts compared to the control group on GLU (Diabetes) in RCTs.**

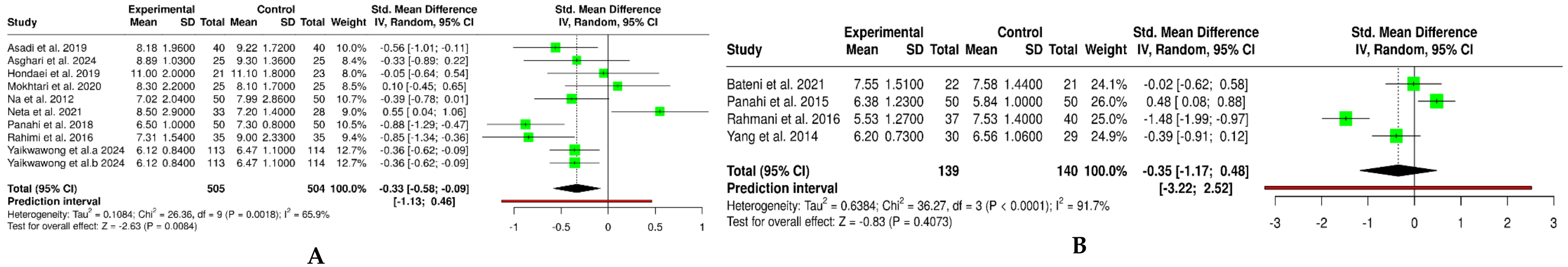


**Supplementary Figure S8a.** Forest plot analysis showing the clinical effects of consuming curcumin/*Curcuma longa* extracts compared to the control group on HbA1c (Diabetes (A), Mes (B)) in RCTs.

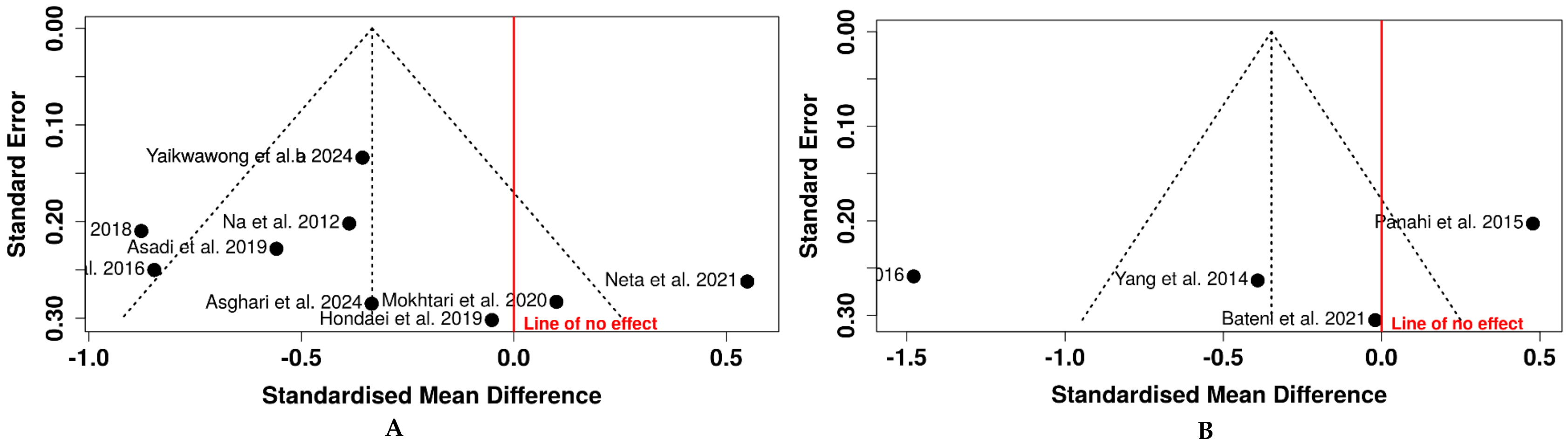


**Supplementary Figure S8b.** Funnel plot analysis showing the clinical effects of consuming curcumin/*Curcuma longa* extracts compared to the control group on HbA1c (Diabetes (A), Mes (B)) in RCTs.

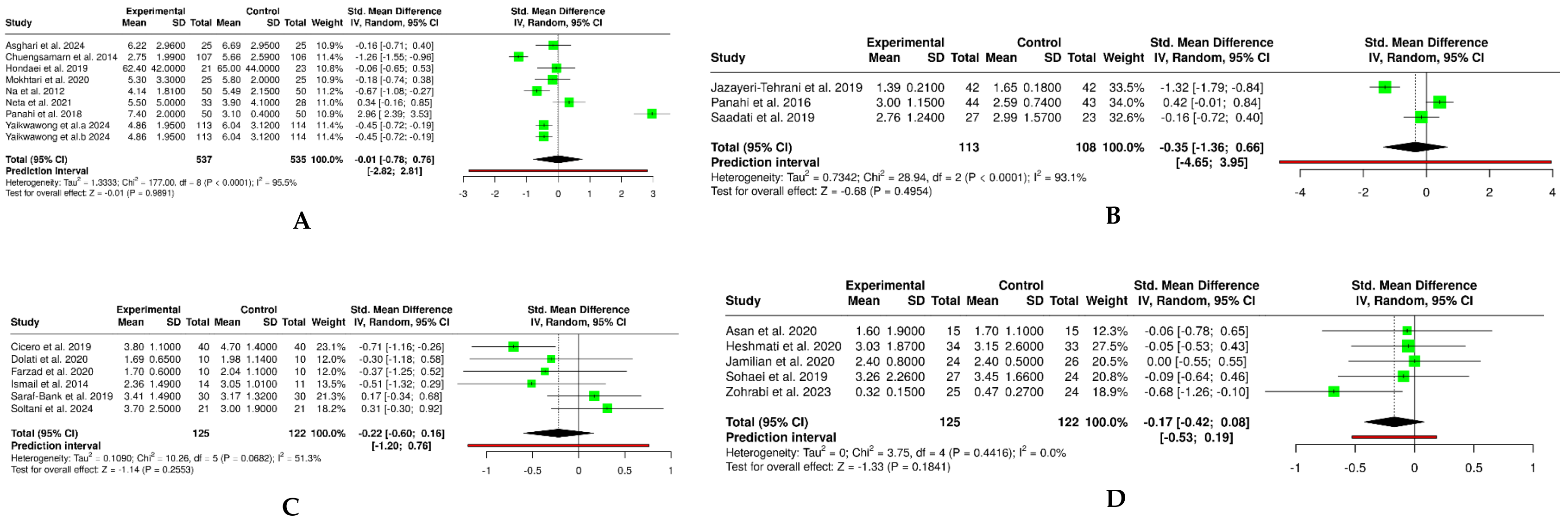


**Supplementary Figure S9a. Forest plot analysis showing the clinical effects of consuming curcumin/*Curcuma longa* extracts compared to the control group on HOMA-IR (Diabetes (A) NAFLD (B), Obese (C) and PCOS (D)) in RCTs.**

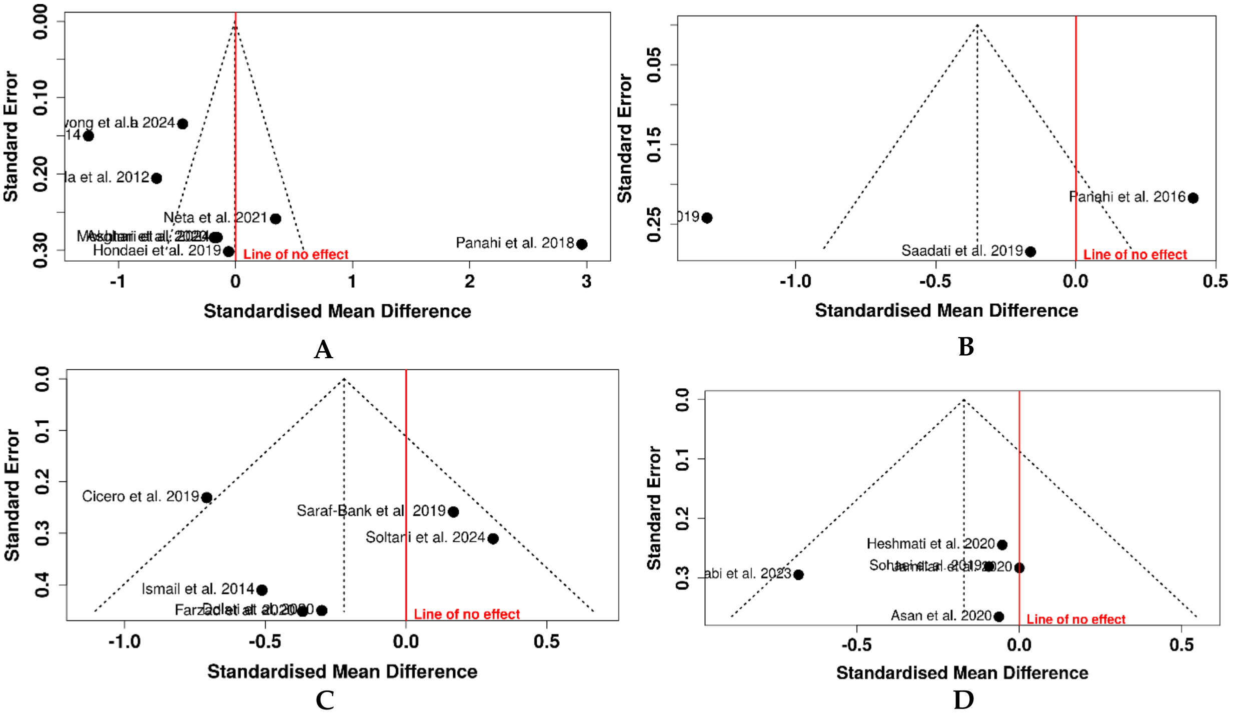


**Supplementary Figure S9b. Funnel plot analysis showing the clinical effects of consuming curcumin/*Curcuma longa* extracts compared to the control group on HOMA-IR (Diabetes (A), NAFLD (B), Obese (C), and PCOS (D)) in RCTs.**

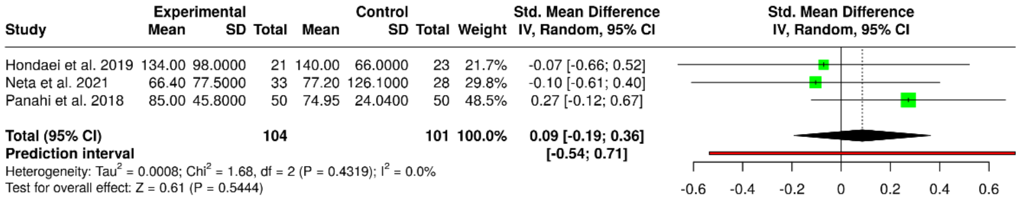


**Supplementary Figure S10a. Forest plot analysis showing the clinical effects of consuming curcumin/*Curcuma longa* extracts compared to the control group on HOMA-B (Diabetes) in RCTs.**

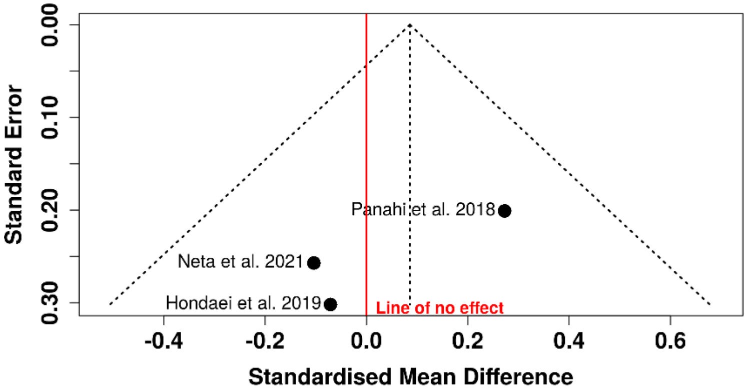


**Supplementary Figure S10b. Funnel plot analysis showing the clinical effects of consuming curcumin/*Curcuma longa* extracts compared to the control group on HOMA-B (Diabetes) in RCTs.**

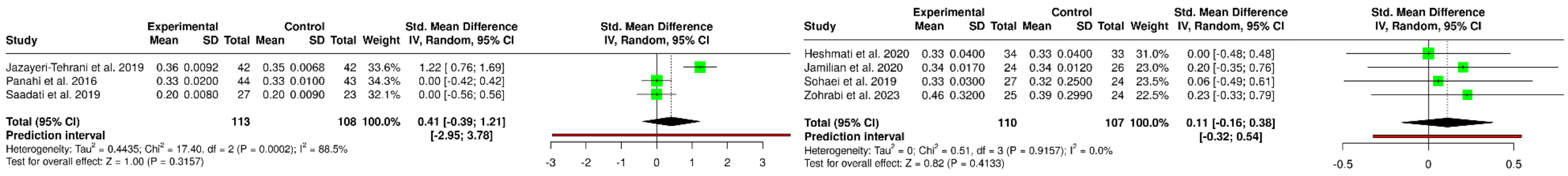


A

B

**Supplementary Figure S11a. Forrest plot analysis showing the clinical effects of consuming curcumin/*Curcuma longa* extracts compared to the control group on QUIKI (NAFLD (A) and PCOS (B)) in RCTs.**

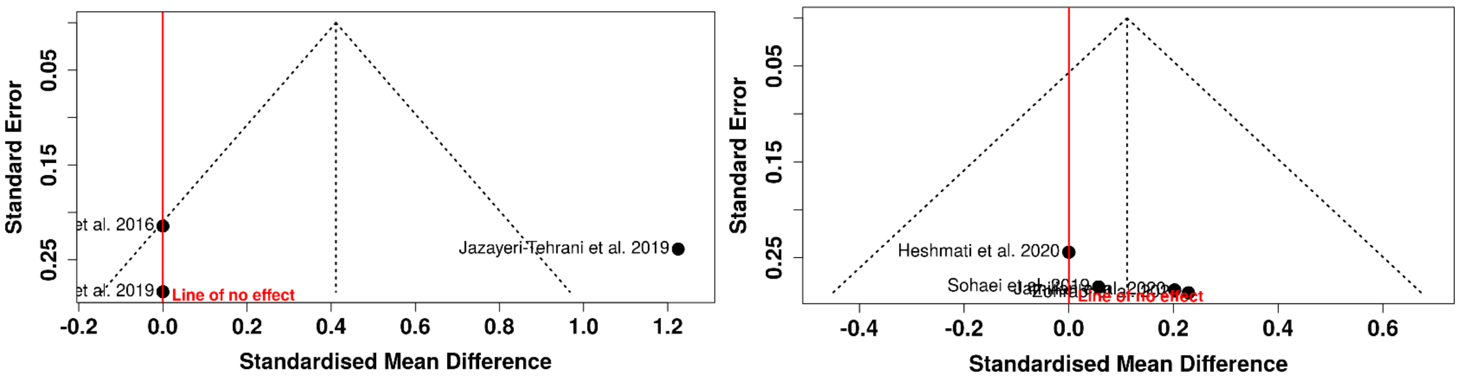


A

B

**Supplementary Figure S11b. Funnel plot analysis showing the clinical effects of consuming curcumin/*Curcuma longa* extracts compared to the control group on QUIKI (NAFLD (A) and PCOS (B)) in RCTs.**

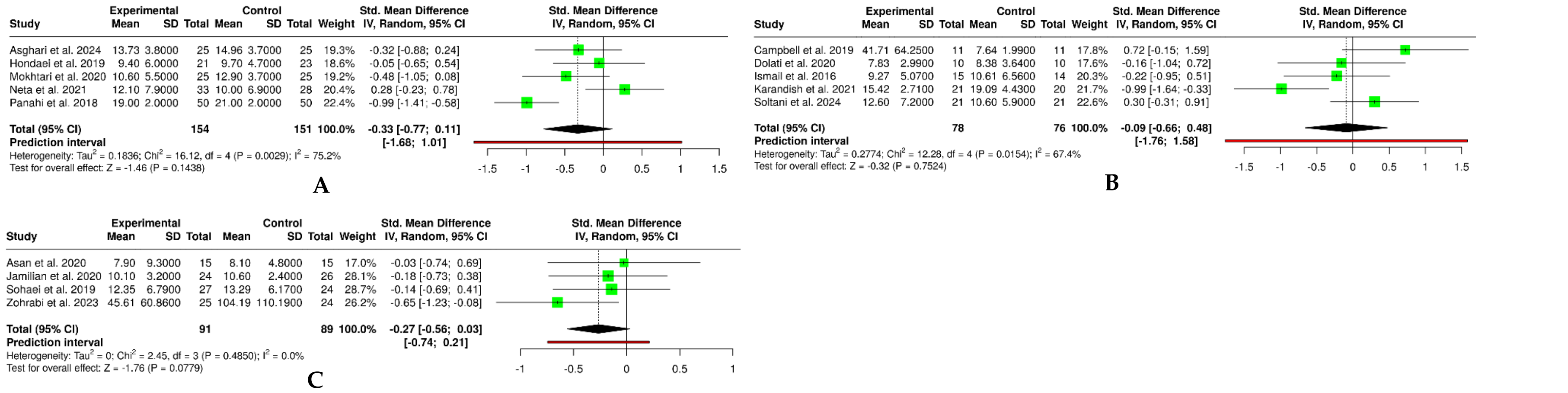


**Supplementary Figure S12a. Forest plot analysis showing the clinical effects of consuming curcumin/*Curcuma longa* extracts compared to the control group on Insulin (Diabetes (A), Obese (B), and PCOS (C)) in RCTs.**

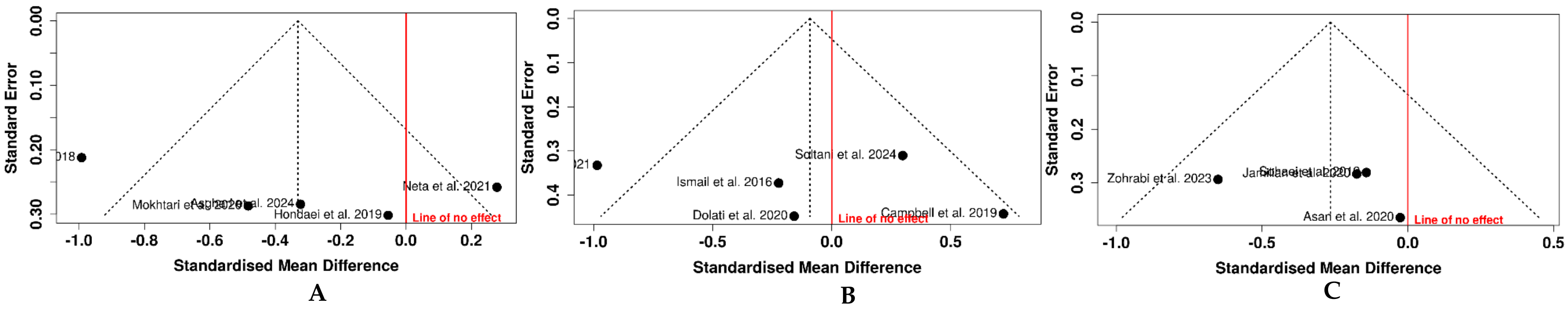


**Supplementary Figure S12b. Funnel plot analysis showing the clinical effects of consuming curcumin/*Curcuma longa* extracts compared to the control group on Insulin (Diabetes (A), Obese (B), and PCOS (C)) in RCTs.**

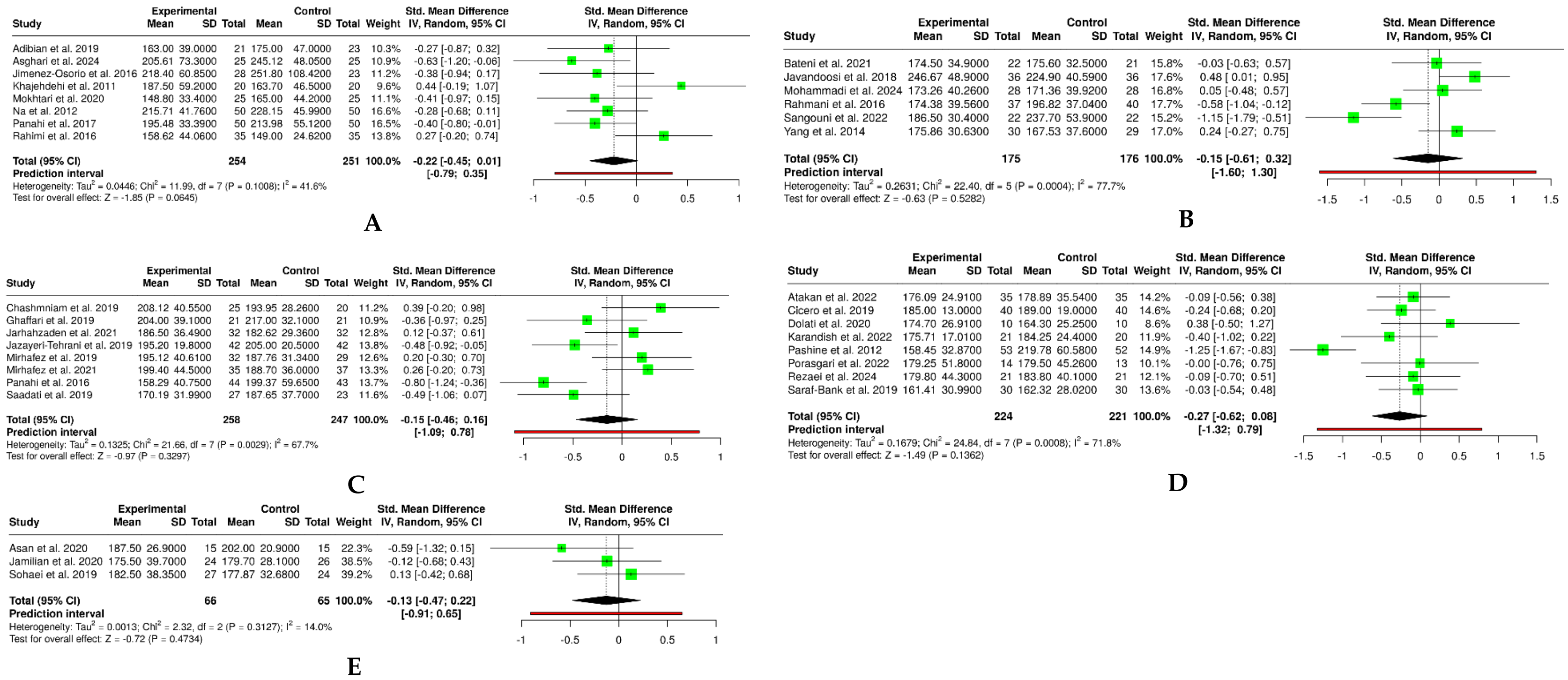


**Supplementary Figure S13a. Forest plot analysis showing the clinical effects of consuming curcumin/*Curcuma longa* extracts compared to the control group on TC (Diabetes (A), Mes (B), NAFLD (C), Obese (D), and PCOS (E)) in RCTs.**

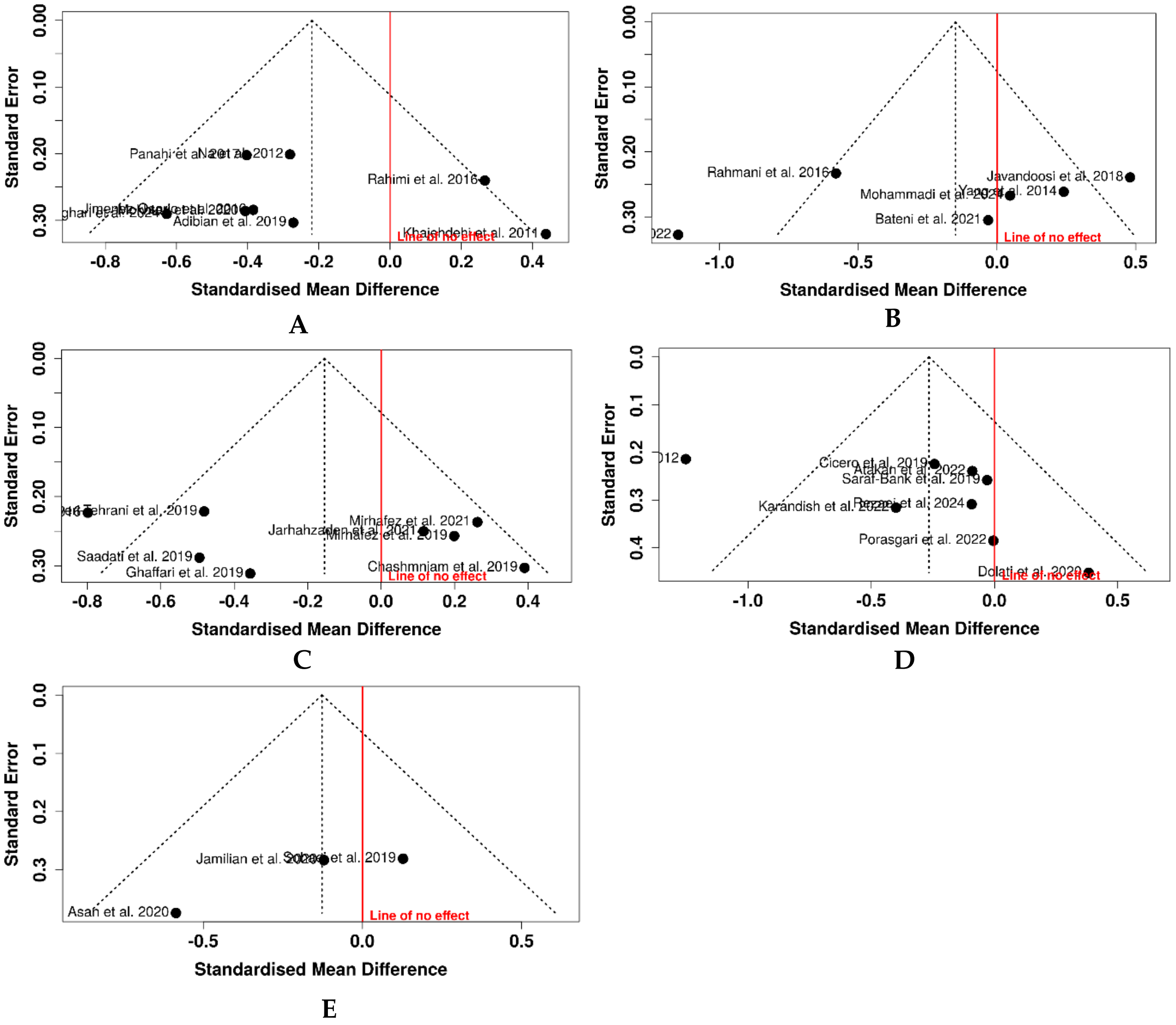


**Supplementary Figure S13b. Funnel plot analysis showing the clinical effects of consuming curcumin/*Curcuma longa* extracts compared to the control group on TC (Diabetes (A), Mes (B), NAFLD (C), Obese (D), and PCOS (E)) in RCTs.**

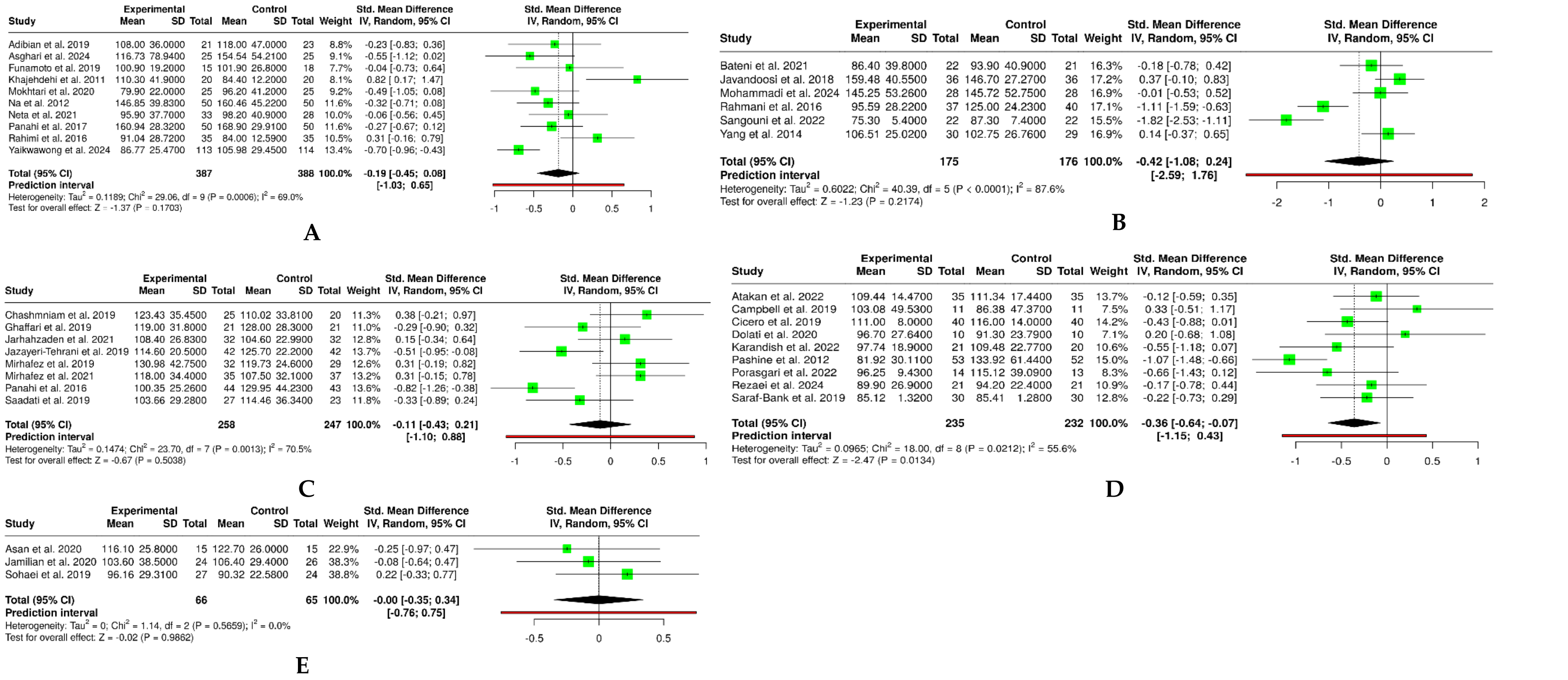


**Supplementary Figure S14a. Forest plot analysis showing the clinical effects of consuming curcumin/*Curcuma longa* extracts compared to the control group on LDL (Diabetes (A), Mes (B), NAFLD (C), Obese (D), and PCOS (E)) in RCTs.**

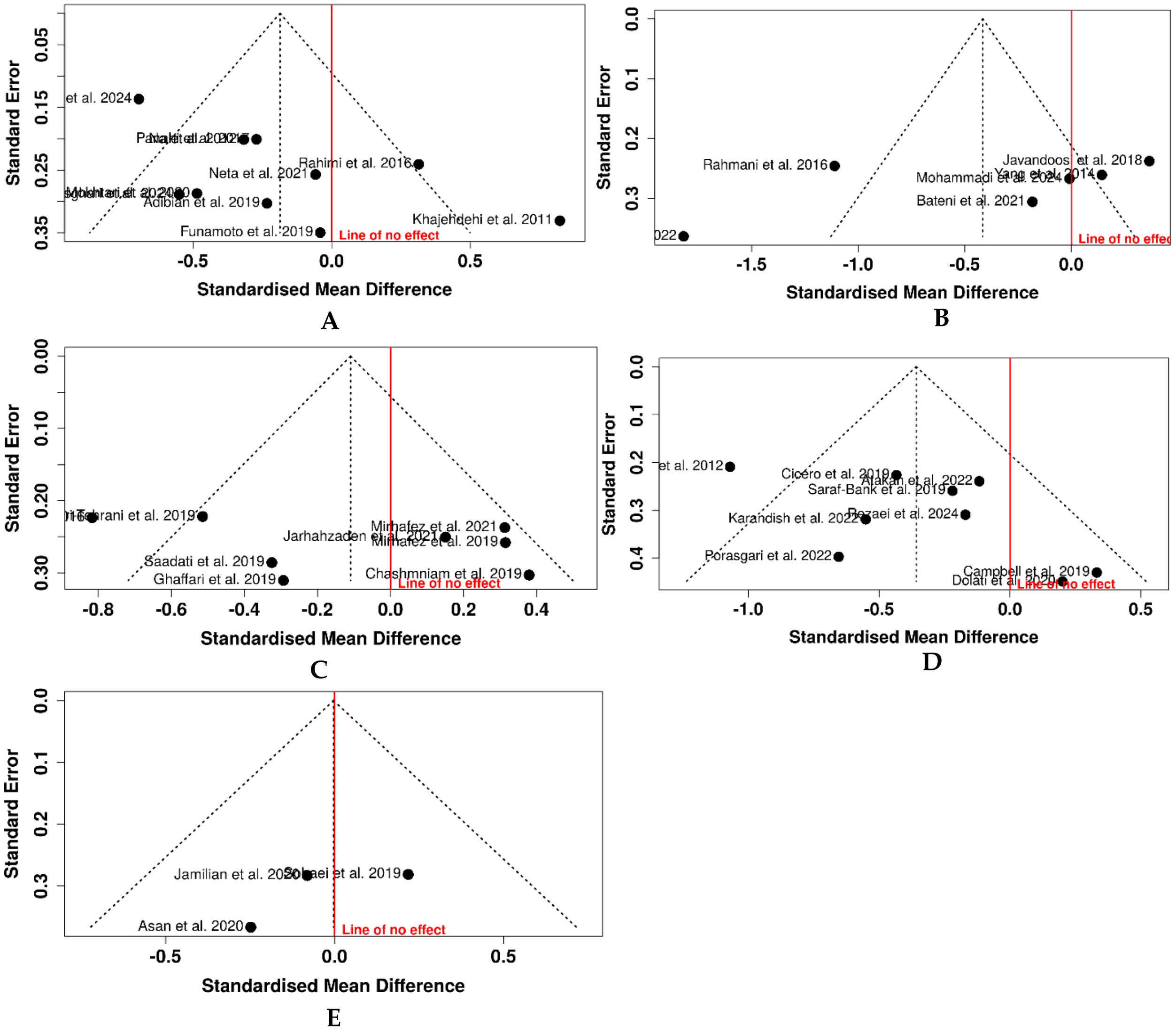


**Supplementary Figure S14b. Funnel plot analysis showing the clinical effects of consuming curcumin/*Curcuma longa* extracts compared to the control group on LDL (Diabetes (A), Mes (B), NAFLD (C), Obese (D), and PICO (E)) in RCTs.**

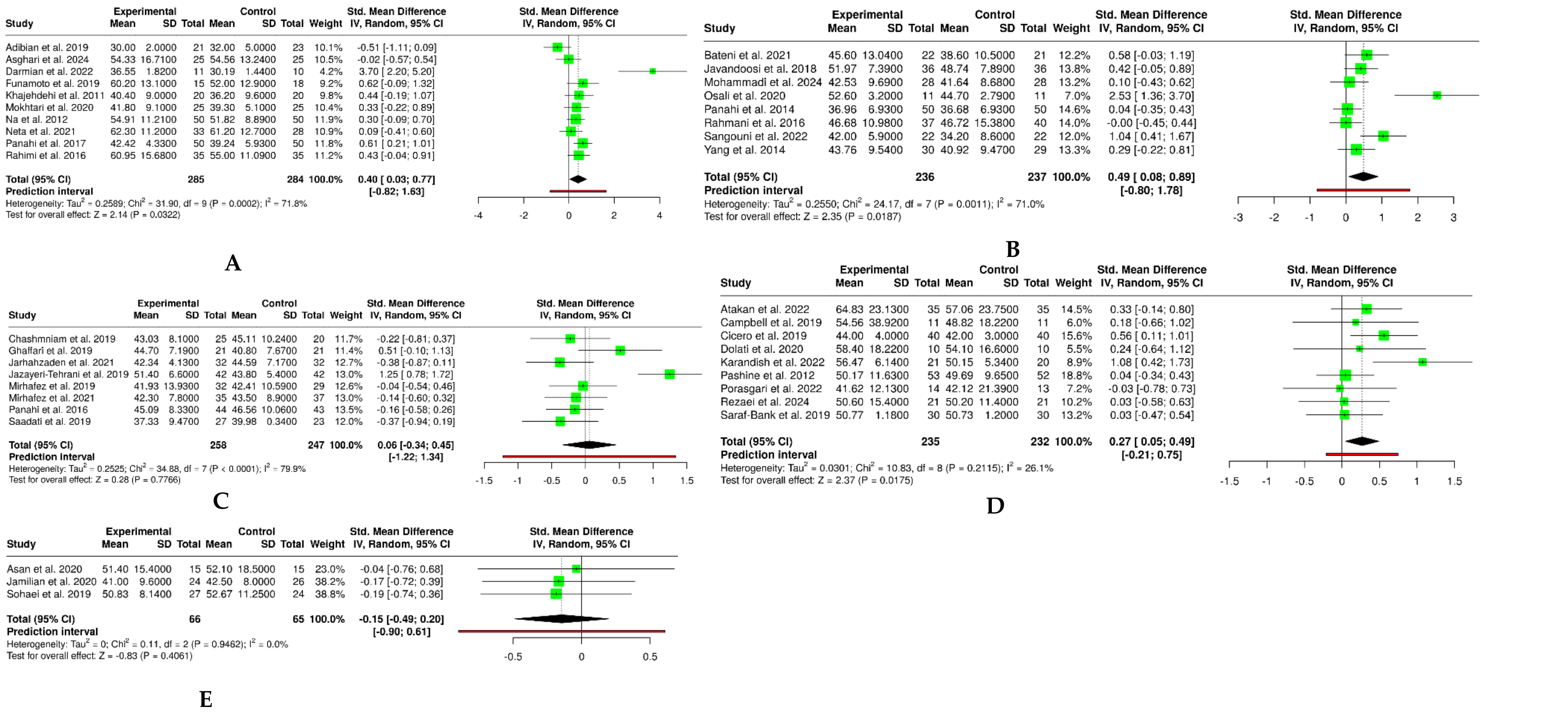


**Supplementary Figure S15a.** Forest plot analysis showing the clinical effects of consuming curcumin/*Curcuma longa* extracts compared to the control group on HDL (Diabetes (A), Mes (B), NAFLD (C), Obese (D), and PCOS (E)) in RCTs.

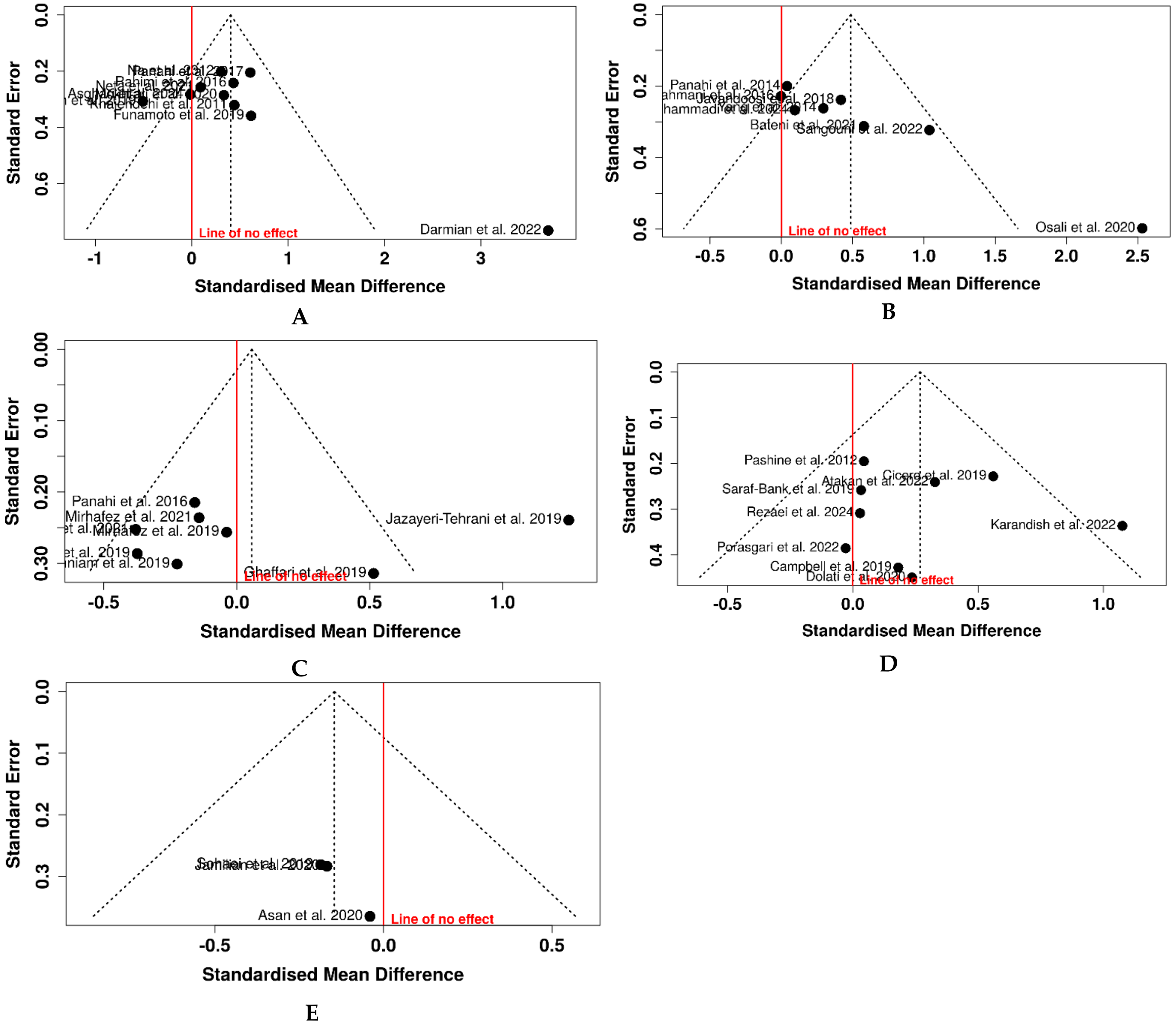


**Supplementary Fig. A15b. Funnel plot analysis showing the clinical effects of consuming curcumin/*Curcuma longa* extracts compared to the control group on HDL (Diabetes (A), Mes (B), NAFLD (C), Obese (D), and PCOS (E)) in RCTs.**

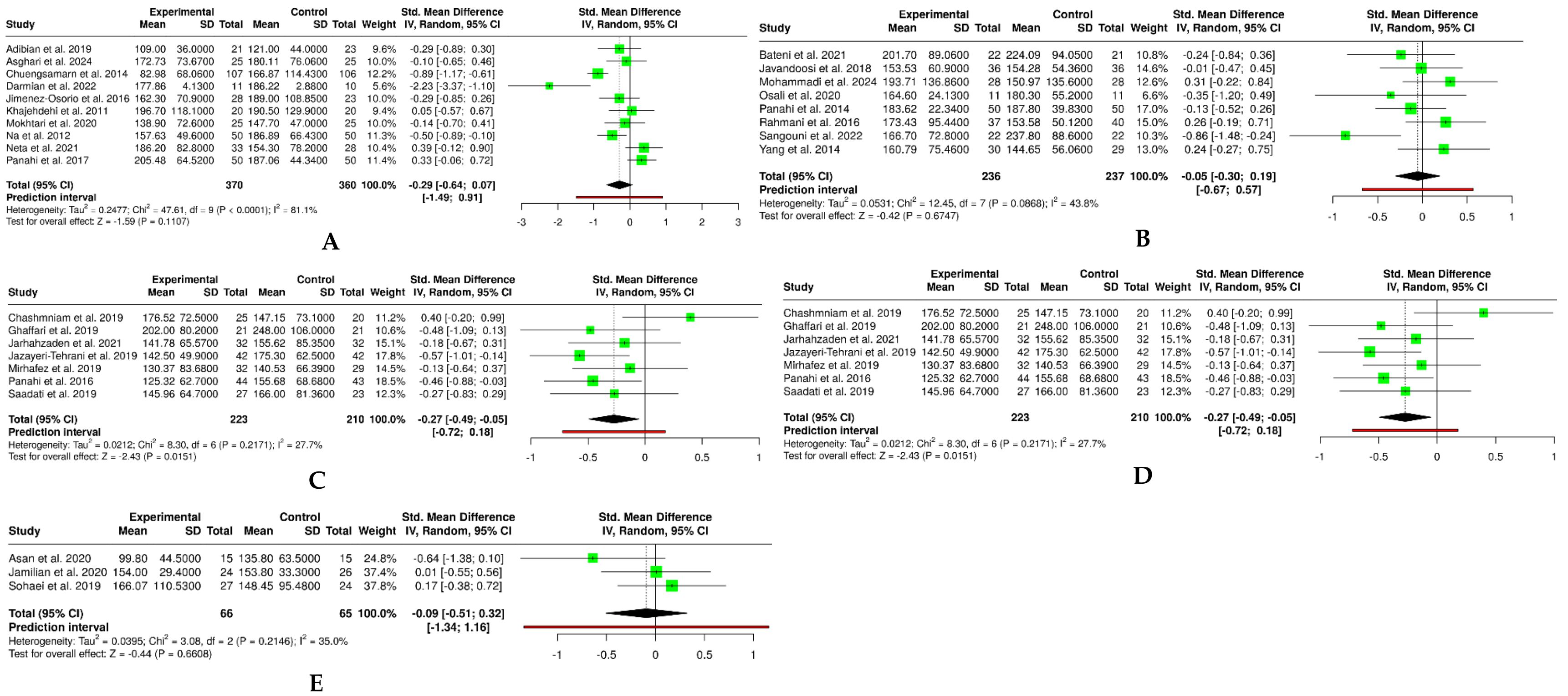


**Supplementary Figure S16a. Forest plot analysis showing the clinical effects of consuming curcumin/*Curcuma longa* extracts compared to the control group on TG (Diabetes (A), Mes (B), NAFLD (C), Obese (D), and PCOS (E)) in RCTs.**

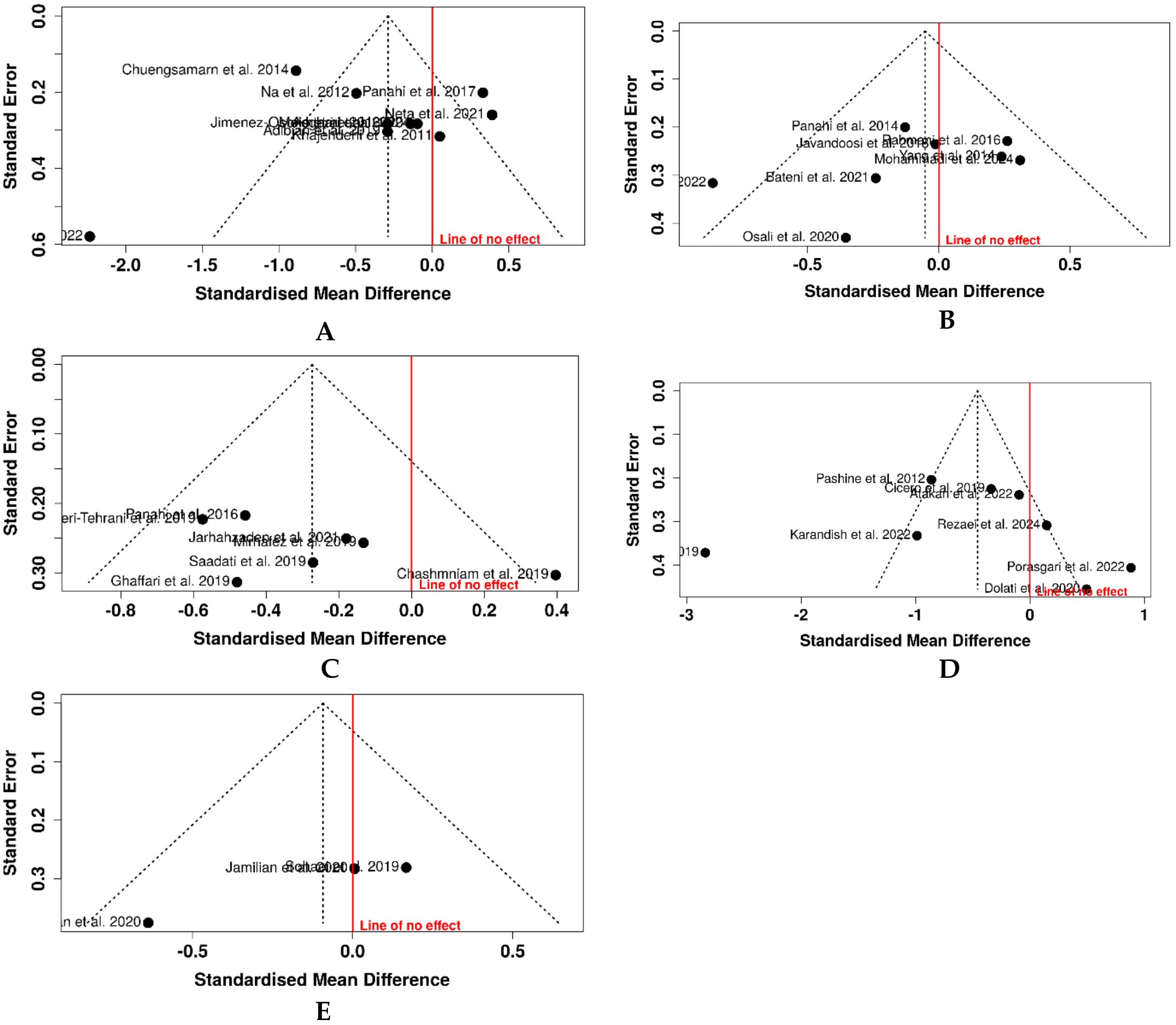


**Supplementary Figure S16b. Funnel plot analysis showing the clinical effects of consuming curcumin/*Curcuma longa* extracts compared to the control group on TG (Diabetes (A), Mes (B), NAFLD (C), Obese (D), and PCOS (E)) in RCTs.**

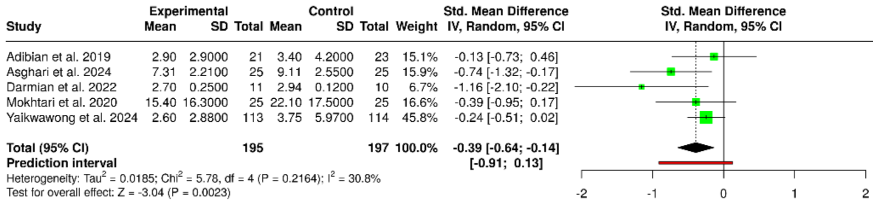


A

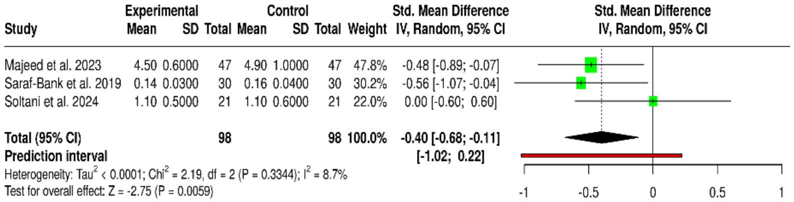


B

**Supplementary Figure S17a. Forest plot analysis showing the clinical effects of consuming curcumin/*Curcuma longa* extracts compared to the control group on CRP (Diabetes (A), Obese (B)) in RCTs.**

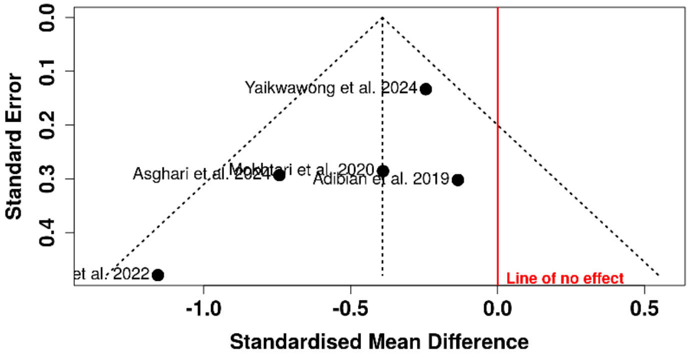


A

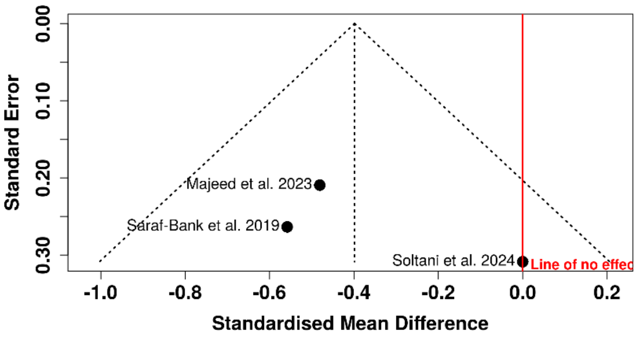


B

**Supplementary Figure S17b. Funnel plot analysis showing the clinical effects of consuming curcumin/*Curcuma longa* extracts compared to the control group on CRP (Diabetes (A), Obese (B)) in RCTs.**

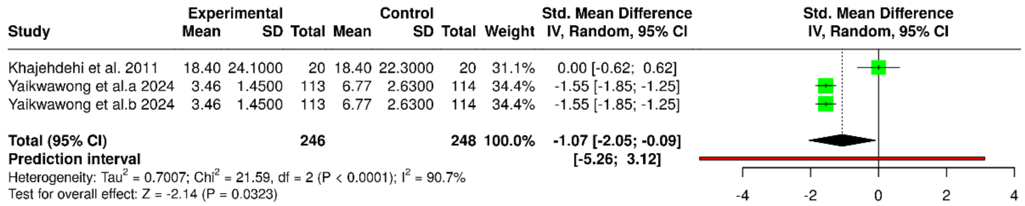


**Supplementary Figure S18a. Forest plot analysis showing the clinical effects of consuming curcumin/*Curcuma longa* extracts compared to the control group on TNF (Diabetes) in RCTs.**

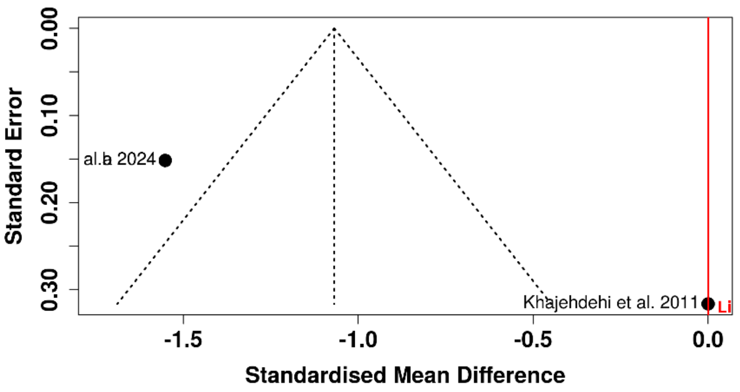


**Supplementary Figure S18b. Funnel plot analysis showing the clinical effects of consuming curcumin/*Curcuma longa* extracts compared to the control group on TNF (Diabetes) in RCTs.**

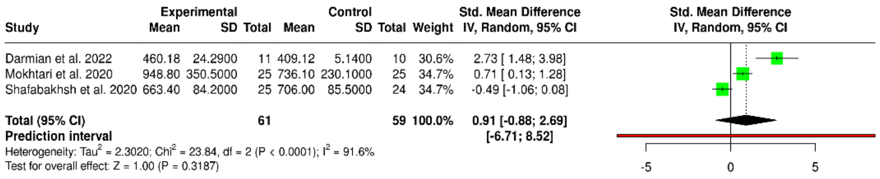


**A**

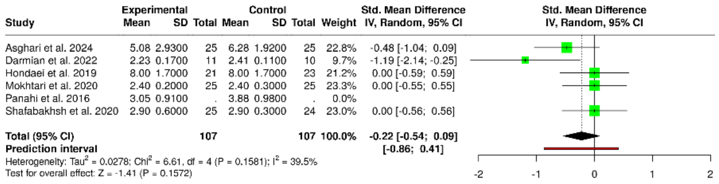


**B**

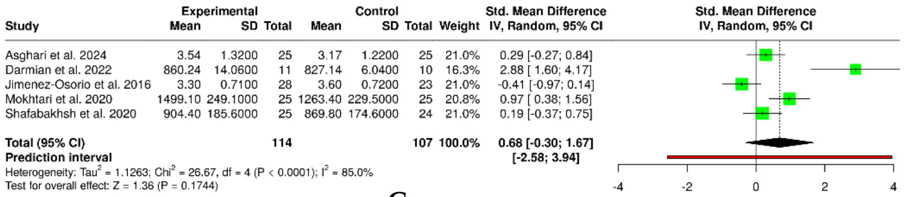


**C**

**Supplementary Figure S19a. Forest plot analysis showing the clinical effects of consuming curcumin/*Curcuma longa* extracts compared to the control group on Oxidative Stress (GSH (A), MDA (B), TAC (C)) in RCTs.**

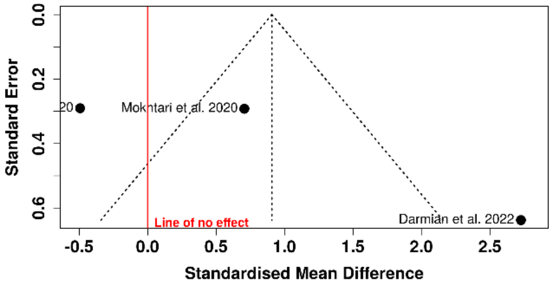


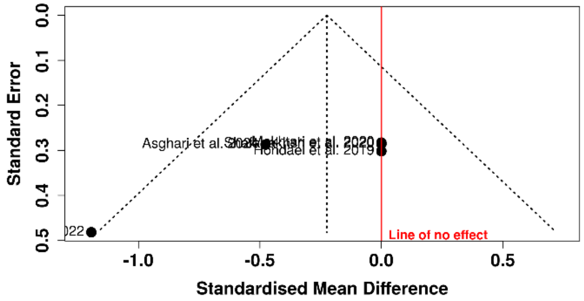


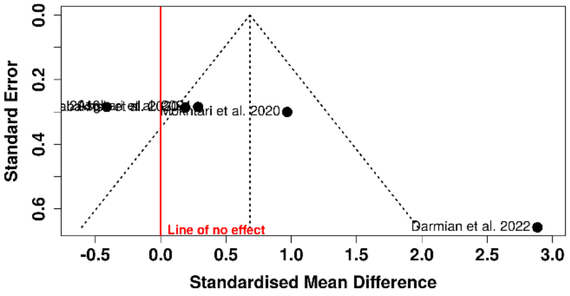


**A** **B** **C**

**Supplementary Figure S19b. Funnel plot analysis showing the clinical effects of consuming curcumin/*Curcuma longa* extracts compared to the control group on Oxidative Stress (GSH (A), MDA (B), TAC (C)) in RCTs.**
